# Apoplast proteome reveals that extracellular matrix contributes to multistress response in poplar

**DOI:** 10.1186/1471-2164-11-674

**Published:** 2010-11-29

**Authors:** Olga Pechanova, Chuan-Yu Hsu, Joshua P Adams, Tibor Pechan, Lindsay Vandervelde, Jenny Drnevich, Sara Jawdy, Ardeshir Adeli, Jeffrey C Suttle, Amanda M Lawrence, Timothy J Tschaplinski, Armand Séguin, Cetin Yuceer

**Affiliations:** 1 Department of Forestry, Mississippi State University, Mississippi State, MS 39762 USA; 2 Life Sciences and Biotechnology Institute, Mississippi Agricultural and Forestry Experiment Station, Mississippi State University, Mississippi State, MS 39762 USA; 3 W.M. Keck Center for Comparative and Functional Genomics, University of Illinois, Urbana, IL 61801 USA; 4 Environmental Sciences Division, Oak Ridge National Laboratory, Oak Ridge, TN 37831 USA; 5 USDA-ARS, Mississippi State, MS 39762 USA; 6 USDA-ARS, Fargo, ND 58105 USA; 7 Electron Microscopy Center, Mississippi State University, Mississippi State, MS 39762 USA; 8 Natural Resources Canada, Canadian Forest Service, Laurentian Forestry Centre, 1055 du P.E.P.S., P.O. Box 10380, Stn. Sainte-Foy, Quebec, Quebec G1V 4C7, Canada

## Abstract

**Background:**

Riverine ecosystems, highly sensitive to climate change and human activities, are characterized by rapid environmental change to fluctuating water levels and siltation, causing stress on their biological components. We have little understanding of mechanisms by which riverine plant species have developed adaptive strategies to cope with stress in dynamic environments while maintaining growth and development.

**Results:**

We report that poplar (*Populus *spp.) has evolved a systems level "stress proteome" in the leaf-stem-root apoplast continuum to counter biotic and abiotic factors. To obtain apoplast proteins from *P. deltoides*, we developed pressure-chamber and water-displacement methods for leaves and stems, respectively. Analyses of 303 proteins and corresponding transcripts coupled with controlled experiments and bioinformatics demonstrate that poplar depends on constitutive and inducible factors to deal with water, pathogen, and oxidative stress. However, each apoplast possessed a unique set of proteins, indicating that response to stress is partly compartmentalized. Apoplast proteins that are involved in glycolysis, fermentation, and catabolism of sucrose and starch appear to enable poplar to grow normally under water stress. Pathogenesis-related proteins mediating water and pathogen stress in apoplast were particularly abundant and effective in suppressing growth of the most prevalent poplar pathogen *Melampsora*. Unexpectedly, we found diverse peroxidases that appear to be involved in stress-induced cell wall modification in apoplast, particularly during the growing season. Poplar developed a robust antioxidative system to buffer oxidation in stem apoplast.

**Conclusion:**

These findings suggest that multistress response in the apoplast constitutes an important adaptive trait for poplar to inhabit dynamic environments and is also a potential mechanism in other riverine plant species.

## Background

Riverine ecosystems, characterized with rapid and dramatic environmental changes caused by fluctuating water levels and siltation, harbor a number of plants, including many species of the fast growing, ecological pioneers such as perennial poplar (*Populus *spp.; [1-5]). These species have a dramatic influence on ecosystem cycles. However, we have little understanding of how plants have adapted to such dynamic environments and cope with a variety of continually changing biotic and abiotic stressors while maintaining growth and development.

Leaves, stems, and roots in plants form an integrated physiological unit that receives stress signals and produces metabolic responses to control whole plant growth and development. Communication within the integrated physiological unit occurs through a continuum of symplast and apoplast [6]. Intracellular space of the symplast is composed of cellular cytoplasm connected through plasmodesmata. Apoplast, on the other hand, constitutes extracellular, aqueous space outside the plasma membrane including cell walls, spaces between cells, and xylem [6-8]. Thus, the apoplast represents a highly dynamic compartment serving as a continuum from roots through the stem to leaves and is potentially important as a bridge that perceives and transduces signals from the environment to the symplast.

Proteins secreted into apoplast might contribute to biotic and abiotic stress response as a first line of defense. A variety of these proteins have been identified in apoplast of several plants [8-20]. Such proteins predominantly represent functional categories associated with cell wall metabolism, defense, and programmed cell death. However, these studies have focused on annual plants that are not native to riverine ecosystems and have been conducted under controlled environments.

A few apoplast studies in perennial plants at the molecular level include xylem sap proteome from apple, peach, pear, poplar, as well as identification of the leaf apoplast antioxidative system in response to pox virus in plum [21-24]. Although these studies provide a basic understanding of protein composition in the apoplast of mainly horticultural species, they are limited in scope of the study, genomic resources (e.g., lack of whole genome sequence), protein source of tissues, developmental stages of trees (juvenile only), environmental conditions (mainly artificial controlled environments), and techniques. Consequently, there is a lack of a systems level understanding of the apoplast proteome and its function in riverine plant species. Thus, our goal was to elucidate a comprehensive and systems level apoplast proteome in riverine *P. deltoides *under normal conditions in addition to identifying apoplast defense mechanisms poplar has developed to respond to water and pathogen stress, which are among the most important stress factors in riverine ecosystems.

## Results and Discussion

### Extracting protein-containing poplar apoplast fluid

**Figure 1:**
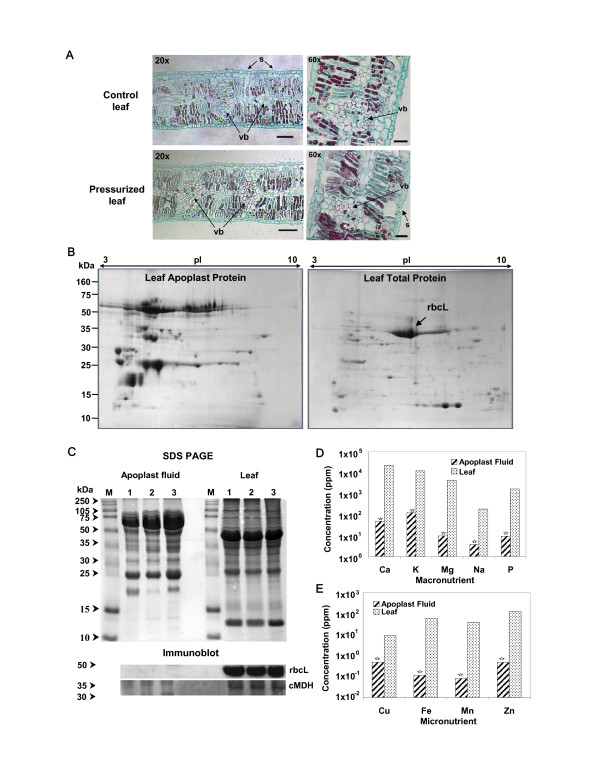
**Assessment of symplastic contamination of leaf apoplastic extracts**. (A) Light microscopy images from pressured and control *P. deltoides *leaves. S, stomata; vb, vascular bundle. Bar = 25 μm. (B) Protein distribution on 2-D PAGE from the extracts of leaf apoplast and whole leaf tissue. rbcL, the ribulose-1, 5-bisphosphate carboxylase/oxygenase large subunit. (C) Protein distribution on 1-D SDS PAGE and immunoblot analysis of symplastic rbcL and cytosolic malate dehydrogenase (cMDH) using the extracts from apoplast and whole leaf tissue. kDa, protein molecular weights. (D) Macro and (E) micro nutrients in leaf apoplast and whole leaf extracts. Asterisks indicate statistically significant (*p *< 0.001) difference in nutrients between apoplast and whole leaf extracts. Ppm, parts per million.

Obtaining a population of representative apoplast proteins from various tissues in poplar without symplastic contamination is technically challenging. We used a pressure-chamber method for leaves and a water-displacement method for stems to extract protein-containing apoplast fluid. To evaluate whether leaf apoplast extracts were contaminated with symplastic contents, we performed the following analyses as credible indicators of contamination. *First*, thin sections from pressurized and control leaves were examined using a light microscope. No ruptured cells were observed in the vicinity of stomata and within the vasculature (Figure 1A). *Second*, the 2-D PAGE protein profiles from leaf apoplast and whole leaf tissue were compared, and no similarity was observed in their protein patterns (Figure 1B). For example, the large subunit of ribulose-bisphosphate carboxylase (rbcL), which is symplast specific [25], was abundant in whole leaf tissues, whereas it was undetectable in the apoplast. *Third*, an immuno blot analysis was conducted for both symplast and apoplast protein samples. Using antibodies against the symplast proteins rbcL and malate dehydrogenase [25-27], both proteins were detected in whole-leaf tissue extracts, but not in apoplast proteome (Figure 1C). *Finally*, concentrations of macro and micro nutrients were significantly (*p *< 0.001) lower in leaf apoplast than in whole leaf extract (Figures 1D and 1E), which is consistent with previous findings [28]. We would have expected to observe similar amounts of nutrients in symplast and apoplast if the cell membrane had ruptured due to applied pressure. We can conclude from these observations that the extracted apoplast proteins from poplar leaves were devoid of symplastic contamination, confirming previous observations that pressurization of cotton (*Gossypium hirsutum*) and cocklebur (*Xanthium strumarium*) leaves for apoplast fluid extraction did not result in symplast contamination [28-30]. We did not verify whether water-pressure of stems to obtain apoplast proteins caused contamination of the apoplast content from symplast, because the applied water pressure by gravity was considerably less than the pressure applied to leaves via pressure chamber.

### Systems level protein composition of poplar apoplast

**Figure 2:**
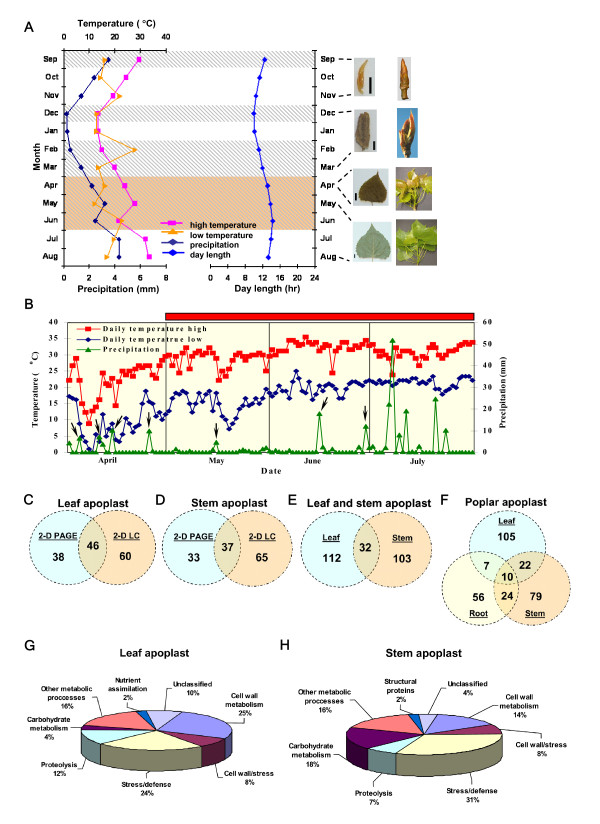
**Environmental conditions of the field where *P. deltoides *trees were located and quantitative distribution and functional classification of apoplast proteome**. (A) Average monthly high and low temperatures, precipitation, and day-length from 2003 to 2007. Orange-color shows the sampling time of tissues for apoplast proteome, whereas the shaded background indicates the sampling of leaf tissues for transcript analysis using microarrays. Developmental stages of leaves and shoots are shown on the right during sampling. Bar = 2 mm (September-March) and = 1 cm (April-August). (B) High/low temperatures and precipitation during sampling for apoplast proteome analysis (April-July, 2007). Arrows indicate flooding times. The red bar shows normal *Melampsora *infestation. (C-E) Quantitative distribution of leaf and stem apoplast proteins. 84 proteins (38+46) were detected with 2-D PAGE, whereas 106 proteins (60+46) were detected with 2-D LC. Both 2-D PAGE and 2-D LC detected 46 proteins. (F) Quantitative distribution of leaf, stem, and root apoplast proteins. 2-D PAGE-based poplar root apoplast proteome was from Dafoe and Constabel [22]. Functional classification of leaf (G) and stem (H) apoplast proteins.

To capture the most representative apoplast proteins in a growing season, we characterized the leaf and stem apoplast proteomes under normal conditions during a period (April-June) in which trees experienced active growth, transition, cessation of primary growth, and variation in temperature, precipitation, day length, and pathogen infestation (Figure 2A and 2B). Some of these factors are related and likely synergistic in a natural environment. Using both gel-based (2-D PAGE) and gel-free (2-D LC MS/MS) proteomic approaches, we identified 144 unique proteins in leaf apoplast (Figure 2C) and 135 unique proteins in stem apoplast (Figure 2D), totaling at 247 proteins (Figure 2E). A previously published poplar root apoplast data (gel-based only) contained 97 proteins [22], thus totaling the number of unique proteins in the root-stem-leaf apoplast continuum at 303 (Figure 2F). Leaf apoplast proteins were mainly associated with cell wall metabolism (most abundant), stress/defense, and proteolysis (Figure 2G and additional files 1, 2 and 3: Figure S1A, and Tables S1 and S2), whereas stem apoplast proteins were mainly associated with stress/defense (most abundant), cell wall metabolism, and carbohydrate metabolism (Figure 2H and additional files 1, 4, and 5: Figure S1B, and Tables S3 and S4). One major difference between the two proteomes was that a substantial portion of stem apoplast proteome (18%), but a small portion (4%) of leaf apoplast, was associated with metabolism of carbohydrates.

The SignalP [31] and SecretomeP [32] algorithms predicted that a substantial number of apoplast proteins (62% root, 57% stem, and 80% leaf) follow a secretory pathway (Additional files 2, 3, 4 and 5: Tables S1-S4; [22]), suggesting that they are secreted to the apoplast. Staining with a specific glyco-dye revealed that 25 proteins (30% of gel-based proteins) in leaf apoplast were glycosylated (Additional files 2 and 6: Table S1 and Figure S2A), which perhaps improves protein solubility, enhances thermal stability in hot summers (Figure 2B), provides protection from proteolysis, and modulates protein-protein interactions in apoplast [33-36]. Staining the gels with specific phospho-dye ProQ Diamond indicated that cysteine-rich repeat secretory protein 38 (POPTR_1698s00200.1) and thaumatin-like protein (POPTR_0018s10490.1) in leaf apoplast were potentially phosphorylated (Additional file 6: Figure S2B). This was supported with NetPhos 2.0 [37] predictions that there were six phosphorylation sites on thaumatin-like protein and 12 sites on cysteine-rich repeat secretory protein 38. These results indicate that the leaf-stem-root apoplast continuum in poplar contains diverse proteins that appear to be post-translationally modified and involved in important functions such as cell wall metabolism, stress/defense, and carbohydrate metabolism.

### Apoplast-controlled response to water stress

**Figure 3:**
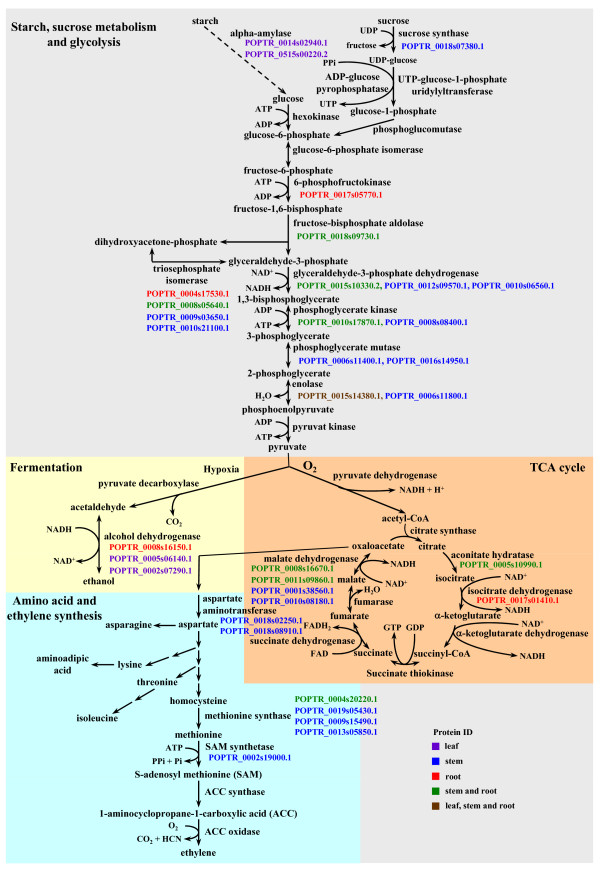
**Carbohydrate (starch and sucrose) catabolism via glycolysis, the TCA cycle, and fermentation**. Poplar leaf, stem, and root apoplast protein IDs are shown in colored numbers. The metabolic pathways were modified from Srivastava [138], Kreuzwieser et al [38], Magneschi and Perata [139], and Narsai et al [140].

Poplar often experiences flooding and drought stress in riverine ecosystems [1,3,4], and our experimental trees went through flooding and drought cycles (Figure 2B). To determine whether apoplast is involved in mediating water stress (flooding/hypoxia or drought) in poplar, we identified signature proteins associated with flooding and drought. Our leaf and previously published root [22] data contained alcohol dehydrogenases in addition to a number of important glycolytic enzymes in stem and root apoplasts (Figure 3). This indicates anaerobic respiration, and therefore some reliance of poplar on glycolysis and ethanolic fermentation for energy production.

**Figure 4:**
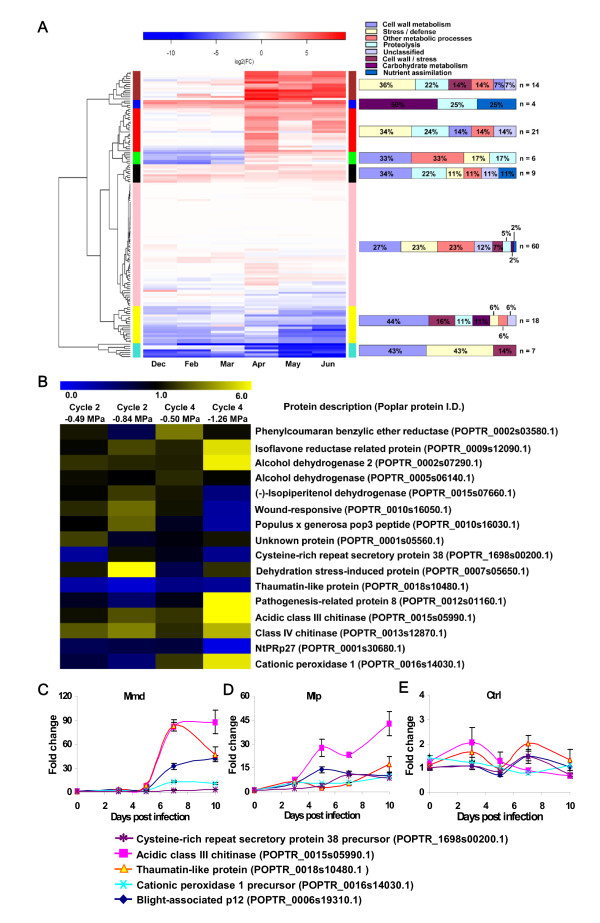
**Transcript levels of poplar leaf apoplast proteins and their transcriptional regulation by stress**. (A) Year-round transcript levels of *P. deltoides *leaf apoplast proteins using poplar microarrays, and hierarchical clustering of the transcripts corresponding to 139 proteins. Transcript abundance over time is represented as the log2 fold-change of each time point to the baseline time point (September). Vertical bars with a color represent a module. Functional classification of each module is shown on the right with the number (n) of genes. (B) Heat map showing response of genes corresponding to apoplast proteins at the transcript level to water stress in *P. deltoides *that was subjected to moderate (-0.49 MPa) to severe (-1.26 MPa) cyclical water stress. Expression less than 1.0 is down-regulated compared to control, whereas expression greater than 1.0 is up-regulated. (C-E) Transcript response of genes corresponding to apoplast proteins in leaves when poplar NM6 (*P*. *nigra *X *P*. *maximowiczii*) was challenged with the isolates of *M. medusae *f. sp. *deltoidae *(*Mmd*) and *M. laricipopulina *(*Mlp*). Control (ctrl) tissues were also analyzed. Error bars show standard deviation about the mean.

Although poplar is subject to frequent oxygen deficits (hypoxia) annually due to flooding and sedimentation, they show tolerance with minimal effect on growth and survival [1,3,5,38,39]. To maintain energy generation under flooding, trees switch mitochondrial respiration to alcoholic fermentation in roots and stems, resulting in significantly increased ethanol biosynthesis [38,40,41]. Unlike flood-intolerant tree species, ethanol does not accumulate in roots of flood-tolerant tree species (e.g., poplar) due to effective transport from roots in the apoplast to leaves where it is efficiently converted to acetyl-CoA via alcohol dehydrogenase and used in leaf metabolism [38,40,42-46]. Consistent with these previous studies, transcripts of alcohol dehydrogenase were abundant year-round in poplar leaves (Figure 4, additional files 7, 8 and 9: Figures S3 and S4B, and Table S5, black module). In contrast, alcohol dehydrogenase gene was induced upon flooding along with the activation of its protein in poplar roots [38,43]. Poplar leaves and roots contained different alcohol dehydrogenases (Figure 3), which phylogenetically fall into different clades (Additional file 10: Figure S5): POPTR_0005s06140.1 and POPTR_0002s07290.1 in leaf apoplast, POPTR_0008s16150.1 in root apoplast, and POPTR_0002s07290.1 in root transcriptome upon flooding. Thus, we hypothesize that poplar has evolved alcohol dehydrogenases optimized for ethanol biosynthesis in roots and for ethanol catabolism in leaves.

**Figure 5:**
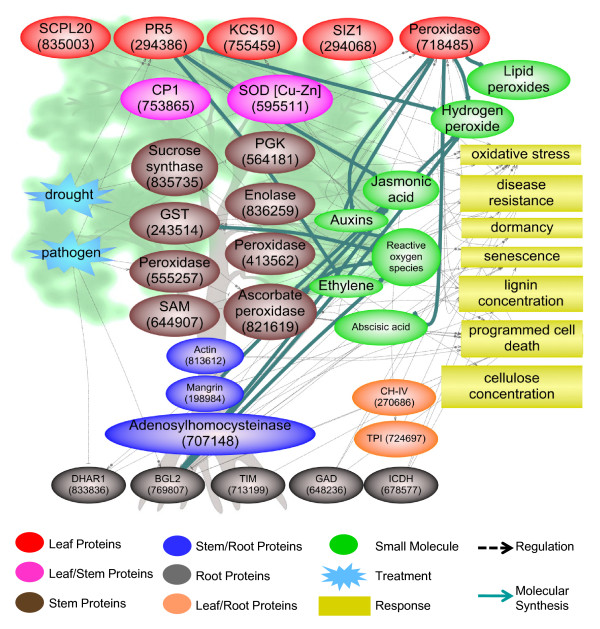
**Modeling leaf, shoot, and root apoplast proteomics data into regulatory networks and pathways**. The pathways include stress factors (drought and pathogen) upstream of proteins and physiological responses (yellow). Proteins are displayed on the background tree relative to the tissue in which they were found, except for two proteins (orange) that were found both in roots and leaves. Proteins displayed in pathways are identified by protein functional name/abbreviation followed by former poplar protein ID in parenthesis. Abbreviations in parenthesis: SCPL20 = serine carboxypeptidase-like 20, PR5 = pathogenesis-related gene 5, KCS10 = 3-ketoacyl-CoA synthase 10, SIZ1 = E3 SUMO-protein ligase, CP1 = cysteine-type peptidase, SOD = superoxide dismutase, PGK = phosphoglycerate kinase, GST = glutathione S-transferase, SAM = S-adenosylmethionine synthetase, CH-IV = class IV chitinase, TPI = triose-phosphate isomerase, DHAR = dehydroascorbate reductase, BGL2 = beta-1,3-glucanase 2, TIM = triosephosphate isomerase, GAD = glutamate decarboxylase, and ICDH = isocitrate dehydrogenase.

Sucrose and starch constitute the main carbohydrate reserves in poplar roots and stems [47], and a steady supply of carbohydrates from these reserves and leaves to hypoxic roots in poplar maintains alcoholic fermentation [38]. Carbohydrates appear to be effectively cycled within the leaf-stem-root continuum via symplast and apoplast in flood-tolerant tree species to meet the carbon demands of tissues [38,41,48]. Sucrose synthase (POPTR_0018s07380.1), an important regulator of sink strength in poplar [49], is present in stem apoplast (Figure 3 and 5, and additional files 2, 3, 4, 5 and 11: Tables S1-S4 and S6), indicating sucrose hydrolysis and a potential channeling into the glycolysis/fermentation pathway. Invertase, which is involved in the primary mechanism of aerobic sucrose hydrolysis, is suppressed under flooding, while sucrose synthase is upregulated to catabolize sucrose in poplar [38]. Supporting this view, a constitutively expressed alpha-amylase is present in leaf apoplast (Figure 3 and 4A, and additional file 9: Table S5, blue module) and is perhaps involved in starch hydrolysis after mobilization of carbohydrate storage reserves. During flooding, flood-tolerant tree species maintain low starch levels in leaves, but high starch levels in roots [42,50]. Increased sucrose synthase expression via overexpression in poplar was associated with maintaining height growth and biomass accumulation, as well as elevated levels of cellulose synthesis (Figure 5 and additional file 11: Table S6) and deposition in the xylem secondary cell wall, resulting in thick cell walls and improved wood density [49]. Indeed, sucrose synthase was among the highly expressed genes in poplar tension wood which is cellulose enriched, suggesting sucrose synthase involvement in stress response [51]. Anoxic rice plants [52] and hypoxic wheat roots [53] showed a high level of sucrose synthase activity and abundant cellulose deposition in the secondary cell walls of wheat, which was postulated to be important for increasing mechanical stability of roots under hypoxia. When the amount of sucrose synthase was reduced in roots of transgenic potatoes, roots were rapidly damaged by hypoxia and showed slow recovery when plants were returned to aerobic conditions [54]. Submerged leaves in flood water of tropical tree species that formed thick outer epidermal walls and a thick cuticle did not rot or detach from the plant during submergence [2], suggesting an improved capacity of those leaves to withstand the impact of flooding. Expression of sucrose synthase was induced by abscisic acid (ABA; Figure 5; additional file 11: Table S6; [55,56]), and abundant ABA was present in poplar leaves in the early growing season (Additional file 8: Figure S4B). However, a strong relationship between flooding and ABA signaling has yet to be established. Since flooding exerts stress, carbohydrates are also likely channeled via sucrose synthase to cellulose biosynthesis and its deposition into xylem secondary cell walls of poplar roots and stems for reinforcement.

#### Response to drought stress

As poplar displays an overall sensitivity to drought, it has evolved ways to cope with intensities and frequencies of water limitation in riverine ecosystems during summers [3,4]. To determine whether transcripts of stress-related apoplast proteins respond to water stress (drought), we applied moderate (-0.49 MPa) to severe (-1.26 MPa) cyclical water stress to *P. deltoides *in a controlled environment. Transcripts of apoplast proteins such as pathogenesis-related protein 8 (POPTR_0012s01160.1), acidic class III chitinase (POPTR_0015s05990.1), cationic peroxidase 1 (POPTR_0016s14030.1), isoflavone reductase-related protein (POPTR_0009s12090.1), and class IV chitinase (POPTR_0013s12870.1) accumulated in leaves of poplar upon water stress (Figure 4B and additional file 12: Table S7). Our year-round transcript analysis showed that genes encoding for pathogenesis-related protein 8 (red module), acidic class III chitinase (brown module), and cationic peroxidase 1 (brown module) were activated during the growing season (Figure 4A and additional files 7, 8 and 9: Figures S3 and S4C, and Table S5) when trees normally experienced drought (Figure 2A and 2B). These proteins are stress/defense related and appear to be secreted (Additional files 2, 3, 4 and 5: Tables S1-S4), perhaps suggesting their role in poplar apoplast to decrease the risk of pathogen and insect infestation under drought.

Our poplar apoplast data (Figure 5 and additional files 2, 3, 4, 5 and 11: Tables S1-S4 and S6) contained ascorbate peroxidase 1 (POPTR_0009s02070.1), methionine synthase (POPTR_0009s15490.1), mangrin/allene oxide cyclase (POPTR_0004s10240.1), heat shock protein 70-3 (POPTR_0010s21280.1), DnaK-type molecular chaperone hsp70 (POPTR_0008s05410.1), blight associated p12 (POPTR_0018s10730.1), and Cu-Zn superoxide dismutase (POPTR_0005s04590.1) that are responsive to drought (Additional file 12: Table S7). These proteins fall into the stress/defense functional category, except for methionine synthase which is involved in other metabolic processes. Heat shock proteins are considered as molecular chaperons involved in various processes including refolding of damaged proteins and protection against denaturation under drought [57]. The presence of methionine synthase and SAM synthase (Figure 3) indicates ethylene biosynthesis in poplar stem apoplast. Mangrin/allene oxide cyclase is involved in jasmonate biosynthesis [58], and existence of this enzyme in poplar root apoplast indicates the biosynthesis of jasmonate. Ethylene and jasmonate are widely involved in signaling biotic and abiotic stress (Figure 5 and additional file 11: Table S6). Ethylene likely plays a role in poplar shoot senescence (sacrifice of some of the current year's shoots) during hot and dry periods of low stream flow in summer that we often observed in our experimental *P. deltoides *trees. Shoot senescence is a common phenomenon in poplar [4] and perhaps reduces transpirational demand enabling the remaining shoots to maintain a favorable water balance.

### Apoplast-mediated response to biotic stress

Poplar has to cope with biotic stress, particularly the prevalent leaf fungal rust *Melampsora *in riverine ecosystems. *Melampsora *infects leaves via the apoplast [59]. To explore whether transcripts of stress-related apoplast proteins respond to pathogen challenges, we infected the poplar clone NM6 (*P*. *nigra *x *P*. *maximowiczii*) with *Melampsora medusae *f. sp. *deltoidae *(*Mmd*) and *Melampsora larici-populina *(*Mlp*). *Mmd *infection caused partial pathogen growth with macroscopic leaf necroses, whereas *Mlp *infection resulted in the formation of many uredinias on the leaf epidermis [60], suggesting that NM6 is more resistant to *Mmd*. Each transcript showed a distinctive profile over time after the infection (Figure 4C-E). The amplitude of gene expression was much higher following the *Mmd *infection, indicating an active defense response resulting in reduced fungal growth. Conversely, relatively weak or delayed gene expression occurred following the infection with *Mlp*. The difference perhaps reflects the variation in pathogenicity and host.

Transcripts of leaf apoplast proteins such as acidic class III chitinase, thaumatin-like protein, blight-associated protein p12, cationic peroxidase 1, and cysteine-rich repeat secretory protein 38 accumulated following *Melampsora *infection (Figure 4C-E). While the poplar leaf apoplast contained three different acidic class III chitinases (POPTR_0015s05990.1, POPTR_0014s08860.1, POPTR_0015s06000.1), only a single chitinase (POPTR_0015s05990.1) was detected in stem apoplast (Additional files 2, 3, 4 and 5: Tables S1-S4). Three different class IV chitinases (POPTR_0019s12360.1, POPTR_0013s12870.1, and POPTR_0019s12350.1) were also detected only in poplar leaf apoplast (Additional files 2, 3, 4 and 5: Tables S1-S4). Transcripts of acidic class III chitinase (POPTR_0015s05990.1) increased in abundance when poplar (*P. trichocarpa *x *P. deltoides *'Beaupre') was challenged with *M. larici-populina *(Additional file 12: Table S7). Moreover, transcripts encoding acidic class III chitinase (POPTR_0015s05990.1) and PR-8 (POPTR_0012s01160.1) rapidly increased in abundance when water stress (drought) was applied (Figure 4B and additional file 12: Table S7), suggesting a broader role of these proteins in stress response.

Chitinases and *β*-1,3-glucanases (POPTR_0016s05800.1, POPTR_0001s26210.1, POPTR_0006s04670.1, POPTR_0001s26210.1) were found together in poplar leaf and stem apoplast (Additional files 2, 3, 4 and 5: Tables S1-S4) and other plant species [11,14,15,17,21,24]. Chitinases are often co-induced with antifungal *β*-glucanases (PR-2 proteins), acting synergistically to limit fungal growth via degrading chitin and glucan of fungal cell walls [61-64]. These PR proteins likely mediate defense responses via activation of a signaling pathway through released elicitors from fungal cell walls into apoplast [65-68]. Furthermore, chitinases and *β*-glucanases appear to act synergistically with thaumatin-like proteins that can bind to *β*-1,3-glucans for hydrolysis [69,70]. The poplar thaumatin-like protein (POPTR_0018s10490.1) was present in two abundant isoforms in only leaf apoplast (spots 87 and 88; additional files 1 and 2: Figure S1A and Table S1). Genes of these thaumatin-like proteins were activated in response to infection of poplar leaves with *Mlp *(Additional file 12: Table S7). While transcripts of blight-associated protein p12 (POPTR_0006s19310.1) were activated by *Melampsora *infection (Figure 4C-E and additional file 12: Table S7), another poplar apoplast blight-associated protein p12 (POPTR_0018s10730.1) was upregulated by drought (Additional file 12: Table S7), suggesting that both family members are involved in stress response. Transcripts of these poplar apoplast proteins along with pathogenesis-related protein 8 (POPTR_0012s01160.1), hevamine (POPTR_0015s05980.1, a chitinase; [71]), and NtPRp27 (POPTR_0001s30680.1, POPTR_0009s09750.1) were abundant during the growing season and showed similar expression patterns (Figure 4A, additional files 7, 8 and 9: Figures S3, S4 D and S4E, and Table S5, red, brown, or green module), indicating co-regulation by water stress and pathogens. Some of these proteins and their homologs (e.g., chitinase and *β*-glucanase) in plants were regulated by ethylene (Figure 5 and additional file 11: Table S6; [13,67,72,73]) whose biosynthesis likely occurs in poplar apoplast (Figure 3). Thus, we postulate that poplar has developed an apoplast defense mechanism against pathogens by activating pathogenesis-related proteins under abiotic stress in riverine ecosystems.

### Cell wall modification in apoplast

As plant cell walls are modified in response to stress [74], we determined the extent to which apoplast proteins are involved in stress-induced cell wall modification in poplar. Eighteen different peroxidases (POX) were detected in poplar root, stem, and leaf apoplast, and virtually all of them were predicted to be extracellular (Additional files 1, 2, 3, 4, 5, and 13: Figure S1, Tables S1-S4, and Figure S6; [22]), indicating an abundant and diverse presence of peroxidases in the poplar apoplast. The poplar leaf apoplast POX gene (POPTR_0016s14030.1) was up-regulated in response to water stress (Figure 4B) and *Melampsora *infection (Figure 4C-E), implying a broader role in defense. The poplar leaf apoplast peroxidases (POPTR_0016s14030.1, POPTR_0014s14000.1) closely clustered (Additional file 13: Figure S6) with defense-related peroxidases POX8.1, POX22.3, POC1, pod2, pod4, PO2, POD1, and swpb4 [75-79]. Cationic peroxidase POC1 accumulated in the apoplast and cell walls of xylem vessels where it was involved in pathogen-induced lignification [80-82]. Lignin deposition in leaf cell walls was observed at infected sites following the interaction between *Melampsora *and poplar [59]. Moreover, transcripts of POPTR_0016s14030.1 and POPTR_0014s14000.1 showed a similar expression pattern and were only abundant during the growing season in poplar leaves (Figure 4A, additional files 7, 8 and 9: Figures S3 and S4C, and Table S5, brown module). These two peroxidases are likely involved in stress-induced lignification during the growing season in poplar leaf apoplast. Their role in defense may include reinforcement of cell walls to restrict pathogen invasion via oxidative cross-linking of monolignols, polysaccharides, and proteins such as proline-rich glycoprotein present in the poplar apoplast (POPTR_0006s18230.1; additional file 2: Table S1).

The majority of the stem apoplast peroxidases (POPTR_0005s14190.1, POPTR_0003s21640.1, POPTR_0003s21620.1, POPTR_0003s21610.1, POPTR_0001s05050.1, POPTR_0001s04850.1, and POPTR_0003s21660.1) that are all anionic (pI < 7) clustered together in the phylogenetic tree (Additional file 13: Figure S6). Some of these peroxidases in the same cluster were also detected in leaves (POPTR_0005s14190.1, POPTR_0001s05050.1, and POPTR_0003s21660.1) along with two leaf-specific (POPTR_0007s05100.1 and POPTR_0016s05860.1) or root-specific proteins (POPTR_0005s14190.1 and POPTR_0003s21660.1). Transcripts of four peroxidases (POPTR_0003s21660.1, POPTR_0149s00200.1, POPTR_0005s14190.1, and POPTR_0007s05100.1) showed year-round expression (Figure 4A, additional files 7 and 9: Figure S3 and Table S5, pink and yellow modules). *Melampsora *infection induced transcription of one particular peroxidase (POPTR_0001s04850.1) in poplar (Additional file 12: Table S7). The related peroxidases PO3, prxA3a, and PXP3-4 in poplar have been proposed to serve as key enzymes for lignifications [59,83-91]. Other poplar peroxidases in the same cluster (PO1 and PO2) are suberization-specific [90], and wound-inducible peroxidases are involved in suberization to help tissue healing [92]. Thus, we anticipate that these apoplast peroxidases are involved in lignification and suberization in poplar stems.

Four poplar apoplast peroxidases (POPTR_0015s00580.1, POPTR_0017s06550.2, POPTR_0007s02580.1, and POPTR_0004s14240.1) closely clustered (Additional file 13: Figure S6) with a poplar peroxidase (CWPO-C) that is proposed to play a role in lignification [88,89]. CWPO-C, whose transcripts are constitutively expressed in the developing leaf and shoot xylem, oxidizes sinapyl alcohol, a component of poplar lignin along with coniferyl alcohol. Transcripts of three peroxidases (POPTR_0017s06550.2, POPTR_0007s02580.1, and POPTR_0004s14240.1) were expressed during leaf development (Figure 4A, additional files 7 and 9: Figure S3 and Table S5, turquoise and yellow modules). One peroxidase (POPTR_0007s02580.1; 718485) appears to be responsive to drought and pathogens, as well as auxin and abscisic acid (Figure 5 and additional file 11: Table S6). As this particular peroxidase is an important hub, we predict that it plays an important role in mediating biotic and abiotic stress in poplar. Consequently, these four poplar apoplast peroxidases are probably involved in lignin polymerization in the apoplast of developing roots, leaves, or stems. Taken together, these observations indicate that poplar possesses a large number of peroxidases in the root-stem-leaf apoplast continuum to modify cell wall structure in response to stress.

### Antioxidative capacity of poplar apoplast

Metabolic reactions, stress-induced processes, and oxygen presence are expected to produce reactive oxygen species (ROS) such as superoxides and H_2_O_2 _in apoplast. Actively lignifying xylem tissues, particularly in stems during the growing season, sustain H_2_O_2 _production for cross-linking of lignin precursors by peroxidase involvement, increasing the apoplast oxidative load [83,93-96]. ROS were also increased in response to pathogen attack, leading not only to enhanced signal circulation that activates downstream antimicrobial proteins and phenolic compounds, but also to peroxidase-dependent cell-wall lignification, preventing pathogen penetration [97-101]. However, we do not have an understanding of how poplar counters oxidative stress in the apoplast.

Superoxide dismutase (POPTR_0011s01280.1, POPTR_0005s04590.1, and POPTR_0013s03160.1), ascorbate peroxidase (POPTR_0009s02070.1 and POPTR_0016s08580.1), monodehydroascorbate reductase (POPTR_0006s11570.1), and peroxiredoxin (POPTR_0019s04070.1) were present in poplar stem apoplast (Figure 5 and additional files 2, 3, 4, 5 and 11: Tables S1-S4 and S6). Two superoxide dismutases (POPTR_0011s01280.1 and POPTR_0005s04590.1) were the only antioxidant enzymes present in leaf apoplast (Additional files 2 and 3: Tables S1 and S2). Transcripts of one superoxide dismutase (POPTR_0005s04590.1) showed a year-round expression in leaves (Figure 4A, additional files 7 and 9: Figure S3 and Table S5, yellow module). These specialized antioxidant enzymes and low-molecular weight antioxidants such as ascorbate and glutathione are particularly involved in buffering oxidation [101-104]. Although none of these enzymes were detected in the poplar root apoplast [22], thioredoxin (POPTR_0005s25420.1) in the root apoplast might play a role in the regeneration of peroxiredoxin [105].

We anticipate that superoxide dismutase in poplar apoplast reduces superoxide ions into H_2_O_2 _which is then reduced to H_2_O by ascorbate peroxidase [96,106,107]. One of the superoxide dismutases (POPTR_0005s04590.1) was responsive to drought in poplar [108], while the other one (POPTR_0013s03160.1) was responsive to *Melampsora *(Additional file 12: Table S7). Knockdown of an extracellular high-isoelectric-point superoxide dismutase in poplar accelerated maturation, ROS accumulation, and lignification in the secondary cell walls with a number of developmental disturbances such as reduced growth, thinner stems, smaller leaves, and shorter and thinner xylem fibers and vessels [109], indicating the importance of superoxide dismutases in stress response and plant development. The presence of monodehydroascorbate reductase in the poplar apoplast indicates the regeneration of ascorbate for continuous removal of H_2_O_2_. Other antioxidant enzymes detected in stem apoplast include glyoxalase I (POPTR_0009s01280.1; additional file 4: Table S3) that removes methylglyoxal, a toxic by product of carbohydrate and amino acid metabolism [110,111], and glutathione S-transferase (POPTR_0483s00220.1 and POPTR_0002s20890.1; Figure 5 and additional files 4 and 11: Tables S3 and S6) that detoxifies xenobiotic compounds [112-114]. These observations suggest poplar has a robust apoplast antioxidative system.

## Conclusions

Our study shows that poplar has developed a diverse apoplast proteome in the leaf-stem-root continuum. Such a complex proteome appears to play a major role in mediating water, pathogen, and oxidative stress, suggesting that a systems level mechanism has evolved in poplar apoplast allowing encounters of multiple stresses while maintaining growth and development over many years. We anticipate our work to be a starting point for developing a systems level understanding of how the extracellular matrix mediates multistress responses in plant species of riverine ecosystems under fluctuating environmental conditions.

## Methods

### Apoplast fluid extraction

All sampled *Populus deltoides *trees were naturally growing along Sand Creek within the Tombigbee River system MS, USA (33° 27' 45" N; 88° 49' 12" W), for which weather data have been regularly collected and archived by Mississippi State University http://ext.msstate.edu/anr/drec/weather.cgi. Three sexually mature male trees (15, 25, and 30 years old) were selected for leaf apoplast analysis. We used a bucket truck with a hydraulic extending and elevating winch to reach the upper crown of ~25-m-tall trees. For stem apoplast analysis, twenty different male and female sexually immature trees with ages ranging from 3 to 6 years old were sampled. For leaves, a pressure chamber (Soilmoisture Equipment, Santa Barbara, CA) was used to extract apoplast fluid. The instrument contained a pressure-safe vessel attached to a pressure gauge. Current-year shoots with expanding and fully-expanded leaves (Figure 2A) were sampled separately from each tree in May-June, 2007. Shoots from each tree were collected between 8:00 AM and 9:00 AM daily (except weekends) and kept on ice. This fixed sampling time took into account that apoplast proteins and their corresponding transcripts might show circadian variation as many genes do in *Arabidopsis thaliana *[115]. In the laboratory, individual leaves were cut at the base of petiole, and cut surfaces were rinsed with sterile water. The leaf lamina was then placed into the pressure chamber with the petiole protruding outside. Using an ultra high purity compressed nitrogen gas, pressure was slowly increased within the chamber to 300 psi (2 MPa) at which apoplastic fluid started to exude. Pressure below 300 psi did not produce any extract. Exuding apoplast fluid was collected for one minute into a sterile tube that was placed on ice and contained 500 μl of cold two-times concentrated protein extraction buffer (1.8 M sucrose, 1 M Tris-base, 0.1 M Na_2_-EDTA, 0.2 M KCl, 4% *β*-mercaptoethanol, pH 8.7). The first drop of the extract was discarded to avoid contamination from broken cells at the cut site. Each tube was filled with 500 μl of apoplast fluid to make the final volume 1 ml. 100-200 μl of apoplastic fluid were extracted from each leaf. Collected extracts were immediately subjected to protein extraction. Three replications of apoplast fluid per tree were obtained in tubes for gel-based and gel-free protein analyses. Multiple tubes within a replication were pooled during protein extraction.

For stems, a water-replacement method was used to extract apoplast fluid. Stems of size 40-90 cm in length and 1-4 cm in diameter were collected between 8:00 and 9:00 AM in April-June, 2007 and kept on ice. In the laboratory, short sections (2.0-2.5 cm) of the bark at both stem ends were removed. The stem ends were then rinsed with deionized water. Plastic tubing was attached to the upper stem end over the exposed tissue and sealed with Parafilm to avoid leakage. The bark at the site of cut of the lower end of the stem was tightly wrapped with Parafilm to avoid contamination from phloem. Stems were positioned vertically, and deinonized water was applied to the tubing attached to the upper stem end. Exudate flowing from the bottom end was collected into sterile tubes placed on ice for several minutes. The first drop was discarded. Depending on the stem size, 1-5 ml of apoplast fluid mixed with water was collected from an individual stem. Apoplast fluid was filtered with sterile 0.2 μm syringe filters (Fisher Scientific, Pittsburgh, PA) to remove particulate matter, pooled, and either frozen at -80°C or immediately used for protein extraction. Three replications of apoplast fluid from all the stems were obtained in tubes. Pooled sap was concentrated on Amicon Centriplus YM-3 filters (Millipore Corporation, Bedford, MA) to 1/30 of original volume. Proteins were precipitated by adding four parts of 12.5% trichloroacetic acid and 0.1% *β*-mercaptoethanol in 100% acetone [116]. Precipitation was carried out at -80°C overnight. Proteins were sedimented by centrifugation at 6,000× *g *for 10 min, and the pellet was washed three times with ice-cold 80% acetone and once with 100% acetone and stored at -80°C.

### Protein extraction and gel-based protein analysis

Total proteins were extracted from apoplast fluid using a phenol-based procedure with modifications [117]. For each replication of protein extraction, apoplast fluid from five tubes was pooled, and five milliliters of Tris-saturated phenol (pH 8.0) were added. After 10 min of shaking, homogenate was centrifuged at 6,000× *g *for 20 min, and the phenol phase was carefully removed. Proteins were precipitated from phenol phase using five volumes of precipitation solution (0.1 M ammonium acetate and 1% *β*-mercaptoethanol in methanol). Precipitation was carried out overnight at -70°C. Precipitated proteins were collected by centrifugation at 6,000× *g *for 10 min and washed three times with cold precipitation solution and three times with cold 80% acetone. Each wash was followed by centrifugation at 6,000× *g *for 5 min. Following the last acetone wash, protein pellets were air-dried and stored at -20°C. Approximately 25-30 leaves were used to obtain 2.5 ml of apoplast fluid, which produced 200-250 μg of protein. The same protocol was used for extracting total proteins from whole leaves, except that two additional extractions of proteins from the phenol phase were carried out in order to remove interfering substances richly present in leaf tissue.

For resolving leaf apoplast proteins, two-dimensional polyacrylamide gel electrophoresis (2-D PAGE) was employed as previously described [118]. Protein spots were visualized by staining with Coomassie Brilliant Blue G-250 (Bio-Rad, Hercules, CA). Gels were scanned with VersaDoc 3000 imager (Bio-Rad) and analyzed with PDQuest (Bio-Rad). Three replications of 2-D gels were performed per tree, totaling nine gels. The landmark semi-automatic method was used to create a match set using all nine 2-D images, and the resulting master image was used for annotation of spots. Correct matching of all spots was manually inspected. The master image included protein spots present in all three poplar trees as well as spots that were present in at least two replications of gels per tree. Reproducible protein spots were subjected to downstream proteomic analyses.

For resolving stem apoplast proteins, the leaf apoplast protein procedure was followed. However, the amount of proteins (570 μg) was only sufficient for a large format 2-D gel (14 cm × 18 cm × 1.5 mm). Proteins were therefore visualized by Deep Purple Total Protein Stain (Amersham Biosciences, Piscataway, NJ). The gel was scanned using the Typhoon 9410 imager (Amersham Biosciences).

For protein spot picking, in-gel digestion, and MS/MS, we followed a previous procedure [118], except that protein identification was conducted by searching against the poplar database http://genome.jgi-psf.org/Poptr1_1/Poptr1_1.home.html. We were unable to identify three abundant protein spots in leaf apoplast (83, 87, and 88). Thus, we had to have N-terminal sequencing conducted by Cambridge Peptides [http://www.cambridgepeptides.com; additional file 14: Table S8]. The sequences were then searched against the poplar protein database using BLASTP, and the best matching similar protein was considered as the corresponding protein. The N-terminus sequence AGIAIYWGQNNN (spot 83) was present in two poplar proteins (POPTR_0015s05990.1 and POPTR_0012s01160.1). Thus, both proteins had to be included in our data set.

### Gel-free protein analysis

Two-dimensional liquid chromatography tandem mass spectrometry (2-D LC ESI/MS/MS) was used to analyze apoplast protein mixtures from leaves and stems. Frozen protein pellets were dissolved in 50 μl of 0.1 M ammonium bicarbonate and 4 M urea (Fraction 1). The remaining un-dissolved proteins were further dissolved in 50 μl of 0.1 M ammonium bicarbonate and 8 M urea (Fraction 2). Protein concentration was determined using 2-D Quant Kit (Amersham Biosciences). Aliquots of 200 μg of proteins were subjected to in-solution digestion. First, proteins were reduced by adding 1/10 volume of 50 mM dithiothreitol (DTT) and incubating for 1 h at room temperature. Alkylation was performed by adding 1/10 volume of 100 mM iodoacetamide and incubating for 30 min at 30°C in the dark. Before digestion, both protein samples (Fraction 1 and 2) were diluted (4X and 8X, respectively) with 25 mM ammonium bicarbonate to reduce urea concentration. HPLC-grade acetonitrile was added to each sample for a final concentration of 5%. Sequencing-grade modified trypsin (Promega, Madison, WI) was then applied to a final substrate/trypsin ratio of 50:1. Digestion was carried out at 37°C for 12-16 hours.

The peptide mixture was acidified with concentrated acetic acid and desalted with a peptide macro-trap according to manufacturer's instructions (Michron BioResources, Auburn, CA). Peptides were lyophilized with a SpeedVac (LABCONCO, model LYPH-LOCK 6, Kansas City, MO) and stored at -80°C. Immediately before mass spectrometry, the peptides were resuspended in 20 μl of 0.1% formic acid and 5% acetonitrile. Four replications of samples (both containing Fraction 1 and Fraction 2) were analyzed.

Following a previous procedure [119] with a slight modification, 2-D LC ESI/MS/MS was performed using ProteomeX Workstation (ThermoFinnigan, San Jose, CA) that included the Surveyor auto sampler and the Surveyor HPLC unit coupled directly in-line with LCQ Deca XP Plus - Electro Spray Ionization (ESI) ion trap mass spectrometer capable of MS/MS (in time) analysis. For the first dimension, peptides were separated on a strong cation exchange (SCX) BioBasic column (0.32 mm × 100 mm) (ThermoElectron, San Jose, CA). The following five-steps were used to elute peptides from SCX column: 0, 10, 23, 37, and 700 mM ammonium acetate (all in 5% acetonitrile and 0.1% formic acid). For the second dimension, peptides were loaded directly onto BioBasic C18 reverse phase column (100 mm × 0.18 mm) equilibrated with 0.1% formic acid and 5% acetonitrile. The following acetonitrile gradient in 0.1% formic acid was applied to elute the peptides: 5-10% for 1 min, 10-30% for 19 min, 95% for 7 min, 5% for 10 min. Total elution and spectra collection time was 37 min. The mass spectrometer Data Dependent method including dynamic exclusion was designed to have four scan events: one MS scan (m/z range: 300-1700) and three MS/MS scans of three most intense ions detected in MS scan. Collected spectra were processed with BioWorks 3.1 SR1 software (ThermoFinnigan). TurboSequest was used for protein identification by matching the experimental masses of parent and fragmented ions/peptides to those in the poplar protein database. The search parameters were that *1*] peptide mass tolerance (precursor ion) was set at 1.4 amu, *2*] two internal cleavage sites were allowed for trypsin, and *3*] group Scan was set at 7, minimum Group Count at 1, and minimum Ion Count at 15. Charge State and MSn level were set as Auto (peptide, 2.50; fragment ions, 0.00; ion series, B and Y). The following modifications were considered: C = 57.05 (differential) for carbamidomethylation of cysteins by iodoacetamide and M = 32.0 (differential) for oxidation of methionines. All accepted peptides had to be fully tryptic, had a ΔCn ≥ 0.08, and had the minimum cross correlation (Xcorr) value of 1.9 (+1 charge), 2.2 (+2 charge), and 3.75 (+3 charge). We only considered hits with at least one unique peptide as positive identifications. Single peptide identifications were accepted only if the peptide was a unique identifier and had occurred in all four replications of protein samples.

### Protein identification and sequence informatics

Sequences of matched/identified poplar proteins were further used in BLAST searches against UniProt Knowledgebase http://www.uniprot.org or the NCBI non-redundant database http://www.ncbi.nlm.nih.gov/blast using the BLOSUM62 algorithm. An E-value cut-off of 1.0 × e^-5 ^was used to find similar proteins in other plant species. A similar protein with the highest degree of sequence similarity was considered as the protein identity/similarity. If several poplar proteins matched to the same gel spot and their sequences produced the same BLASTP output (e.g., the same best matching similar protein, but with a different degree of sequence similarity to poplar protein), we only considered the hit with the highest Mascot score, which was always the 1^st ^rank hit. Identified poplar proteins were assigned putative biological function based on Gene Ontology Annotation [120] of their most similar proteins from other species or based on literature. For easy interpretation, the annotated proteins were then clustered into general biological categories that were identified based on common terminology used in literature. Poplar protein sequences were also screened for the presence of signal peptides using SignalP [31,121] and for non-classical signal peptide using SecretomeP [32]. Phosphorylation sites on proteins were predicted using NetPhos 2.0 [37]. Protein identifications were submitted to the Protein Identifications (PRIDE) database, accession numbers 14841-14844.

### Determining post-translational modifications

Five hundred micrograms of leaf apoplast proteins from one of the sexually mature *P. deltoides *trees were separated by 2-D PAGE as described above. After electrophoresis, gels were stained with Pro-Q Diamond and Pro-Q Emerald 488 to detect phosphorylated and glycosylated proteins, respectively. In both cases, manufacturer's (Molecular Probes, Carlsbad, CA) staining protocols were followed, and gels were scanned using the Typhoon 9410 imager. Gels were subsequently post-stained with SYPRO Ruby (Molecular Probes, Carlsbad, CA) for visualizing total proteins and re-scanned with the Typhoon 9410 imager.

### Microarray experiments

To determine year-round gene expression patterns corresponding to leaf apoplast proteins throughout leaf development, three independent replications of leaf tissues at each collection period were sampled from the upper crown of one of the sexually mature *P*. *deltoides *trees 2 h after sunrise and kept on ice. We used one genotype to have a uniform data set, because gene expression often significantly varies among poplar genotypes. We confirmed the results of our microarray experiments via qRT-PCR using two other sexually mature *P*. *deltoides *trees. Tissue collections were conducted in September 2005, December 2005, February 2005, March 2005, March 2006, April 2006, May 2006, and June 2006, spanning the development of leaves and four seasons, as well as overlapping with the timing of sample collection for apoplast proteome analysis. Leaves were preformed and enclosed in terminal vegetative buds in September, December, February, and March (Figure 2A; [122]). Leaves began unfolding from terminal buds late March, and this process continued by late May when leaves became fully expanded and mature (Figure 2A). Thus, we reorganized the samples based on leaf development, beginning with September and ending with June. We conducted array experiments in two 12-chip sets. The first set included September 05, December 05, February 05, and March 05, whereas the second set consisted of March 06, April 06, May 06, and June 06. Thus, the March samples (6 independent sampling) provided an overlap between the two sets. We used the poplar arrays from the same batch, and followed the same protocol executed by the same person.

Total RNA was isolated using a hot-borate extraction method [123], followed by DNase I digestion and clean-up using the RNeasy Mini Kit (Qiagen, Valencia, CA). A total of 3 μg of total RNA from each sample was used to synthesize the double-strand cDNA using the One-cycle cDNA Synthesis Kit (Affymetrix, Santa Clara, CA). After cleaning-up the double-strand cDNA using the Sample Cleanup Module (Affymetrix, Santa Clara, CA), the biotin-labeled cRNA was synthesized from the double-strand cDNA via *in vitro *transcription using the Genechip IVT Labeling Kit (Affymetrix). cRNA (20 μg) was fragmented at 94°C for 35 minutes. Fifteen micrograms of the fragmented cRNA were used to hybridize each Genechip Poplar Genome Array (Affymetrix) in Genechip Hybridization Oven 640 (Affymetrix) at 45°C for 16 hr. The arrays were washed using the Genechip Fluidics Station 450 (Affymetrix) and then scanned using the Genechip Scanner 3000 (Affymetrix). Microarray data were submitted to the National Center for Biotechnology Information Gene Expression Omnibus (GEO) GSE24349.

Quality control (QC) assessment, data processing, and statistical analysis of array data were conducted in R [124] using packages from the Bioconductor project [125]. QC assessment using affycoretools [126] showed that a block effect existed between the first set of samples (September, December, February, and March) and the second set of samples (March, April, May, and June) even after processing the arrays with the GCRMA algorithm [127]. In addition to background-correction and summarizing the multiple probes into one probe set value, GCRMA does a quantile normalization between arrays, but no normalization method can completely remove some block effects. In these cases, the block effect must also be accounted for in a statistical model. A one-way ANOVA for time was performed, taking the correlation due to block into account, using the limma package [128]. The limma model was fit using all 61,413 probe-sets on the array because it uses an empirical Bayes correction [129] that helps to improve power by borrowing information across genes. Expression values for each time point (adjusted for batch effects) were pulled for the model for the 139 probe sets matched to our leaf apoplast proteins of interest (see below).

Expression patterns over time were represented as the log2 fold-change of each time point to the baseline time point (September), and hierarchical clustering was performed on the 139 probe sets corresponding to 139 leaf apoplast proteins. To determine which of these clusters represented co-expressed modules, we used the "Dynamic Hybrid" algorithm from the dynamicTreeCut package [130]. A total of nine modules were found with deepSplit = 2. However, two of the modules had extremely similar expression patterns and were merged into one module (yellow, Figure 4A).

### Matching proteins to microarray probe sets

Affymetrix's poplar genome array contains over 61,000 probe sets that were designed from UniGene Build #6 (March 16, 2005; *Populus tremula *x *Populus tremuloides *only), GenBank^® ^mRNAs and ESTs (April 26, 2005; all *Populus *species), and the predicted gene set v1.1 from the *Populus *genome project (May 4, 2005; *P. trichocarpa*). The annotation information for the probe sets provided by Affymetrix is primarily based on UniGene and GenBank IDs, not DOE Joint Genome Institute's gene model names or protein/transcript IDs, because probe sets are based on the sequence data available at the time. However, our knowledge of which sequences actually uniquely represent a particular gene and which sequences may be common to related genes (i.e., annotation) is constantly changing. Therefore, there can be complex, many-to-many relationships between probe sets and Gene Models.

To find the "best" probe set on the array for each of our 144 proteins of interest from leaf apoplast, we used two other annotation sources: the PopARRAY database http://aspendb.uga.edu/poparray; February 4, 2009] and a custom annotation of the Affymetrix poplar array kindly provided by K-H. Han and J-H. Ko at Michigan State University (January 9, 2009). Using both sources, a total of 253 probe sets mapped to our 144 leaf apoplast proteins. We additionally used the PopARRAY database to reverse map the 253 probe sets to poplar gene models, and 212 mapped to only one gene model. We refer to these 212 as "unique-hit" probe sets whereas the other 41 are "multi-hit" probe sets. We used the following criteria to select one "best" probe set for each of our proteins. If one unique-hit probe set was found in both sources, we used it. If more than one unique-hit probe set was found in both sources, we used one with the lowest raw *p*-value from the ANOVA model. If no unique-hit probe set was found in both sources, we used the source that had a unique-hit probe set. If more than one unique-hit probe set in that source, we used the one with the lowest raw *p*-value from the ANOVA model. If no unique-hit probe sets were found in either source, we used multi-hit probe sets following the same criteria. Using these criteria, we were able to find unique-hit probe sets for 118 proteins, multi-hit probe sets for 21 proteins, and no matching probe sets for 5 proteins.

### Validation of microarray results

Validation of randomly selected seven genes in the microarray data was conducted via qRT-PCR using leaves from two other sexually mature *P. deltoides *trees (Tree 1 and Tree 2). Three replications of leaf tissues at each collection were sampled monthly from March to October 2 h after sunrise. Total RNA was isolated as previously described, and the first-strand cDNA was synthesized using 1 μg of total RNA in a total of 20-μl reaction mixture as described by Hsu et al [131]. qRT-PCR was conducted using the Power SYBR Green PCR Master Mix Kit (ABI, Applied Biosystems, Foster City, CA) and the 7500 Fast Real-Time PCR system (ABI, Applied Biosystems) with a run mode of "Standard 7500". Gene specific primers (Additional file 15: Table S9) were designed according to available sequences from the poplar database http://genome.jgi-psf.org/Poptr1_1/Poptr1_1.home.html. Since we used the primers on *P. deltoides *cDNA, the amplicons were sequenced and a BLAST analysis was conducted against the poplar genome database to confirm the gene IDs. The *P. deltoides UBIQUITIN *(*UBQ*) transcript was used as an internal standard. Each qRT-PCR reaction mixture contained 0.5 μl of cDNA template, 5 μl of SYBR Green Mix, 0.25 μl of 10 μM forward primer, 0.25 μl of 10 μM reverse primer, and 4 μl of ddH_2_O. The PCR was programmed to run an initial incubation at 95°C for 10 min, followed by 95°C for 15 sec and 60°C for 1 min for a total of 40 cycles. A dissociation curve analysis was conducted after each run to verify the specificity of amplicon and the formation of primer-dimers. A standard curve was generated by log [cDNA] (represented by the amount of total RNA used in the real-time reaction) versus the crossing point value using a series of dilutions of the first-strand cDNA. The ratio between the expression levels of each transcript and *UBQ *for each sample was calculated using the relative quantitative analysis method based on a formula for the standard curve assay. Each assay was repeated at least three times. Then, these expression data were compared with those first elucidated from our microarray analysis. Linear regression was used in which the qRT-PCR ratios (amount of target gene/amount of *UBIQUITIN*) were used as predictors of normalized microarray intensity values (transformed with log2) for each respective gene. This analysis was performed separately for Tree 1 and Tree 2.

### Water stress experiments and transcript analysis

*P. deltoides *'WV94' plantlets were transferred from tissue culture in November 2006 to leach tubes containing peat, vermiculite, and perlite in a 2:2:1 ratio, and initially fertilized with Osmocote, bone meal, gypsum, and dolomite at 12, 4, 4, and 0.5 ml/L, respectively. The transplants were grown in a mist bed for two weeks, and then transferred to greenhouse (26/21°C day/night) with a photoperiod of 14-16 h. After 12 weeks, plants were transplanted to 3.8 L containers using the same potting media, and placed in greenhouses (19°C/day with no supplemental lighting). Plants were fertilized with Peter's 20-20-20 at 300 ppm per month. Plants were grown until they reached an average height of 81.1 cm. A total of 20 plants were used for the cyclical water stress (drought) experiment, which commenced on 4/13/2007 and concluded on 5/14/2007. Water stress included 4 cycles in which water was withheld from plants for a period, and then watering was resumed. 10 plants were sampled after two cycles of drought, and 10 were sampled after 4 cycles of drought. Of the 10 plants, four were controls and six were water stressed. A completely randomized design was used in greenhouse. Pre-dawn water potential measurements were taken each night. During cycles 1 and 3, plants were watered again after water potential measurements indicated that plants were experiencing moderate water stress (< 0.05-1.0 MPa). During cycles 2 and 4, partially-expanded leaves were sampled when the water potential measurements indicated that the plants, on average, were under a moderate level of water stress. Water was then withheld from the plants until water potential measurements indicated that the plants were under severe water stress (< 1.0 MPa). Partially-expanded leaves were collected from each plant, and watering was resumed. The total number of samples collected after 4 cycles was 32 (4 control samples, 6 moderately-stressed samples, and 6 severely-stressed samples per cycle, i.e., cycle 2 and 4).

Total RNA was extracted from 32 partially expanded leaf samples, using the Spectrum™ Plant Total RNA Kit (Sigma). An on-column DNase I treatment (Sigma) was conducted during the RNA extraction according to the provided protocol. cDNA synthesis was conducted using a SuperScript^® ^III First-Strand Synthesis SuperMix for qRT-PCR (Invitrogen) according to the protocol provided. One microgram of total RNA was used for cDNA synthesis in a 20-μl reaction mixture. After cDNA synthesis, all cycle 2 control plants were pooled, all cycle 2 moderately-stressed samples were pooled, and all cycle 2 severely-stressed samples were pooled. The same was also done with the cycle 4 samples, resulting in a total of six samples for real-time qRT-PCR.

Following the selection of genes of interest based on the annotation of stress-related pathways from leaf apoplast proteome, qRT-PCR was used to compare the expression of 16 genes across the six samples. Amplification reactions (25.0 μl) were carried out using iQ™ SYBR^® ^Green Supermix according to instructions provided by Bio-Rad Laboratories. Each reaction contained a cDNA template (1.0 μl), SYBR^® ^Green supermix (12.5 μl), sterile water (8.5 μl), and the appropriate forward and reverse 5 μM primer pair (1.5 μl each). An actin gene (ACT) expressed at a constant rate across tissue types was used as a control to normalize the data for differences in input RNA and efficiency of reverse transcription between the samples. PCR amplification reactions were performed in triplicate. The thermal cycling conditions took place on the StepOne™ Plus Real Time PCR detection system (Applied Biosystems) and included 3 minutes at 95°C, 40 cycles of 95°C for 15 seconds, 55°C for 20 seconds and 72°C for 20 seconds, 1 minute at 95°C, 80 cycles at 55°C for 10 seconds with the temperature increasing by 0.5°C after each cycle and then a hold at 4°C until plates were removed from the machine. The forward and reverse primers that were used to detect gene expression are shown in additional file 15: Table S9.

Data analysis was carried out using StepOne™ Software (Applied Biosystems). Cycle threshold values that were flagged because they did not meet default QC requirements were removed from the analysis. Relative gene expression, or fold-change, was calculated for each gene with the cycle control sample used as the reference sample. Therefore, gene expression in the control always equaled 1. Values greater than 1 represent up-regulation, and values less than 1 represent down-regulation. Fold change minimum and maximum calculations were based on one standard deviation.

### Pathogen challenge experiments and transcript analysis

Individual trees of the hybrid poplar clone NM6 (*Populus nigra *X *Populus maximowiczii*) were initially obtained from *in vitro *grown plantlets and then transferred to a growth chamber with the following environmental conditions: 26°C/22°C day/night, 16-h day, 60% relative humidity, and 100 μmol m^-2 ^s^-1 ^light intensity. Plants were fertilized every other week with 20:20:20 N: P: K (1 g/L). Leaves LPI 8 and LPI 9 (leaf plastochron index, [132]) were used for all experiments from 1 m tall poplar trees (16 fully expanded leaves). Inoculation of leaf discs with *Melampsora *was conducted as previously described [133]. Briefly, disks of 2 cm in diameter were inoculated on the abaxial side with *M. medusae *f. sp. *deltoidae *or *M. laricipopulina *at a density of 1,000-3,000 spores (in 0.01% Tween) per cm^2^. After inoculation, the disks were kept on wet paper in large Parafilm sealed Petri dishes (22 cm × 22 cm) and incubated in a growth chamber at 18°C with a long photoperiod. Control disks were sprayed with Tween 0.01%. Samples for pathogen quantification and transcript analysis were collected at different time points over 10 days after inoculation. Tissues were collected from four biological replicate trees and processed separately for each treatment and time point.

Leaf disks were taken at specified intervals and frozen in liquid nitrogen. 100 mg of ground leaf-disk powder were used for either DNA or RNA extractions. Total genomic DNA preparation was performed by using the Plant DNeasy Kit (QIAGEN) following manufacturer instructions. Pathogen growth was quantified as described previously [133]. Amplifications were performed using a Stratagene Mx3000p apparatus (Agilent Technologies, Santa Clara, CA) and 1× QuantiTect SYBR^® ^Green mixture (Qiagen).

RNA was isolated using the Qiagen RNeasy Kit by following the manufacturer's instructions. RNA was treated with a DNase I, and complementary DNA was synthesized from the RNA samples (500 ng) for single strand conversion to cDNA using reverse transcriptase (Invitrogen, SuperScript™ II) and oligo-dT primers. Resulting samples were not treated with RNase H, and cDNAs were diluted at 5 ng/μl. We selected the genes of interest based on the annotation of stress-related pathways from leaf apoplast proteome. Gene specific primers (Additional file 15: Table S9) were designed according to available sequences from the NCBI Entrez Nucleotide database http://www.ncbi.nlm.nih.gov and from the poplar genome database. Amplifications were performed using a Stratagene Mx3000p apparatus (Agilent Technologies, Santa Clara, CA) and 1× QuantiTect SYBR Green mixture (Qiagen). Primer specificity was tested on NM6 genomic DNA (*P. trichocarpa, P. Nigra, P*. *maximowiczii*) and used at a concentration of 0.6 μM. Each PCR reaction had a 25-μl volume and contained 0.6 μM of forward and reverse primers, 10 ng of cDNA as template, and 1× master mix solution (Quanti Tect Tm Sybr^@ ^Green PCR Kit, Qiagen inc., Valencia, CA). Using the SYBR Green amplification mode in Stratagene's software, PCR cycling conditions were 15 min incubation at 95°C, followed by 40 successive cycles (94°C for 15 sec, annealing and extension between 57°C and 65°C for 2 min, depending on the primer set and target gene). Primer efficiency and number of molecules were determined using the No-Ct-Linear Regression of Efficiency (LRE) from fluorescence and Ct values [134]. Gene expression was presented as fold change observed between treatments relative to control samples based on a modified Livak and Schmittgen [135] calculation (2^-ΔΔCt^) to introduce efficiency (E). Internal control gene-stability measure was evaluated for poplar actin, elF4, ubi10, gadph, and cdc2 following a previously described normalization method (Additional file 16: Figure S7; [136]).

### Light microscopy

Tissues from pressurized and control leaves of three mature trees were fixed in half-strength Karnovsky's fixative (2% paraformaldehyde and 2.5% glutaraldehyde) with phosphate buffer (0.1 M, pH 7.2; [131]). Samples were then rinsed in distilled water and dehydrated in a graded ethanol series. Leaf pieces were infiltrated and embedded in Paraplast Plus (Oxford Labware, St. Louis, MO) using CitriSolve (Fisher Scientific, Houston, TX) as a transitional fluid. Serial 8 micron sections were cut with an American Optical 820 rotary microtome (Fisher Scientific, Houston, TX) and were stained with saffranin and fast green. All the thin sections were examined for ruptured cells in the vicinity of stomata and within the vasculature on an Olympus BX60 light microscope (Olympus America Inc., Center Valley, PA).

### Immunoblot analysis

Protein samples extracted from leaf apoplast fluid and whole leaf tissue of three sexually mature trees were resuspended in SDS-PAGE loading buffer [62.5 mM Tris-HCl, pH 6.8; 10% (v/v) glycerol; 2% (w/v) SDS; and 720 mM *β*-mercaptoethanol] and quantified with the 2-D Quant Kit (Amersham Biosciences). Fifty micrograms of total proteins from each sample were separated on SDS-PAGE using 4% stacking and 12% separating polyacrylamide gels. Protein samples were subsequently blotted onto a nitrocellulose membrane (Sigma, St. Louis, MO) using a semi-dried electro-transferring blotter (Owl Separation Systems, Portsmouth, NH). The rbcL (Ribulose-1,5-bisphosphate carboxylase/oxygenase large subunit) and malate dehydrogenase (cMDH) proteins, which are symplastic [25], were detected using the ECL Western Blotting Analysis System (Amersham Biosciences) according to the manufacturer's instructions. Monoclonal primary antibodies for rbcL (Cosmo Bio, Tokyo, Japan) were used in a dilution ratio of 1:5,000 for hybridization. A dilution ratio of 1:7,500 was used for cMDH (Santa Cruz Biotechnology, Santa Cruz, CA).

### Nutrient analysis

Three replications of 1.5 ml leaf apoplast fluid per sexually mature tree were used for nutrient analysis. Extracts were stored at -70°C until analysis commenced and were thawed immediately before use. Congruently, three samples of whole leaves were harvested from each tree for nutrient analysis. Leaf samples were dried at 50°C for 48 h and ground into a fine powder. Two hundred milligrams of leaf tissues were placed in a porcelain crucible, placed in a furnace, and ashed at 500°C for 4 h. The samples were then allowed to cool to room temperature. Ash was gently mixed with 1 ml of hydrochloric acid and deionized water solution (1:1 v/v) and allowed to dissolve for 1 h. Subsequently, liquid was decanted through 2 mm and 1 mm screens, respectively, for 1 h, and the solution was collected. Nutrient concentrations (Ca, K, Mg, Na, P, Cu, Fe, Mn, and Zn) from the whole leaf extract and the undiluted apoplast fluid were determined spectrophotometrically using an inductively coupled plasma (ICP) emission spectrophotometer (Thermo Jarrel Ash Iris Advantage ICP, Houghton, MI). There were three technical replications taken from each of the three biological replications. Concentrations were estimated for leaf (mg/g) and apoplast fluid (mg/l). Apoplast fluid density was estimated to be approximately one based on three mass measurements. Leaf and apoplast fluid nutrient measurements were then converted to a standard unit of parts per million (ppm). A mixed general linear model was used to compare the concentrations between the fixed tissue type effect and the random biological repeat (tree) effect.

### Abscisic acid analysis

Three independent leaf samples (#9-11 from the base of a shoot) from the upper crown of a sexually mature tree were collected 2 h after sunrise at each collection time from April to July in 2007. One gram (fresh weight) of leaves was ground into powder which was then freeze-dried using a SpeedVac (LABCONCO, model LYPH-LOCK 6). 100-150 mg (dry weight) of sample was used for determining the concentration of abscisic acid by following the procedure detailed in Destefano-Beltrán et al [137].

### Phylogenetic analysis

Full-length (predicted) alcohol dehydrogenase protein sequences were retrieved from the poplar database http://genome.jgi-psf.org/Poptr1_1/Poptr1_1.home.html. New poplar IDs were obtained from http://www.phytozome.net/poplar (Version 2.0 of the poplar assembly and annotation). *Arabidopsis *full-length (predicted) alcohol dehydrogenase proteins were obtained from the *Arabidopsis *database http://www.arabidopsis.org by conducting protein-protein BLAST using the protein sequence for poplar apoplast alcohol dehydrogenases. At2g47140 was used to root the tree. To construct the phylogenetic tree for peroxidases, protein sequences of peroxidases for other species were obtained from NCBI http://www.ncbi.nlm.nih.gov. Short sequences were excluded. For creating multiple alignments and constructing and visualizing the phylogenetic trees, we followed the procedure outlined in Hsu et al [131].

### Construction of regulatory networks and pathways

Unique poplar protein identification numbers from shoot and leaf apoplast were compiled with those from root apoplast [22]. These proteins (144 from leaf, 135 from stem, and 97 from root) were imported into Pathway Studio (Ariadne Genomics, Rockville, MD). These protein IDs were associated to homologs based on a poplar-to-*Arabidopsis *mapfile. Briefly, this file was created by blasting poplar proteins against the *Arabidopsis *genome. Appropriate homologs were selected based on best e-value and greatest similarity. After import, 165 proteins from root, stem, and leaf tissues were successfully matched with proteins described in the ResNet Plant Database 2.0. A pathway was then constructed based on established connections in ResNet Plant Database 2.0 with other proteins, small molecules, treatments, and plant processes. The initial pathway was extremely large and had many pathway branches not connected with physiological process of pertinence to our study. Thus, pathway thinning was conducted. *First*, plant processes not related to stress response were discarded. *Second*, connections were discarded if our proteins were not directly in the connection or upstream (e.g., direct connection between treatment and plant process). A *final *re-analysis of the pathway was conducted to ensure all objects were still connected to the main pathway. Any objects that became disconnected because of the previous two thinning regimes were subsequently deleted. Finally, proteins were positioned and color coded based on the tissue(s) in which they occurred.

## Authors' contributions

CY, OP, C-YH, JPA, JD, AA, JCS, TJT, AML, and AS designed research; OP, C-YH, JPA, TP, LV, JD, SJ, AML, AS, and CY performed research; OP, C-YH, JPA, TP, JD, AA, JCS, TJT, AS, and CY analyzed data; CY, OP, C-YH, and JPA wrote the paper. All authors read and approved the final manuscript.

## Supplementary Material

Additional file 1 **Supplementary Figure S1**. 2-D protein reference maps of *P. deltoides *leaf and stem apoplast. (A) Leaf apoplast proteins (1 mg) were resolved on 2-D PAGE and visualized with colloidal Coomassie Blue G-250 stain. Corresponding proteins in spots with numbers are given in additional file 2: Table S1. Protein molecular weights are shown in kilodaltons (kDa). (B) Representative 2-D reference map for stem apoplast proteome. Proteins were resolved on 2-D PAGE and visualized with Deep Purple Total Protein Stain. Corresponding proteins in spots with numbers are given in additional file 4: Table S3. Protein molecular weights are shown in kilodaltons (kDa). Click here for file

Additional file 2 **Supplementary Table S1**. Proteins identified in poplar (*P. deltoides*) leaf apoplast using 2-D PAGE MS/MS. Click here for file

Additional file 3 **Supplementary Table S2**. Proteins identified in poplar (*P. deltoides*) leaf apoplast using 2-D LC MS/MS. Click here for file

Additional file 4 **Supplementary Table S3**. Proteins identified in poplar (*P. deltoides*) stem apoplast using 2-D PAGE MS/MS. Click here for file

Additional file 5 **Supplementary Table S4**. Proteins identified in poplar (*P. deltoides*) stem apoplast using 2-D LC MS/MS. Click here for file

Additional file 6 **Supplementary Figure S2**. Staining of leaf apoplast proteins to identify post-translational modifications. (A) Leaf apoplast proteins stained with ProQ-Emerald 488 (left panel) to detect glycosylated proteins. The same gel was post-stained with SYPRO Ruby (right panel) to visualize all proteins. Boxed spots annotated with letters indicate glycosylated proteins whose IDs are given in additional file 2: Table S1. Protein molecular weights are shown in kilodaltons (kDa). (B) Leaf apoplast proteins stained with ProQ-Diamond (left panel) to detect phosphorylated proteins. The same gel was post-stained with SYPRO Ruby (right panel) to visualize all proteins. Boxed spots annotated with letters indicate phosphorylated proteins. *Box a *corresponds to cysteine-rich repeat secretory protein 38 (spot 14; POPTR_1698s00200.1; additional file 2: Table S1), whereas *box b *refers to thaumatin-like protein (POPTR_0018s10490.1; spot 88; additional file 2: Table S1). Protein molecular weights are shown in kilodaltons (kDa). Click here for file

Additional file 7 **Supplementary Figure S3**. Cluster analysis of year-round expression profiles of genes corresponding to 139 leaf apoplast proteins shown in Figure 4A. The log2 fold-change (FC) is shown on the left. Dotted lines (black) denote the expression profiles of individual genes, whereas the solid lines (red) represent the mean expression for the cluster. For each time point, boxplot representation of the expression profile for all filtered array elements is provided. Click here for file

Additional file 8 **Supplementary Figure S4**. Validation of microarray data via qRT-PCR using leaves from two other sexually mature *P. deltoides *trees (Tree 1 and Tree 2), predictive significance (*p*-value) and relationship strength (*R*^2 ^value) with microarray intensity values. (A) Alcohol dehydrogenase 2 (POPTR_0002s07290.1): For Tree 1, *p *= 0.007 and *R*^2 ^= 0.54; For Tree 2, *p *= 0.16 and *R*^2 ^= 0.19. (B) Abscisic acid (ABA) analysis in poplar (*P. deltoides*) leaves in April, May, June, and July. (C) Cationic peroxidase 1 (POPTR_0016s14030.1): For Tree 1, *p *= 0.164 and *R*^2 ^= 0.18; For Tree 2, *p *= 0.019 and *R*^2 ^= 0.44. (D) Thaumatin-like protein (POPTR_0018s10490.1): For Tree 1, *p *< 0.001, *R*^2 ^= 0.88; For Tree 2, *p *= 0.003 and *R*^2 ^= 0.59. (E) Blight-associated p12 (POPTR_0006s19310.1): For Tree 1, *p *= 0.06, *R*^2 ^= 0.30, For Tree 2, *p *= 0.016 and *R*^2 ^= 0.46. (F) Phenylcoumaran benzylic ether reductase (POPTR_0002s03580.1): For Tree1, *p*= 0.002, *R*^2 ^= 0.64; For Tree 2, *p *= 0.51 and *R*^2 ^= 0.042. (G) Dehydration stress-induced protein (POPTR_0007s05650.1): For Tree 1, *p *= 0.001 and *R*^2 ^= 0.65; For Tree 2, *p *< 0.001 and *R*^2 ^= 0.78. (H) Wound-responsive (POPTR_0010s16050.1): For Tree 1, *p *= 0.04, *R*^2 ^= 0.35; For Tree 2, *p *= 0.7 and *R*^2 ^= 0.014. Click here for file

Additional file 9 **Supplementary Table S5**. Clusters of year-round expression profiles of genes corresponding to 139 leaf apoplast proteins and their functional annotation. Click here for file

Additional file 10 **Supplementary Figure S5**. Phylogenetic analysis of alcohol dehydrogenase proteins in poplar (POPTR) and *Arabidopsis thaliana *(At). Poplar leaf (POPTR_0005s06140.1 and POPTR_0002s07290.1 in purple) and root (POPTR_0008s16150.1 in red) apoplast alcohol dehydrogenases fall into different clades. Click here for file

Additional file 11 **Supplementary Table S6**. Modeling leaf, shoot, and root apoplast proteomics data into regulatory networks and pathways via Pathway Studio using prior knowledge. This pathway contains treatments, primary proteins, effect of primary proteins, and secondary effect leading back to another protein or small molecule. Proteins are referenced by their protein functional name/abbreviation followed by poplar ID in parenthesis (old, new). Effect type is indicated by --+>, ---|, or --->, meaning a strong positive, strong negative, or unresolved association, respectively. Abbreviations: SCPL20 = serine carboxypeptidase-like 20, PR5 = pathogenesis-related gene 5, KCS10 = 3-ketoacyl-CoA synthase 10, SIZ1 = E3 SUMO-protein ligase SIZ1, CP1 = cysteine-type peptidase, SOD = superoxide dismutase, PGK = phosphoglycerate kinase, GST = glutathione S-transferase, SAM = S-adenosylmethionine synthetase, CH-IV = Class IV chitinase, TPI = triose-phosphate isomerase, DHAR = dehydroascorbate reductase, BGL2 = beta-1,3-glucanase 2, TIM = triosephosphate isomerase, GAD = glutamate decarboxylase, and ICDH = isocitrate dehydrogenase. Click here for file

Additional file 12 **Supplementary Table S7**. List of poplar apoplast proteins and corresponding transcripts and their expression levels in publicly available poplar (*Populus *spp.) microarray data obtained from stressed poplar tissues [59,60,141,142]. Dpi, Days Post Infection; Mlp, *Melampsora larici-populina*; Mmd, *Melampsora medusae *f. sp. *deltoidae*. Click here for file

Additional file 13 **Supplementary Figure S6**. Phylogenetic analysis of peroxidases from various plant species, including the poplar apoplast peroxidases. Protein sequences of 18 poplar apoplastic POXs and 30 deduced amino acid sequences for the following POXs from other species were used for phylogenetic analysis: *Arabidopsis *At5g06720 (*A. thaliana *AtPA2; Q42578), *Arabidopsis *At3g49120 (*A. thaliana *AtPCb; Q9SMU8), *Arabidopsis *At3g49110 (*A. thaliana *AtPCa; P24101), tobacco TP60 (*Nicotiana tabacum*; Q9XFL2), tomato TPX1 (*Lycopersicon esculentum*; Q07446), tomato TPX2 (*L. esculentum*; Q07445), cotton pod2 (*Gossypium hirsutum*; Q8RVW0), cotton pod3 (*G. hirsutum*; Q8RVP7), cotton pod4 (*G. hirsutum*; Q9XGV6), cotton pod6 (*G. hirsutum*; Q8RVP4), rice POX8.1 (*Oryza sativa*; O22439), rice POX22.3 (*O. sativa*; O22438), rice POC1 (*O. sativa*; Q9LKY9), Norway spruce SPI2 (*Picea abies*; Q9SC55), Norway spruce PX1 (*P. abies*; Q5W5I3), Norway spruce PX2 (*P. abies*; Q5W5I4), Norway spruce PX3 (*P. abies*; Q5W5I2), sweet potato swpa4 (*Ipomoea batatas*; B3SHI1), sweet potato swpb5 (*I. batatas*; B3SHI2), sweet potato swpb7 (*I. batatas*; B3SHI0), bell pepper PO2 (*Capsicum annuum*; A4ZCI6), poplar POD1 (*Populus alba × Populus tremula *var. *glandulosa*; Q58GF4), lombardy poplar CY26 (*P. nigra*; Q40949), western balsam poplar PXP3-4 (*P. trichocarpa*; Q43101), white poplar CWPO-C (*P. alba*; Q4ADU9), white poplar PO1 (*P. alba*: Q50KB0), white poplar PO2 (*P. alba*; Q08IT5), white poplar PO3 (*P. alba*; Q08IT6), aspen prxA1 (*P. kitakamiensis*; Q43055), aspen prxA3a (*P. kitakamiensis*; Q43049). *A. thaliana *L-ascorbate peroxidase 1, cytosolic (At1g07890/APX1; Q05431) was used to root the tree. Click here for file

Additional file 14 **Supplementary Table S8**. N-terminal sequencing of proteins from three spots (83, 87, and 88) on leaf apoplast 2-D gels. Letters correspond to amino acids. Dashes indicate undetermined residues. Click here for file

Additional file 15 **Supplementary Table S9**. List of primers that were used for qRT-PCR analyses. Click here for file

Additional file 16 **Supplementary Figure S7**. Using qRT-PCR, the transcript stability of potential internal control genes was determined using leaf tissues of the hybrid poplar clone NM6 (*P*. *nigra *X *P*. *maximowiczii*) challenged with the isolates of *M. medusae *f. sp. *deltoidae *(Mmd) and *M. laricipopulina *(Mlp). Click here for file
