# Supplementary material for: Apoplast proteome reveals that extracellular matrix contributes to multistress response in poplar
Source: BMC Genomics. 2010 Nov 29;11:674. doi: 10.1186/1471-2164-11-674 (PMC3091788; doi:10.1186/1471-2164-11-674)
Supplement: Additional file 1 — Supplementary Figure S1. 2-D protein reference maps of P. deltoides leaf and stem apoplast. (A) Leaf apoplast proteins (1 mg) were resolved on 2-D PAGE and visualized with colloidal Coomassie Blue G-250 stain. Corresponding proteins in spots with numbers are given in additional file 2: Table S1. Protein molecular weights are shown in kilodaltons (kDa). (B) Representative 2-D reference map for stem apoplast proteome. Proteins were resolved on 2-D PAGE and visualized with Deep Purple Total Protein Stain. Corresponding proteins in spots with numbers are given in additional file 4: Table S3. Protein molecular weights are shown in kilodaltons (kDa). [file 1471-2164-11-674-S1.PDF]

Additional file 1

File format: PDF

Title: Supplementary Figure S1

Description:
Figure S1. 2-D protein reference maps of *P. deltoides* leaf and stem apoplast. (A) Leaf apoplast proteins (1 mg) were resolved on 2-D PAGE and visualized with colloidal Coomassie Blue G-250 stain. Corresponding proteins in spots with numbers are given in additional file 2: Table S1. Protein molecular weights are shown in kilodaltons (kDa). (B) Representative 2-D reference map for stem apoplast proteome. Proteins were resolved on 2-D PAGE and visualized with Deep Purple Total Protein Stain. Corresponding proteins in spots with numbers are given in additional file 4: Table S3. Protein molecular weights are shown in kilodaltons (kDa).

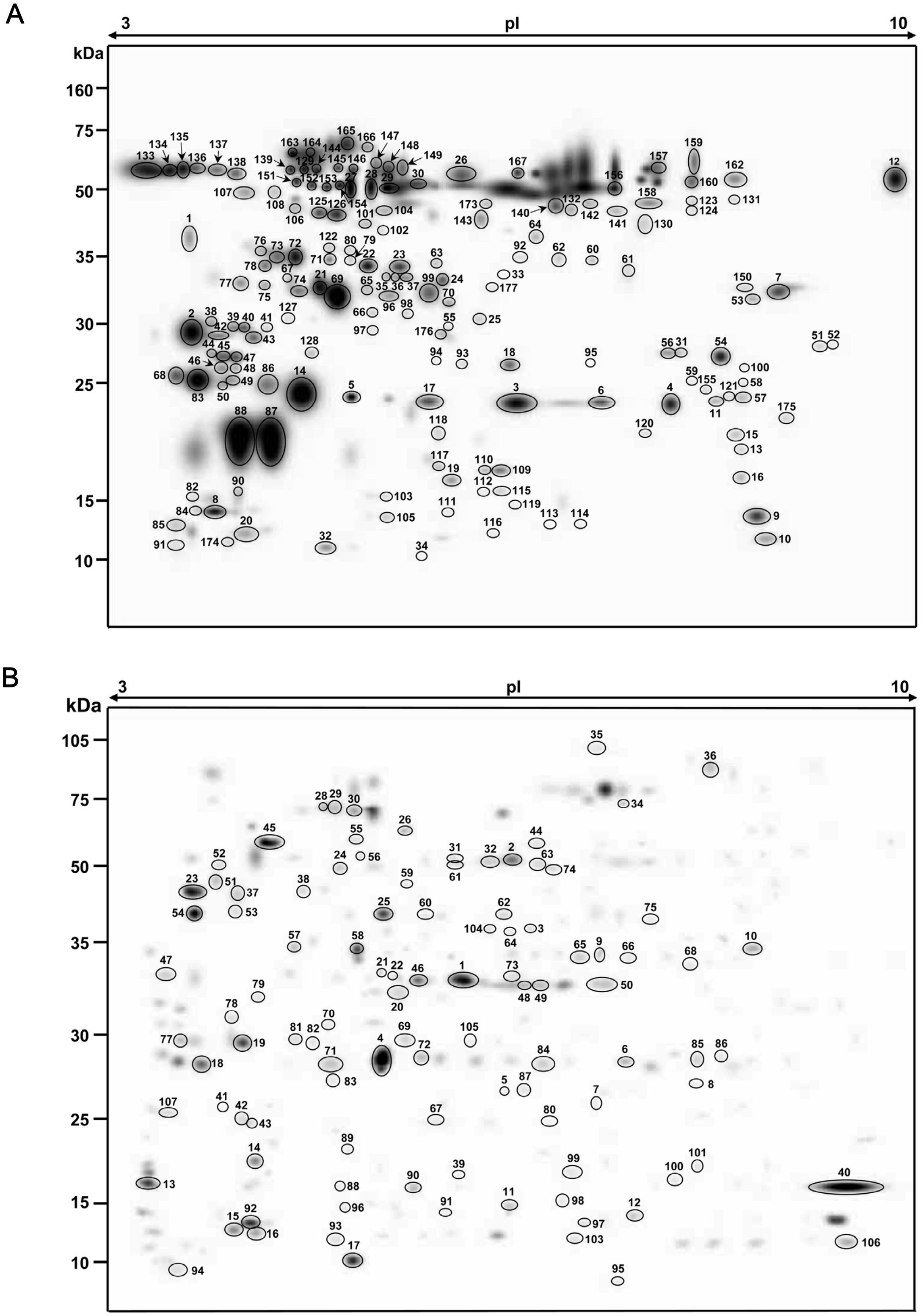
A
3
pI
10
kDa
160
75
50
35
30
25
15
10
B
3
pI
10
kDa
105
75
50
35
30
25
15
10
