# Supplementary material for: Apoplast proteome reveals that extracellular matrix contributes to multistress response in poplar
Source: BMC Genomics. 2010 Nov 29;11:674. doi: 10.1186/1471-2164-11-674 (PMC3091788; doi:10.1186/1471-2164-11-674)
Supplement: Additional file 2 — Supplementary Table S1. Proteins identified in poplar (P. deltoides) leaf apoplast using 2-D PAGE MS/MS. [file 1471-2164-11-674-S2.PDF]

Additional file 2

File format: PDF

Title: Supplementary Table S1

Description:
Table S1. Proteins identified in poplar (*P. deltoides*) leaf apoplast using 2-D PAGE MS/MS.

Poplar protein ID	Poplar transcript ID	Poplar new ID	Spot number	Mr/pI (theor.)	Number of matching peptides	Protein score	Protein identity/similarity	Glyco-protein**	Sequence similarity	Accession number	Organism	Signal peptide (SP)	Non-classical SP
**Cell wall metabolism**													
297914#*	gw1.88.36.1	POPTR_0017s08470.1	177	98.5/6.2	14	163	Beta-galactosidase		77%	Q5CCQ1	*Pyrus pyrifolia*	SA	yes
297914#*	gw1.88.36.1	POPTR_0017s08470.1	55	98.5/6.2	15	260	Beta-galactosidase		77%	Q5CCQ1	*Pyrus pyrifolia*	SA	yes
204338#*	gw1.IX.4803.1	POPTR_0009s01780.1	60	90.0/6.3	15	194	Beta-galactosidase		77%	Q93X57	*Fragaria ananassa*	yes	
204338#*	gw1.IX.4803.1	POPTR_0009s01780.1	62	90.0/6.3	15	238	Beta-galactosidase		77%	Q93X57	*Fragaria ananassa*	yes	
204338#*	gw1.IX.4803.1	POPTR_0009s01780.1	92	90.0/6.3	12	205	Beta-galactosidase		77%	Q93X57	*Fragaria ananassa*	yes	
204338#*	gw1.IX.4803.1	POPTR_0009s01780.1	11	90.0/6.3	8	143	Beta-galactosidase		77%	Q93X57	*Fragaria ananassa*	yes	
204338#*	gw1.IX.4803.1	POPTR_0009s01780.1	57	90.0/6.3	12	469	Beta-galactosidase		77%	Q93X57	*Fragaria ananassa*	yes	
209993#	gw1.V.5394.1	POPTR_0005s20280.1	57	85.3/9.0	4	106	Beta-galactosidase 16		63%	Q8GX69	*Arabidopsis thaliana*	no	yes
204338#*	gw1.IX.4803.1	POPTR_0009s01780.1	58	90.0/6.3	11	428	Beta-galactosidase		77%	Q93X57	*Fragaria ananassa*	yes	
209993#	gw1.V.5394.1	POPTR_0005s20280.1	58	85.3/9.0	4	106	Beta-galactosidase 16		63%	Q8GX69	*Arabidopsis thaliana*	no	yes
204338#*	gw1.IX.4803.1	POPTR_0009s01780.1	59	90.0/6.3	7	206	Beta-galactosidase		77%	Q93X57	*Fragaria ananassa*	yes	
209993#	gw1.V.5394.1	POPTR_0005s20280.1	59	85.3/9.0	7	80	Beta-galactosidase 16		63%	Q8GX69	*Arabidopsis thaliana*	no	yes
786149*	fgenesh4_pg.C_scaffold_164000005	POPTR_0018s14920.1	75	40.0/4.9	14	366	Alpha-galactosidase		83%	Q84VQ7	*Helianthus annuus*	no	yes
254860#	gw1.XVI.799.1	POPTR_0016s01920.1	102	36.9/6.1	7	66	Pectinesterase		78%	A2Q4U8	*Medicago truncatula*	no	yes
718566*	estExt_Genewise1_v1.C_LG_VII1401	POPTR_0007s04020.1	66	62.1/6.3	10	333	Pectin methylesterase-like protein		66%	Q9FXW9	*Arabidopsis thaliana*	yes	
718566*	estExt_Genewise1_v1.C_LG_VII1401	POPTR_0007s04020.1	176	62.1/6.3	7	226	Pectin methylesterase-like protein		66%	Q9FXW9	*Arabidopsis thaliana*	yes	
718566*	estExt_Genewise1_v1.C_LG_VII1401	POPTR_0007s04020.1	175	62.1/6.3	5	115	Pectin methylesterase-like protein		66%	Q9FXW9	*Arabidopsis thaliana*	yes	
718566*	estExt_Genewise1_v1.C_LG_VII1401	POPTR_0007s04020.1	173	62.1/6.3	9	113	Pectin methylesterase-like protein		66%	Q9FXW9	*Arabidopsis thaliana*	yes	
816882*	estExt_fgenesh4_pg.C_LG_II2363	POPTR_0002s23920.1	173	114.7/5.8	15	81	Alpha-mannosidase		71%	P94078	*Arabidopsis thaliana*	yes	
570444*	eugene3.00121097	POPTR_0012s13090.1	72	43.7/5.1	7	63	Pectinacetylesterase	box e	64%	Q9FF93	*Arabidopsis thaliana*	yes	
570444*	eugene3.00121097	POPTR_0012s13090.1	73	43.7/5.1	6	309	Pectinacetylesterase	box e	64%	Q9FF93	*Arabidopsis thaliana*	yes	
570444*	eugene3.00121097	POPTR_0012s13090.1	76	43.7/5.1	7	268	Pectinacetylesterase	box e	64%	Q9FF93	*Arabidopsis thaliana*	yes	
570444*	eugene3.00121097	POPTR_0012s13090.1	122	43.3/5.1	9	74	Pectinacetylesterase		64%	Q9FF93	*Arabidopsis thailana*	yes	
255102*	gw1.XVI.1041.1	POPTR_0016s02620.1	12	73.3/5.5	10	138	Alpha-L-arabinofuranosidase		74%	Q7X9G7	*Malus domestica*	yes	
255102*	gw1.XVI.1041.1	POPTR_0016s02620.1	27	73.3/5.5	10	146	Alpha-L-arabinofuranosidase	box a	74%	Q7X9G7	*Malus domestica*	yes	
255102*	gw1.XVI.1041.1	POPTR_0016s02620.1	28	73.3/5.5	10	146	Alpha-L-arabinofuranosidase	box a	74%	Q7X9G7	*Malus domestica*	yes	
255102*	gw1.XVI.1041.1	POPTR_0016s02620.1	29	73.3/5.5	9	161	Alpha-L-arabinofuranosidase	box a	74%	Q7X9G7	*Malus domestica*	yes	
255102*	gw1.XVI.1041.1	POPTR_0016s02620.1	30	73.3/5.5	10	138	Alpha-L-arabinofuranosidase	box a	74%	Q7X9G7	*Malus domestica*	yes	
207219#*	gw1.V.2620.1	POPTR_0005s07020.1	129	71.2/4.9	11	73	Beta-galactosidase	box a	72%	A5AXS9	*Vitis vinifera*	no	yes
255102*	gw1.XVI.1041.1	POPTR_0016s02620.1	129	73.3/5.5	12	84	Alpha-L-arabinofuranosidase	box a	74%	Q7X9G7	*Malus domestica*	yes	
255102*	gw1.XVI.1041.1	POPTR_0016s02620.1	133	73.3/5.5	12	84	Alpha-L-arabinofuranosidase	box a	74%	Q7X9G7	*Malus domestica*	yes	
255102*	gw1.XVI.1041.1	POPTR_0016s02620.1	134	73.3/5.5	9	149	Alpha-L-arabinofuranosidase	box a	74%	Q7X9G7	*Malus domestica*	yes	
255102*	gw1.XVI.1041.1	POPTR_0016s02620.1	135	73.3/5.5	9	102	Alpha-L-arabinofuranosidase	box a	74%	Q7X9G7	*Malus domestica*	yes	
255102*	gw1.XVI.1041.1	POPTR_0016s02620.1	136	73.3/5.5	10	280	Alpha-L-arabinofuranosidase	box a	74%	Q7X9G7	*Malus domestica*	yes	
255102*	gw1.XVI.1041.1	POPTR_0016s02620.1	137	73.3/5.5	12	239	Alpha-L-arabinofuranosidase	box a	74%	Q7X9G7	*Malus domestica*	yes	
255102*	gw1.XVI.1041.1	POPTR_0016s02620.1	138	73.3/5.5	11	194	Alpha-L-arabinofuranosidase	box a	74%	Q7X9G7	*Malus domestica*	yes	

Table S1. continued

207219[#,*]	gw1.V.2620.1	POPTR_0005s07020.1	139	71.2/4.9	8	91	Beta-galactosidase	box a	72%	A5AXS9	*Vitis vinifera*	no	yes
255102*	gw1.XVI.1041.1	POPTR_0016s02620.1	139	73.3/5.5	9	78	Alpha-L-arabinofuranosidase	box a	74%	Q7X9G7	*Malus domestica*	yes	
255102*	gw1.XVI.1041.1	POPTR_0016s02620.1	144	73.3/5.5	10	118	Alpha-L-arabinofuranosidase	box a	74%	Q7X9G7	*Malus domestica*	yes	
255102*	gw1.XVI.1041.1	POPTR_0016s02620.1	145	73.3/5.5	9	255	Alpha-L-arabinofuranosidase	box a	74%	Q7X9G7	*Malus domestica*	yes	
255102*	gw1.XVI.1041.1	POPTR_0016s02620.1	146	73.3/5.5	10	96	Alpha-L-arabinofuranosidase	box a	74%	Q7X9G7	*Malus domestica*	yes	
255102*	gw1.XVI.1041.1	POPTR_0016s02620.1	147	73.3/5.5	12	105	Alpha-L-arabinofuranosidase	box a	74%	Q7X9G7	*Malus domestica*	yes	
255102*	gw1.XVI.1041.1	POPTR_0016s02620.1	148	73.3/5.5	12	118	Alpha-L-arabinofuranosidase	box a	74%	Q7X9G7	*Malus domestica*	yes	
255102*	gw1.XVI.1041.1	POPTR_0016s02620.1	149	73.3/5.5	7	98	Alpha-L-arabinofuranosidase	box a	74%	Q7X9G7	*Malus domestica*	yes	
255102*	gw1.XVI.1041.1	POPTR_0016s02620.1	151	73.3/5.5	5	137	Alpha-L-arabinofuranosidase	box a	74%	Q7X9G7	*Malus domestica*	yes	
255102*	gw1.XVI.1041.1	POPTR_0016s02620.1	152	73.3/5.5	9	121	Alpha-L-arabinofuranosidase	box a	74%	Q7X9G7	*Malus domestica*	yes	
255102*	gw1.XVI.1041.1	POPTR_0016s02620.1	153	73.3/5.5	11	146	Alpha-L-arabinofuranosidase	box a	74%	Q7X9G7	*Malus domestica*	yes	
255102*	gw1.XVI.1041.1	POPTR_0016s02620.1	154	73.3/5.5	12	140	Alpha-L-arabinofuranosidase	box a	74%	Q7X9G7	*Malus domestica*	yes	
255102*	gw1.XVI.1041.1	POPTR_0016s02620.1	155	73.3/5.5	6	209	Alpha-L-arabinofuranosidase		74%	Q7X9G7	*Malus domestica*	yes	
255102*	gw1.XVI.1041.1	POPTR_0016s02620.1	156	73.3/5.5	10	154	Alpha-L-arabinofuranosidase	box a	74%	Q7X9G7	*Malus domestica*	yes	
255102*	gw1.XVI.1041.1	POPTR_0016s02620.1	157	73.3/5.5	11	63	Alpha-L-arabinofuranosidase	box a	74%	Q7X9G7	*Malus domestica*	yes	
255102*	gw1.XVI.1041.1	POPTR_0016s02620.1	158	73.3/5.5	12	130	Alpha-L-arabinofuranosidase	box a	74%	Q7X9G7	*Malus domestica*	yes	
255102*	gw1.XVI.1041.1	POPTR_0016s02620.1	159	73.3/5.5	11	79	Alpha-L-arabinofuranosidase	box a	74%	Q7X9G7	*Malus domestica*	yes	
255102*	gw1.XVI.1041.1	POPTR_0016s02620.1	160	73.3/5.5	11	101	Alpha-L-arabinofuranosidase	box a	74%	Q7X9G7	*Malus domestica*	yes	
255102*	gw1.XVI.1041.1	POPTR_0016s02620.1	162	73.3/5.5	9	68	Alpha-L-arabinofuranosidase	box a	74%	Q7X9G7	*Malus domestica*	yes	
816882*	estExt_fgenesh4_pg.C_LG_II2363	POPTR_0002s23920.1	21	114.8/5.8	15	212	Alpha-mannosidase	box h	71%	P94078	*Arabidopsis thaliana*	yes	
816882*	estExt_fgenesh4_pg.C_LG_II2363	POPTR_0002s23920.1	105	114.8/5.8	5	90	Alpha-mannosidase		71%	P94078	*Arabidopsis thaliana*	yes	
816882*	estExt_fgenesh4_pg.C_LG_II2363	POPTR_0002s23920.1	125	114.8/5.8	15	129	Alpha-mannosidase	box d	71%	P94078	*Arabidopsis thaliana*	yes	
816882*	estExt_fgenesh4_pg.C_LG_II2363	POPTR_0002s23920.1	126	114.8/5.8	17	110	Alpha-mannosidase	box d	71%	P94078	*Arabidopsis thaliana*	yes	
816882*	estExt_fgenesh4_pg.C_LG_II2363	POPTR_0002s23920.1	132	114.8/5.8	17	326	Alpha-mannosidase		71%	P94078	*Arabidopsis thaliana*	yes	
816882*	estExt_fgenesh4_pg.C_LG_II2363	POPTR_0002s23920.1	140	114.8/5.8	15	90	Alpha-mannosidase		71%	P94078	*Arabidopsis thaliana*	yes	
816882*	estExt_fgenesh4_pg.C_LG_II2363	POPTR_0002s23920.1	141	114.8/5.8	16	291	Alpha-mannosidase		71%	P94078	*Arabidopsis thaliana*	yes	
816882*	estExt_fgenesh4_pg.C_LG_II2363	POPTR_0002s23920.1	142	114.8/5.8	16	327	Alpha-mannosidase		71%	P94078	*Arabidopsis thaliana*	yes	
816882*	estExt_fgenesh4_pg.C_LG_II2363	POPTR_0002s23920.1	128	114.8/5.8	13	217	Alpha-mannosidase		71%	P94078	*Arabidopsis thaliana*	yes	
816882*	estExt_fgenesh4_pg.C_LG_II2363	POPTR_0002s23920.1	67	114.8/5.8	16	243	Alpha-mannosidase		71%	P94078	*Arabidopsis thaliana*	yes	
572859*	eugene3.00140904	POPTR_0014s14050.1	127	113.8/6.3	9	81	Alpha-mannosidase		70%	Q9FFX7	*Arabidopsis thaliana*	yes	
816882*	estExt_fgenesh4_pg.C_LG_II2363	POPTR_0002s23920.1	130	114.7/5.8	18	369	Alpha-mannosidase		71%	P94078	*Arabidopsis thaliana*	yes	
595225	eugene3.68730001	POPTR_0003s13160.1	107	38.7/4.9	4	136	Polygalacturonase-like protein	box b	75%	Q84LI7	*Fragaria ananassa*	no	yes
413310[#]	gw1.III.413.1	POPTR_0003s13160.1	108	47.1/4.7	5	157	Polygalacturonase-like protein	box b	74%	Q84LI7	*Fragaria ananassa*	no	yes
662785*	grail3.0016022201	POPTR_0013s02730.2	33	41.7/6.1	6	143	Lipolytic enzyme, G-D-S-L		75%	Q2HTP0	*Medicago truncatula*	yes	
662785*	grail3.0016022201	POPTR_0013s02730.2	35	41.7/6.1	11	266	Lipolytic enzyme, G-D-S-L		75%	Q2HTP0	*Medicago truncatula*	yes	
662785*	grail3.0016022201	POPTR_0013s02730.2	24	41.7/6.1	9	280	Lipolytic enzyme, G-D-S-L	box k	75%	Q2HTP0	*Medicago truncatula*	yes	
827727	estExt_fgenesh4_pg.C_1210040	POPTR_0006s18230.1	24	30.0/4.8	3	128	Proline-rich protein	box k	46%	Q94J26	*Oryza sativa subsp. japonica*	yes	
662785*	grail3.0016022201	POPTR_0013s02730.2	36	41.7/6.1	9	93	Lipolytic enzyme, G-D-S-L		75%	Q2HTP0	*Medicago truncatula*	yes	
827727	estExt_fgenesh4_pg.C_1210040	POPTR_0006s18230.1	36	30.0/4.8	4	92	Proline-rich protein		46%	Q94J26	*Oryza sativa subsp. japonica*	yes	
580490	eugene3.01210029	POPTR_0006s18240.1	36	42.3/5.1	7	75	GDSL-motif lipase/hydrolase-like protein		43%	Q9FK75	*Arabidopsis thaliana*	yes	

Table S1. continued

827727	estExt_fgenesh4_pg.C_1210040	POPTR_0006s18230.1	37	30.0/4.8	6	164	Proline-rich protein		46%	Q94J26	*Oryza sativa subsp. japonica*	yes	
580490	eugene3.01210029	POPTR_0006s18240.1	37	42.3/5.1	7	147	GDSL-motif lipase/hydrolase-like protein		43%	Q9FK75	*Arabidopsis thaliana*	yes	
830063*	estExt_fgenesh4_pm.C_LG_II0164	POPTR_0002s03580.1	98	33.9/5.5	19	465	Phenylcoumaran benzylic ether reductase		100%	O65904	*Populus trichocarpa*	no	no
648596	grail3.0045003902	POPTR_0004s16120.2	97	40.3/5.8	11	141	Glucan endo-1,3-beta-glucosidase 7		74%	Q9M069	*Arabidopsis thaliana*	yes	
209602[# *]	gw1.V.5003.1	POPTR_0005s16590.1	124	32.8/5.0	11	211	Glucan 1,3-beta-glucosidase		68%	Q8RU06	*Oryza sativa subsp. japonica*	no	yes
569295	eugene3.00111309	POPTR_0011s15750.1	116	102.5/7.1	5	65	Alpha-glucosidase 1		64%	Q9LYF8	*Arabidopsis thaliana*	SA	yes
570444*	eugene3.00121097	POPTR_0012s13090.1	80	43.7/5.1	6	145	Pectinacetylesterase		64%	Q9FF93	*Arabidopsis thaliana*	yes	
**Cell wall and stress related**													
547681*	eugene3.00010122	POPTR_0001s05050.1	1	36.8/4.5	5	140	Peroxidase	box c	98%	Q43101	*Populus trichocarpa*	yes	
817694*	estExt_fgenesh4_pg.C_LG_III1873	POPTR_0003s21660.1	65	33.4/5.8	8	146	Peroxidase N		67%	Q42517	*Armoracia rusticana*	no	yes
817694*	estExt_fgenesh4_pg.C_LG_III1873	POPTR_0003s21660.1	69	33.4/5.8	11	145	Peroxidase N	box i	67%	Q42517	*Armoracia rusticana*	no	yes
817694*	estExt_fgenesh4_pg.C_LG_III1873	POPTR_0003s21660.1	70	33.4/5.8	10	297	Peroxidase N	box m	67%	Q42517	*Armoracia rusticana*	no	yes
817694*	estExt_fgenesh4_pg.C_LG_III1873	POPTR_0003s21660.1	74	33.4/5.8	10	197	Peroxidase N	box i	67%	Q42517	*Armoracia rusticana*	no	yes
679511	grail3.0064002301	POPTR_0017s06550.2	7	32.0/8.7	5	75	Peroxidase a	box v	76%	Q66RM0	*Eucommia ulmoides*	yes	
679511	grail3.0064002301	POPTR_0017s06550.2	150	32.0/8.7	6	65	Peroxidase a	box v	76%	Q66RM0	*Eucommia ulmoides*	yes	
777213*	fgenesh4_pg.C_LG_XVI000455	POPTR_0016s05860.1	77	33.0/5.3	2	173	Peroxidase	box g	93%	Q08IT3	*Populus alba*	no	yes
825400*	estExt_fgenesh4_pg.C_LG_XVI1240	POPTR_0016s14030.1	54	33.4/8.1	9	72	Cationic peroxidase 1	box u	69%	P22195	*Arachis hypogaea*	yes	
800693	fgenesh4_pm.C_LG_IV000380	POPTR_0004s14240.1	25	34.6/5.2	8	201	Peroxidase ATP17a like protein		68%	Q67XK7	*Arabidopsis thaliana*	yes	
718485*	estExt_Genewise1_v1.C_LG_VII1054	POPTR_0007s02580.1	53	35.9/8.6	8	244	Peroxidase		74%	Q9ZNZ5	*Glycine max*	yes	
654740	grail3.0011011501	POPTR_0007s05100.1	96	36.5/5.3	15	288	Peroxidase 17		75%	Q9SJZ2	*Arabidopsis thaliana*	yes	
**Stress/defense**													
549955*	eugene3.00012396	POPTR_0001s30680.1	11	25.0/7.0	7	273	NtPRp27		72%	Q9XIY9	*Nicotiana tabacum*	yes	
549955*	eugene3.00012396	POPTR_0001s30680.1	3	25.0/7.0	6	289	NtPRp27	box p	72%	Q9XIY9	*Nicotiana tabacum*	yes	
549955*	eugene3.00012396	POPTR_0001s30680.1	4	25.0/7.0	6	193	NtPRp27	box p	72%	Q9XIY9	*Nicotiana tabacum*	yes	
549955*	eugene3.00012396	POPTR_0001s30680.1	6	25.0/7.0	10	217	NtPRp27	box p	72%	Q9XIY9	*Nicotiana tabacum*	yes	
549955*	eugene3.00012396	POPTR_0001s30680.1	121	25.0/7.0	5	295	NtPRp27		72%	Q9XIY9	*Nicotiana tabacum*	yes	
549955*	eugene3.00012396	POPTR_0001s30680.1	17	25.0/7.0	8	107	NtPRp27		72%	Q9XIY9	*Nicotiana tabacum*	yes	
201948[#]	gw1.IX.2413.1	POPTR_0009s09750.1	48	23.7/5.0	9	239	NtPRp27-like protein		66%	Q84XQ4	*Solanum tuberosum*	no	no
735049	estExt_Genewise1_v1.C_LG_XVI1825	POPTR_0016s05800.1	51	22.5/7.8	1	83	Beta-1,3 glucanase		90%	Q9M5I9	*Populus tremula x Populus alba*	no	yes
735049	estExt_Genewise1_v1.C_LG_XVI1825	POPTR_0016s05800.1	52	22.5/7.8	1	111	Beta-1,3 glucanase		90%	Q9M5I9	*Populus tremula x Populus alba*	no	yes
290846[#]	gw1.5405.1.1	POPTR_0001s26210.1	38	34.7/4.7	8	192	Beta-1,3-glucanase		69%	Q84RT6	*Fragaria ananassa*	no	yes
290846[#]	gw1.5405.1.1	POPTR_0001s26210.1	40	34.7/4.7	8	277	Beta-1,3-glucanase		69%	Q84RT6	*Fragaria ananassa*	no	yes
574380*	eugene3.00190854	POPTR_0019s12360.1	40	28.9/4.7	2	123	Class IV chitinase		70%	Q7X9F8	*Galega orientalis*	yes	
290846[#]	gw1.5405.1.1	POPTR_0001s26210.1	41	34.7/4.7	8	397	Beta-1,3-glucanase		69%	Q84RT6	*Fragaria ananassa*	no	yes
652688*	grail3.0024032801	POPTR_0006s04670.1	43	31.6/4.7	7	245	Beta-1,3-glucanase		80%	Q9M5I9	*Populus tremula x Populus alba*	yes	
259952	gw1.XVIII.493.1	POPTR_0018s14060.1	153	109.4/5.9	21	71	Salt-inducible protein	box a	52%	Q9LVD3	*Arabidopsis thailana*	no	yes
828660*	estExt_fgenesh4_pg.C_1970027	POPTR_0015s05980.1	56	31.7/8.4	7	199	Hevamine-A	box t	80%	P23472	*Hevea brasiliensis*	yes	

Table S1. continued

828660*	estExt_fgenesh4_pg.C_1970027	POPTR_0015s05980.1	31	31.7/8.4	6	166	Hevamine-A		80%	P23472	*Hevea brasiliensis*	yes	
233978[#]	gw1.XI.1958.1	POPTR_0012s01160.1	83	28.6/5.2	N-TS		Pathogenesis-related protein 8		75%	Q00MX4	*Malus domestica*	no	yes
746640*	estExt_Genewise1_v1.C_1970084	POPTR_0015s05990.1	83	30.8/4.4	N-TS		Acidic class III chitinase		72%	Q09Y38	*Citrullus lanatus*	yes	
669475*	grail3.0020019002	POPTR_0018s10490.1	87	24.7/7.8	N-TS		Thaumatin-like protein		76%	Q5ND92	*Actinidia deliciosa*	yes	
669475*	grail3.0020019002	POPTR_0018s10490.1	88	24.7/7.8	N-TS		Thaumatin-like protein		76%	Q5ND92	*Actinidia deliciosa*	yes	
574380*	eugene3.00190854	POPTR_0019s12360.1	50	28.9/4.7	2	123	Class IV chitinase		70%	Q7X9F8	*Galega orientalis*	yes	
270686[# *]	gw1.142.209.1	POPTR_0013s12870.1	2	27.1/4.4	5	184	Class IV chitinase		80%	Q9M2U5	*Arabidopsis thaliana*	no	yes
572334	eugene3.00140379	POPTR_0014s08860.1	100	29.9/6.9	9	112	Class III chitinase		54%	Q19AL0	*Panax ginseng*	yes	
586585	eugene3.01970027	POPTR_0015s06000.1	68	30.8/4.3	3	116	Acidic class III chitinase		69%	Q09Y38	*Citrullus lanatus*	yes	
586585	eugene3.01970027	POPTR_0015s06000.1	90	30.8/4.3	*3*	*88*	Acidic class III chitinase		69%	Q09Y38	*Citrullus lanatus*	yes	
811643*	fgenesh4_pm.C_scaffold_163000009	POPTR_0011s01280.1	103	21.6/6.4	5	114	Superoxide dismutase [Cu-Zn]		99%	A9PJW9	*Populus jackii*	no	yes
595511	eugene3.00700152	POPTR_0005s04590.1	111	15.3/5.6	6	70	Superoxide dismutase [Cu-Zn]		100%	A3FM77	*Populus trichocarpa*	no	yes
822230	estExt_fgenesh4_pg.C_LG_X1353	POPTR_0010s16050.1	34	13.0/5.7	4	71	*P.trichocarpa X P.deltoides* wound-responsive		98%	Q7DM48	*Populus trichocarpa*	no	no
819386*	estExt_fgenesh4_pg.C_LG_VI1270	POPTR_0006s19310.1	10	13.8/8.9	6	77	Blight-associated protein p12		49%	Q6K4C4	*Oryza sativa subsp. japonica*	yes	
825296*	estExt_fgenesh4_pg.C_LG_XVI0953	POPTR_0016s10140.1	9	11.9/8.1	7	62	Protease inhibitor/seed storage/lipid transfer protein (LTP) family protein		74%	A9XNQ1	*Sonneratia caseolaris*	yes	
819822	estExt_fgenesh4_pg.C_LG_VII0502	POPTR_0007s05650.1	84	18.6/4.7	3	377	Dehydration stress-induced protein		68%	Q9AXN5	*Brassica napus*	yes	
832078	estExt_fgenesh4_pm.C_LG_VI0650	POPTR_0006s24030.1	119	17.5/6.2	3	99	Cytosolic class II low molecular weight heat shock protein		88%	Q9XGS6	*Prunus dulcis*	no	no
723969	estExt_Genewise1_v1.C_LG_X0701	POPTR_0010s16030.1	174	12.4/4.9	6	182	*Populus x generosa* pop3 peptide		100%	Q41049	*Populus jackii*	no	yes
272681[# *]	gw1.152.150.1	POPTR_0006s06100.2	101	56.8/6.0	14	553	Heparanase-like protein 3		66%	Q9FZP1	*Arabidopsis thaliana*	no	yes
201272	gw1.IX.1737.1	POPTR_0009s12090.1	98	34.1/7.8	3	90	Isoflavone reductase related protein		80%	O81355	*Pyrus communis*	no	yes

Proteolysis

835003*	estExt_fgenesh4_pm.C_LG_XIV0520	POPTR_0014s17580.1	49	55.6/5.4	7	130	Serine carboxypeptidase-like 20		72%	Q8L7B2	*Arabidopsis thaliana*	yes	
560367*	eugene3.00060326	POPTR_0006s03490.1	39	51.1/4.9	6	240	Serine carboxypeptidase family protein		65%	Q2R3G8	*Oryza sativa subsp. japonica*	yes	
199556[# *]	gw1.IX.21.1	POPTR_0009s00820.1	42	49.0/5.4	7	87	Serine carboxypeptidase-like 51		67%	Q67Y83	*Arabidopsis thaliana*	no	yes
560367*	eugene3.00060326	POPTR_0006s03490.1	19	51.1/4.9	6	478	Serine carboxypeptidase family protein		65%	Q2R3G8	*Oryza sativa subsp. japonica*	yes	
797202	fgenesh4_pm.C_LG_I000205	POPTR_0001s13140.1	19	50.9/5.6	3	101	Serine carboxypeptidase-like 45		79%	Q93Y09	*Arabidopsis thaliana*	yes	
835003*	estExt_fgenesh4_pm.C_LG_XIV0520	POPTR_0014s17580.1	117	55.6/5.4	4	152	Serine carboxypeptidase-like 20	box r	72%	Q8L7B2	*Arabidopsis thaliana*	yes	
835003*	estExt_fgenesh4_pm.C_LG_XIV0520	POPTR_0014s17580.1	115	55.6/5.4	6	202	Serine carboxypeptidase-like 20		72%	Q8L7B2	*Arabidopsis thaliana*	yes	
835003*	estExt_fgenesh4_pm.C_LG_XIV0520	POPTR_0014s17580.1	45	55.6/5.4	7	305	Serine carboxypeptidase-like 20		72%	Q8L7B2	*Arabidopsis thaliana*	yes	
835003*	estExt_fgenesh4_pm.C_LG_XIV0520	POPTR_0014s17580.1	46	55.6/5.4	8	239	Serine carboxypeptidase-like 20		72%	Q8L7B2	*Arabidopsis thaliana*	yes	
199556[# *]	gw1.IX.21.1	POPTR_0009s00820.1	44	49.0/5.4	3	98	Serine carboxypeptidase-like 51		67%	Q67Y83	*Arabidopsis thaliana*	no	yes
249095[# *]	gw1.XIX.1495.1	POPTR_0019s08160.1	109	52.4/5.2	10	159	Wound-inducible carboxypeptidase	box s	64%	Q9M513	*Solanum lycopersicum*	no	yes
263224[#]	gw1.10474.1.1	POPTR_0019s08160.1	109	9.1/5.1	4	104	Serine carboxypeptidase-like 20	box s	83%	Q8L7B2	*Arabidopsis thaliana*	no	no
249095[# *]	gw1.XIX.1495.1	POPTR_0019s08160.1	110	52.4/5.2	5	170	Wound-inducible carboxypeptidase	box s	64%	Q9M513	*Solanum lycopersicum*	no	yes
263224[#]	gw1.10474.1.1	POPTR_0019s08160.1	110	9.1/5.1	3	140	Serine carboxypeptidase-like 20	box s	83%	Q8L7B2	*Arabidopsis thaliana*	no	no
249095[# *]	gw1.XIX.1495.1	POPTR_0019s08160.1	112	52.4/5.2	6	92	Wound-inducible carboxypeptidase		64%	Q9M513	*Solanum lycopersicum*	no	yes
825802*	estExt_fgenesh4_pg.C_LG_XVIII0531	POPTR_0018s03020.1	76	49.9/7.4	6	89	41 kD chloroplast nucleoid DNA binding protein		45%	Q8H9F4	*Nicotiana sylvestris*	yes	

Table S1. continued

825802*	estExt_fgenesh4_pg.C_LG_XVIII0531	POPTR_0018s03020.1	18	49.9/7.4	6	205	41 kD chloroplast nucleoid DNA binding protein	box o	45%	Q8H9F4	*Nicotiana sylvestris*	yes	
825802*	estExt_fgenesh4_pg.C_LG_XVIII0531	POPTR_0018s03020.1	94	49.9/7.4	4	162	41 kD chloroplast nucleoid DNA binding protein		45%	Q8H9F4	*Nicotiana sylvestris*	yes	
825802*	estExt_fgenesh4_pg.C_LG_XVIII0531	POPTR_0018s03020.1	78	49.9/7.4	6	276	41 kD chloroplast nucleoid DNA binding protein	box f	45%	Q8H9F4	*Nicotiana sylvestris*	yes	
825802*	estExt_fgenesh4_pg.C_LG_XVIII0531	POPTR_0018s03020.1	95	49.9/7.4	3	140	41 kD chloroplast nucleoid DNA binding protein		45%	Q8H9F4	*Nicotiana sylvestris*	yes	
560367*	eugene3.00060326	POPTR_0006s03490.1	43	51.1/4.9	5	135	Serine carboxypeptidase family protein		65%	Q2R3G8	*Oryza sativa subsp. japonica*	yes	
709916*	estExt_Genewise1_v1.C_LG_II0461	POPTR_0002s02010.1	26	77.2/5.3	4	81	Subtilisin-like serine protease	box a	61%	Q8LAE1	*Arabidopsis thailana*	no	yes
709916*	estExt_Genewise1_v1.C_LG_II0461	POPTR_0002s02010.1	64	77.2/5.3	8	309	Subtilisin-like serine protease	box n	61%	Q8LAE1	*Arabidopsis thailana*	no	yes
709916*	estExt_Genewise1_v1.C_LG_II0461	POPTR_0002s02010.1	165	77.2/5.3	9	78	Subtilisin-like serine protease	box a	61%	Q8LAE1	*Arabidopsis thailana*	no	yes
709916*	estExt_Genewise1_v1.C_LG_II0461	POPTR_0002s02010.1	166	77.2/5.3	9	70	Subtilisin-like serine protease	box a	61%	Q8LAE1	*Arabidopsis thailana*	no	yes
551801*	eugene3.00021116	POPTR_0002s12130.1	167	82.0/6.6	16	301	Serine protease	box a	98%	Q8RVJ7	*Populus canadensis*	yes	
781559*	fgenesh4_pg.C_scaffold_40000309	POPTR_0014s02650.1	164	83.4/5.3	8	163	Subtilase	box a	50%	Q84TR6	*Casuarina glauca*	yes	
781559*	fgenesh4_pg.C_scaffold_40000309	POPTR_0014s02650.1	163	83.4/5.3	11	102	Subtilase	box a	50%	Q84TR6	*Casuarina glauca*	yes	
781583*	fgenesh4_pg.C_scaffold_40000333	POPTR_0014s02410.1	113	50.4/5.4	9	316	Cysteine protease CP1		82%	Q52QX8	*Manihot esculenta*	yes	
675847*	grail3.0028002001	POPTR_0006s14400.1	47	39.3/6.3	7	252	Cysteine protease CP1		79%	A5HIJ1	*Actinidia deliciosa*	yes	
417502#	gw1.VI.1875.1	POPTR_0006s24850.1	99	49.9/5.2	7	134	Nucleoid DNA-binding protein cnd41-like protein	box l	55%	Q9LEW2	*Arabidopsis thaliana*	yes	

Carbohydrate metabolism

644125*	grail3.0033012902	POPTR_0515s00220.2	63	44.1/5.1	11	302	Alpha-amylase		78%	Q7X9T1	*Phaseolus angularis*	no	yes
644125*	grail3.0033012902	POPTR_0515s00220.2	22	44.1/5.1	11	302	Alpha-amylase		78%	Q7X9T1	*Phaseolus angularis*	no	yes
644125*	grail3.0033012902	POPTR_0515s00220.2	23	44.1/5.1	14	325	Alpha-amylase		78%	Q7X9T1	*Phaseolus angularis*	no	yes
644125*	grail3.0033012902	POPTR_0515s00220.2	71	44.1/5.1	12	277	Alpha-amylase		78%	Q7X9T1	*Phaseolus angularis*	no	yes
644125*	grail3.0033012902	POPTR_0515s00220.2	79	44.1/5.1	8	131	Alpha-amylase		78%	Q7X9T1	*Phaseolus angularis*	no	yes
575698*	eugene3.00151093	POPTR_0015s14380.1	143	47.9/5.7	13	113	Enolase		100%	A9PD49	*Populus trichocarpa*	no	yes
772214*	fgenesh4_pg.C_LG_XI001367	POPTR_0011s16170.1	130	59.6/6.1	15	206	Carbohydrate oxidase		59%	Q8SA59	*Helianthus annuus*	yes	
772214*	fgenesh4_pg.C_LG_XI001367	POPTR_0011s16170.1	131	59.6/6.1	13	116	Carbohydrate oxidase		59%	Q8SA59	*Helianthus annuus*	yes	
772214*	fgenesh4_pg.C_LG_XI001367	POPTR_0011s16170.1	123	59.6/6.1	16	247	Carbohydrate oxidase		59%	Q8SA59	*Helianthus annuus*	yes	
772214*	fgenesh4_pg.C_LG_XI001367	POPTR_0011s16170.1	124	59.6/6.1	12	74	Carbohydrate oxidase		59%	Q8SA59	*Helianthus annuus*	yes	
772214*	fgenesh4_pg.C_LG_XI001367	POPTR_0011s16170.1	104	59.6/6.1	13	173	Carbohydrate oxidase		59%	Q8SA59	*Helianthus annuus*	yes	
772214*	fgenesh4_pg.C_LG_XI001367	POPTR_0011s16170.1	106	59.6/6.1	15	412	Carbohydrate oxidase		59%	Q8SA59	*Helianthus annuus*	yes	
287193#	gw1.40.953.1	POPTR_0014s02270.1	93	25.7/5.6	6	83	Carbohydrate esterase		57%	Q8L9J9	*Arabidopsis thaliana*	no	yes

Other metabolic processes

553231	eugene3.00002546	POPTR_0002s25070.1	59	27.1/8.6	5	168	Auxin-induced protein 12		41%	Q94BT2	*Arabidopsis thaliana*	yes	
816232*	estExt_fgenesh4_pg.C_LG_II0662	POPTR_0002s07290.1	61	41.1/6.3	11	118	Alcohol dehydrogenase 2		87%	Q9FZ01	*Vitis vinifera*	no	no
782655	fgenesh4_pg.C_scaffold_70000003	POPTR_0005s06140.1	61	41.1/6.1	7	84	Alcohol dehydrogenase		92%	Q19P40	*Dimocarpus longan*	no	no
410110#	gw1.II.1445.1	POPTR_0002s13970.1	114	18.1/5.7	7	328	Nucleoside diphosphate kinase		99%	A9PGV1	*Populus trichocarpa*	no	no
666348	grail3.0005000301	POPTR_0015s07660.1	130	28.4/6.6	10	67	(-)-isopiperitenol dehydrogenase		69%	A7XDE9	*Mentha haplocalyx var. piperascens*	no	no
652151*	grail3.0002074001	POPTR_0005s26740.1	45	17.0/5.1	4	85	Plastocyanin A		100%	P00299	*Populus nigra*	no	yes
286504#	gw1.40.264.1	POPTR_0014s04850.1	20	16.5/5.0	1	102	Cu2+ plastocyanin-like		43%	Q6V5E4	*Olimarabidopsis pumila*	yes	

Table S1. continued

816369	estExt_fgenesh4_pg.C_LG_II0927	POPTR_0002s10150.1	32	18.8/5.6	2	226	Blue copper-like protein		55%	A3F8V0	*Gossypium hirsutum*	yes	
816369	estExt_fgenesh4_pg.C_LG_II0927	POPTR_0002s10150.1	91	18.8/5.6	1	81	Blue copper-like protein		55%	A3F8V0	*Gossypium hirsutum*	yes	
816369	estExt_fgenesh4_pg.C_LG_II0927	POPTR_0002s10150.1	174	18.8/5.6	1	89	Blue copper-like protein		55%	A3F8V0	*Gossypium hirsutum*	yes	
417410[#]	gw1.VI.1783.1	POPTR_0006s06650.1	85	31.5/5.2	1	96	Dicyanin		44%	Q9M510	*Solanum lycopersicum*	yes	
569314	eugene3.00111328	POPTR_0011s15950.1	104	58.7/9.3	5	65	FAD linked oxidase, N-terminal		59%	Q2HTY4	*Medicago truncatula*	yes	
569314	eugene3.00111328	POPTR_0011s15950.1	106	58.7/9.3	4	74	FAD linked oxidase, N-terminal		59%	Q2HTY4	*Medicago truncatula*	yes	
**Unclassified**													
718495	estExt_Genewise1_v1.C_LG_VII1106	POPTR_1698s00200.1	17	26.4/5.8	14	199	Cysteine-rich repeat secretory protein 38		58%	Q9LRJ9	*Arabidopsis thaliana*	yes	
718495	estExt_Genewise1_v1.C_LG_VII1106	POPTR_1698s00200.1	5	26.4/5.8	9	135	Cysteine-rich repeat secretory protein 38	box j	58%	Q9LRJ9	*Arabidopsis thaliana*	yes	
718495	estExt_Genewise1_v1.C_LG_VII1106	POPTR_1698s00200.1	14	26.4/5.8	11	288	Cysteine-rich repeat secretory protein 38	box j	58%	Q9LRJ9	*Arabidopsis thaliana*	yes	
718495	estExt_Genewise1_v1.C_LG_VII1106	POPTR_1698s00200.1	86	26.4/5.8	14	213	Cysteine-rich repeat secretory protein 38	box j	58%	Q9LRJ9	*Arabidopsis thaliana*	yes	
562320	eugene3.00070258	POPTR_1698s00200.1	118	26.4/5.8	7	147	Cysteine-rich repeat secretory protein 38		58%	Q9LRJ9	*Arabidopsis thaliana*	yes	
562320	eugene3.00070258	POPTR_1698s00200.1	122	26.4/5.8	9	267	Cysteine-rich repeat secretory protein 38		58%	Q9LRJ9	*Arabidopsis thaliana*	yes	
718495	estExt_Genewise1_v1.C_LG_VII1106	POPTR_1698s00200.1	49	26.4/5.8	5	94	Cysteine-rich repeat secretory protein 38		58%	Q9LRJ9	*Arabidopsis thaliana*	yes	
824970	estExt_fgenesh4_pg.C_LG_XVI0047	POPTR_0016s01210.1	120	41.9/9.6	4	87	Membrane protein		57%	Q9AR56	*Solanum lycopersicum*	no	no
640081[# *]	grail3.0008013701	POPTR_0001s13320.1	15	17.4/9.6	5	148	Leucine-rich repeat protein		67%	A5HKK5	*Nicotiana tabacum*	no	yes
549865*	eugene3.00012306	POPTR_0001s31740.1	13	20.9/8.7	3	274	Tumor-related protein		37%	P93378	*Nicotiana tabacum*	yes	
549865*	eugene3.00012306	POPTR_0001s31740.1	16	20.9/8.7	5	164	Tumor-related protein		37%	P93378	*Nicotiana tabacum*	yes	
814847*	estExt_fgenesh4_pg.C_LG_I0347	POPTR_0001s05560.1	162	95.2/5.5	17	102	no sequence similarity to a known protein	box a				yes	
814847*	estExt_fgenesh4_pg.C_LG_I0347	POPTR_0001s05560.1	8	95.2/5.5	10	70	no sequence similarity to a known protein					yes	
814847*	estExt_fgenesh4_pg.C_LG_I0347	POPTR_0001s05560.1	82	95.2/5.5	9	529	no sequence similarity to a known protein					yes	

[#] protein sequence in JGI database is incomplete

* protein was also identified via 2D-LC MSMS

** based on staining with specific glyco dye protein appears to be glycosylated. Boxes correspond to glycoprotein on Figure S2

N-TS - protein was identified via N-terminal sequencing

SA - protein is predicted to serve as signal anchor
