# Supplementary material for: Apoplast proteome reveals that extracellular matrix contributes to multistress response in poplar
Source: BMC Genomics. 2010 Nov 29;11:674. doi: 10.1186/1471-2164-11-674 (PMC3091788; doi:10.1186/1471-2164-11-674)
Supplement: Additional file 3 — Supplementary Table S2. Proteins identified in poplar (P. deltoides) leaf apoplast using 2-D LC MS/MS. [file 1471-2164-11-674-S3.PDF]

Additional file 3

File format: PDF

Title: Supplementary Table S2

Description:
Table S2. Proteins identified in poplar (*P. deltoides*) leaf apoplast using 2-D LC MS/MS.

Poplar Protein ID	Poplar transcript ID	Poplar new ID	Mr/pI (theor.)	Number of matched peptides total/unique	Protein score	Protein identity/similarity	Sequence similarity	Accession number	Organism	Signal peptide (SP)	Non-classical SP
**Cell wall metabolism**											
422858[#]	gw1.XII.1318.1	POPTR_0012s10830.1	112.5/5.7	3/2	28.19	Alpha-mannosidase	70%	Q8LPJ3	*Arabidopsis thaliana*	yes	
816882*	estExt_fgenesh4_pg.C_LG_II2363	POPTR_0002s23920.1	114.8/5.8	23/21	198.32	Alpha-mannosidase	71%	P94078	*Arabidopsis thaliana*	yes	
757134	fgenesh4_pg.C_LG_III000676	POPTR_0007s09730.1	114.0/5.5	2/2	14.24	Alpha-mannosidase	67%	Q9FKW9	*Arabidopsis thaliana*	SA	yes
572859*	eugene3.00140904	POPTR_0014s14050.1	113.8/6.3	4/3	26.19	Alpha-mannosidase	70%	Q9FFX7	*Arabidopsis thaliana*	yes	
786149*	fgenesh4_pg.C_scaffold_164000005	POPTR_0018s14920.1	40.0/4.9	4/3	30.20	Alpha-galactosidase	83%	Q84VQ7	*Helianthus annuus*	no	yes
204338[#*]	gw1.IX.4803.1	POPTR_0009s01780.1	90.0/6.3	9/8	90.26	Beta-galactosidase	77%	Q93X57	*Fragaria ananassa*	yes	
297914[#*]	gw1.88.36.1	POPTR_0017s08470.1	98.5/6.2	3/3	30.21	Beta-galactosidase	77%	Q5CCQ1	*Pyrus pyrifolia*	SA	yes
207219[#*]	gw1.V.2620.1	POPTR_0005s07020.1	71.2/4.9	12/9	78.33	Beta-galactosidase	72%	A5AXS9	*Vitis vinifera*	no	yes
798675	fgenesh4_pm.C_LG_II000364	POPTR_0002s08120.1	89.6/9.4	2/2	18.17	Beta-galactosidase 16	66%	Q8GX69	*Arabidopsis thaliana*	no	yes
228333[#]	gw1.X.3030.1	POPTR_0010s26170.1	41.1/6.8	2/2	18.12	Acid alpha galactosidase 1	83%	Q2MK92	*Cucumis sativus*	no	yes
209602[#*]	gw1.V.5003.1	POPTR_0005s16590.1	32.8/5.0	7/7	40.24	Glucan 1,3-beta-glucosidase	68%	Q8RU06	*Oryza sativa subsp. japonica*	no	yes
171426[#]	gw1.I.26.1	POPTR_0001s21430.1	79.5/6.0	11/2	70.25	Xylan 1,4-beta-xylosidase	93%	Q2MCJ5	*Populus tremula x Populus alba*	no	yes
799561	fgenesh4_pm.C_LG_III000026	POPTR_0003s01730.1	83.4/5.7	10/1	78.25	Xylan 1,4-beta-xylosidase	96%	Q2MCJ6	*Populus tremula x Populus alba*	yes	
255102*	gw1.XVI.1041.1	POPTR_0016s02620.2	73.3/5.5	30/21	240.37	Alpha-L-arabinofuranosidase	74%	Q7X9G7	*Malus domestica*	yes	
204508[#]	gw1.IX.4973.1	POPTR_0009s01240.1	51.5/4.9	3/3	20.23	Alpha-L-fucosidase 1	73%	Q8GW72	*Arabidopsis thaliana*	no	yes
830063*	estExt_fgenesh4_pm.C_LG_II0164	POPTR_0002s03580.1	33.9/5.5	3/1	30.18	Phenylcoumaran benzylic ether reductase	100%	O65904	*Populus trichocarpa*	no	no
718566*	estExt_Genewise1_v1.C_LG_VII1401	POPTR_0007s04020.1	62.1/6.3	6/3	50.27	Pectin methylesterase-like protein	66%	Q9FXW9	*Arabidopsis thaliana*	yes	
253960[#]	gw1.XV.3396.1	POPTR_0015s12990.1	39.9/8.3	2/1	20.26	Pectinacetylesterase	68%	Q9FF93	*Arabidopsis thaliana*	no	no
827492	estExt_fgenesh4_pg.C_860139	POPTR_0004s24220.4	44.2/9.1	1/1	10.14	Pectinacetylesterase	64%	Q41695	*Phaseolus aureus*	yes	
570444*	eugene3.00121097	POPTR_0012s13090.1	43.3/5.1	7/6	60.30	Pectinacetylesterase	64%	Q9FF93	*Arabidopsis thailana*	yes	
755730	fgenesh4_pg.C_LG_II001910	POPTR_0002s18800.1	50.7/4.9	4/3	38.19	Polygalacturonase	68%	Q75LI0	*Oryza sativa subsp. japonica*	yes	
644497	grail3.0039009101	POPTR_0002s16390.1	51.5/5.9	5/5	40.30	Polygalacturonase-like protein	80%	Q84LI7	*Fragaria ananassa*	yes	
662785*	grail3.0016022201	POPTR_0013s02730.2	41.7/6.1	7/3	50.31	Lipolytic enzyme, G-D-S-L	75%	Q2HTP0	*Medicago truncatula*	yes	
225922[#]	gw1.X.619.1	POPTR_0010s15510.1	61.8/8.9	1/1	6.13	O-linked GlcNAc transferase	69%	Q9LSP3	*Arabidopsis thaliana*	no	no
551701	eugene3.00021016	POPTR_0002s11090.1	72.9/4.8	6/5	40.18	LG27/30-like gene (rhamnogalacturonate lyase)	67%	Q9STV1	*Arabidopsis thaliana*	no	yes
836412	estExt_fgenesh4_pm.C_400050	POPTR_0014s04360.1	20.2/8.0	1/1	10.14	Sts15 protein	59%	P93569	*Solanum tuberosum*	yes	
272681[#*]	gw1.152.150.1	POPTR_0006s06100.1	56.8/6.0	4/4	40.33	Heparanase-like protein 3	66%	Q9FZP1	*Arabidopsis thaliana*	no	yes
262951[#]	gw1.XVIII.3492.1	POPTR_0025s00410.1	55.9/8.0	2/2	16.33	Heparanase-like protein 3	68%	Q9FZP1	*Arabidopsis thaliana*	no	yes
**Cell wall and stress related**											
774930	fgenesh4_pg.C_LG_XIV000840	POPTR_0014s14000.1	33.5/9.1	1/1	18.19	Peroxidase	98%	Q58GF4	*Populus alba x Populus tremula var. glandulosa*	yes	
825400*	estExt_fgenesh4_pg.C_LG_XVI1240	POPTR_0016s14030.1	33.4/8.1	5/4	40.29	Cationic peroxidase 1	69%	P22195	*Arachis hypogaea*	yes	
817694*	estExt_fgenesh4_pg.C_LG_III1873	POPTR_0003s21660.1	33.4/5.8	12/10	106.30	Peroxidase N	67%	Q42517	*Armoracia rusticana*	no	yes

Table S2. continued

718485*	estExt_Genewise1_v1.C_LG_VII1054	POPTR_0007s02580.1	35.9/8.6	6/4	60.22	Peroxidase	74%	Q9ZNZ5	*Glycine max*	yes	
777213*	fgenesh4_pg.C_LG_XVI000455	POPTR_0149s00200.1	33.0/5.3	4/3	40.30	Peroxidase	93%	Q08IT3	*Populus alba*	no	yes
208491[#]	gw1.V.3892.1	POPTR_0005s14190.1	32.6/5.2	3/2	28.27	Peroxidase 10	66%	Q9FX85	*Arabidopsis thaliana*	no	yes
547681*	eugene3.00010122	POPTR_0001s05050.1	36.8/4.5	4/1	38.25	Peroxidase	98%	Q43101	*Populus trichocarpa*	yes	
**Stress/defense**											
249950[#]	gw1.XIX.2350.1	POPTR_0019s12350.1	27.9/4.5	2/1	18.13	Class IV chitinase	75%	Q9M2U5	*Arabidopsis*	yes	
270686[#*]	gw1.142.209.1	POPTR_0013s12870.1	27.1/4.4	7/7	60.41	Class IV chitinase	80%	Q9M2U5	*Arabidopsis thaliana*	no	yes
574380*	eugene3.00190854	POPTR_0019s12360.1	28.9/4.7	4/1	30.29	Class IV chitinase	70%	Q7X9F8	*Galega orientalis*	yes	
746640*	estExt_Genewise1_v1.C_1970084	POPTR_0015s05990.1	30.8/4.4	6/3	60.48	Acidic class III chitinase	72%	Q09Y38	*Citrullus lanatus*	yes	
828660*	estExt_fgenesh4_pg.C_1970027	POPTR_0015s05980.1	31.7/8.4	8/7	60.25	Hevamine-A	80%	P23472	*Hevea brasiliensis*	yes	
203165[#]	gw1.IX.3630.1	POPTR_0001s26210.1	33.7/5.4	8/2	20.30	Beta-1,3-glucanase	67%	Q56AP0	*Fragaria ananassa*	no	yes
652688*	grail3.0024032801	POPTR_0006s04670.1	31.6/4.7	4/4	30.16	Beta-1,3 glucanase	80%	Q9M5I9	*Populus tremula x Populus alba*	yes	
669475*	grail3.0020019002	POPTR_0018s10490.1	24.7/7.8	5/4	100.30	Thaumatin-like protein	76%	Q5ND92	*Actinidia deliciosa*	yes	
747341	estExt_Genewise1_v1.C_4720001	POPTR_0001s09000.1	24.1/5.6	4/1	40.26	Thaumatin-like protein isoform 2	71%	Q2VAC9	*Ficus awkeotsang*	yes	
649635	grail3.0001063901	POPTR_0009s09760.1	25.1/5.0	4/1	38.15	NtPRp27	73%	Q9XIY9	*Nicotiana tabacum*	yes	
549955*	eugene3.00012396	POPTR_0001s30680.1	25.0/7.0	10/5	100.37	NtPRp27	72%	Q9XIY9	*Nicotiana tabacum*	yes	
669494	grail3.0020020701	POPTR_0018s10730.1	13.6/4.7	2/2	20.32	Blight-associated protein p12	60%	Q653F1	*Oryza sativa subsp. japonica*	yes	
781921	fgenesh4_pg.C_scaffold_44000051	POPTR_0003s02750.1	20.1/9.3	1/1	8.15	Pathogenesis-related transcriptional factor	70%	Q2HUA6	*Medicago truncatula*	no	no
579227	eugene3.10400001	POPTR_0185s00200.1	34.9/5.4	1/1	8.13	Resistance protein RGC2	25%	Q6Y136	*Lactuca sativa*	no	yes
198468	gw1.IV.3557.1	POPTR_0004s08710.1	58.2/6.5	1/1	8.17	Disease resistance protein	49%	Q9C784	*Arabidopsis thaliana*	no	yes
819386*	estExt_fgenesh4_pg.C_LG_VI1270	POPTR_0006s19310.1	13.8/8.9	4/4	40.20	Blight-associated protein p12	49%	Q6K4C4	*Oryza sativa subsp. japonica*	yes	
825296*	estExt_fgenesh4_pg.C_LG_XVI0953	POPTR_0016s10140.1	11.9/8.1	6/6	60.30	Protease inhibitor/seed storage/lipid transfer protein (LTP) family protein	74%	A9XNQ1	*Sonneratia caseolaris*	yes	
648132	grail3.0038010001	POPTR_0004s08500.1	11.8/9.2	1/1	10.24	Non-specific lipid-transfer protein	91%	A9PJG2	*Populus jackii*	yes	
761882	fgenesh4_pg.C_LG_VI000192	POPTR_0006s02260.1	23.5/5.4	2/2	20.18	Abrin-a	33%	P11140	*Abrus precatorius*	SA	yes
294386[#]	gw1.6840.1.1	POPTR_0001s09570.1	7.3/7.7	1/1	10.20	Osmotin-like protein linusitin	92%	Q8GUQ2	*Linum usitatissimum*	no	yes
811643*	fgenesh4_pm.C_scaffold_163000009	POPTR_0011s01280.1	21.6/6.4	3/2	30.23	Superoxide dismutase [Cu-Zn]	99%	A9PJW9	*Populus jackii*	no	yes
**Proteolysis**											
740448	estExt_Genewise1_v1.C_400832	POPTR_0014s02630.1	79.5/5.1	9/9	60.22	Subtilase	51%	Q84TR6	*Casuarina glauca*	no	yes
781559*	fgenesh4_pg.C_scaffold_40000309	POPTR_0014s02650.1	83.4/5.3	8/8	70.27	Subtilase	50%	Q84TR6	*Casuarina glauca*	yes	
208844	gw1.V.4245.1	POPTR_0005s18880.1	80.4/6.6	1/1	10.17	Subtilisin-like protease	77%	9XG40	*Nicotiana tabacum*	yes	
709916*	estExt_Genewise1_v1.C_LG_II0461	POPTR_0002s02010.1	77.2/5.3	16/16	110.33	Subtilisin-like serine protease	61%	Q8LAE1	*Arabidopsis thailana*	no	yes
551801*	eugene3.00021116	POPTR_0002s12130.1	82.0/6.6	15/15	148.39	Serine protease	98%	Q8RVJ7	*Populus canadensis*	yes	
675847*	grail3.0028002001	POPTR_0006s14400.1	39.3/6.3	4/4	40.31	Cysteine protease CP1	79%	A5HIJ1	*Actinidia deliciosa*	yes	
781583*	fgenesh4_pg.C_scaffold_40000333	POPTR_0014s02410.1	50.4/5.4	3/3	20.16	Cysteine protease CP1	82%	Q52QX8	*Manihot esculenta*	yes	
560367*	eugene3.00060326	POPTR_0006s03490.1	51.1/4.9	9/8	88.24	Serine carboxypeptidase family protein	65%	Q2R3G8	*Oryza sativa subsp. japonica*	yes	
835003*	estExt_fgenesh4_pm.C_LG_XIV0520	POPTR_0014s17580.1	55.6/5.4	7/6	60.21	Serine carboxypeptidase-like 20	72%	Q8L7B2	*Arabidopsis thaliana*	yes	
199556[#*]	gw1.IX.21.1	POPTR_0009s00820.1	49.0/5.4	6/6	60.20	Serine carboxypeptidase-like 51	67%	Q67Y83	*Arabidopsis thaliana*	no	yes

Table S2. continued

249095#*	gw1.XIX.1495.1	POPTR_0019s08160.1	52.4/5.2	5/4	50.27	Wound-inducible carboxypeptidase	64%	Q9M513	*Solanum lycopersicum*	no	yes
751613	fgenesh4_pg.C_LG_I001216	POPTR_0001s22090.1	55.8/5.3	3/1	30.25	Prolylcarboxypeptidase-like protein	58%	Q8LFB7	*Arabidopsis thaliana*	yes	
825802*	estExt_fgenesh4_pg.C_LG_XVIII0531	POPTR_0018s03020.1	49.9/7.4	10/7	90.39	41 kD chloroplast nucleoid DNA binding protein	45%	Q8H9F4	*Nicotiana sylvestris*	yes	
571847	eugene3.00131211	POPTR_0013s14800.1	90.4/6.2	1/1	8.19	Cell survival CED-4-interacting protein MAC-1	61%	Q5VRN1	*Oryza sativa subsp. japonica*	no	no

Carbohydrate metabolism

575698*	eugene3.00151093	POPTR_0015s14380.1	47.9/5.7	3/3	20.16	Enolase	100%	A9PD49	*Populus trichocarpa*	no	yes
772214*	fgenesh4_pg.C_LG_XI001367	POPTR_0011s16170.1	59.6/6.1	9/8	60.23	Carbohydrate oxidase	59%	Q8SA59	*Helianthus annuus*	yes	
286987#*	gw1.40.747.1	POPTR_0014s02940.1	43.7/4.8	5/1	50.27	Alpha-amylase	77%	Q7X9T1	*Phaseolus angularis*	no	yes
644125*	grail3.0033012902	POPTR_0515s00220.2	44.1/5.1	15/5	120.27	Alpha-amylase	78%	Q7X9T1	*Phaseolus angularis*	no	yes

Other metabolic processes

816232*	estExt_fgenesh4_pg.C_LG_II0662	POPTR_0002s07290.1	41.1/6.3	4/3	40.35	Alcohol dehydrogenase 2	87%	Q9FZ01	*Vitis vinifera*	no	no
828210	estExt_fgenesh4_pg.C_1450022	POPTR_0018s07000.1	53.5/5.8	3/3	20.14	Nucleotide pyrophosphatase-like protein	70%	Q9FS13	*Spinacia oleracea*	SA	yes
652386	grail3.0024007102	POPTR_0006s01080.1	45.6/5.3	1/1	8.14	Fumarylacetoacetase	77%	Q2HT34	*Medicago truncatula*	no	yes
755459	fgenesh4_pg.C_LG_II001639	POPTR_0002s17860.1	58.8/9.0	1/1	8.14	3-ketoacyl-CoA synthase 10	82%	A9XU46	*Gossypium hirsutum*	SA	no
580100	eugene3.01180035	POPTR_0003s08760.1	17.8/5.1	2/2	10.21	Mitochondrial glycine decarboxylase complex H-protein	98%	Q6UJ35	*Populus tremuloides*	yes	
204539#	gw1.IX.5004.1	POPTR_0009s01140.1	74.6/7.3	1/1	8.16	DNA ligase	81%	A7PQQ0	*Vitis vinifera*	no	no
418463#	gw1.VI.2836.1	POPTR_0006s01000.1	25.9/4.8	1/1	10.14	ABC transporter	73%	Q65CB1	*Populus tremula x Populus tremuloides*	no	yes
821987	estExt_fgenesh4_pg.C_LG_X0820	POPTR_0010s10020.1	20.0/9.4	1/1	10.24	F9D12.16 protein (Copper binding protein-like, GPI-anchored protein)	53%	O81500	*Arabidopsis thaliana*	yes	
419378#	gw1.VIII.806.1	POPTR_0008s15040.1	16.8/9.0	2/2	28.27	F9D12.16 protein (Copper binding protein-like, GPI-anchored protein)	60%	O81500	*Arabidopsis thaliana*	yes	
417410#	gw1.VI.1783.1	POPTR_0006s06650.1	31.6/5.2	1/1	10.26	Dicyanin	44%	Q9M510	*Solanum lycopersicum*	yes	
652151*	grail3.0002074001	POPTR_0005s26740.1	17.0/5.1	4/3	40.27	Plastocyanin A	100%	P00299	*Populus nigra*	no	yes
813630	estExt_fgenesh4_kg.C_LG_II0003	POPTR_0002s01740.1	17.0/4.9	5/4	40.23	Plastocyanin B	98%	P11970	*Populus nigra*	yes	
574226	eugene3.00190700	POPTR_0019s10720.1	108.0/5.5	1/1	10.13	Leucine-rich receptor-like protein kinase	59%	Q9C7T7	*Arabidopsis thaliana*	yes	
294068#	gw1.66.741.1	POPTR_0004s21990.1	70.2/5.2	1/1	8.15	E3 SUMO-protein ligase SIZ1	68%	Q680Q4	*Arabidopsis thaliana*	no	no
802937	fgenesh4_pm.C_LG_VII000423	POPTR_0007s13010.2	83.3/6.0	1/1	8.14	Bg55 protein	60%	Q93XR9	*Bruguiera gymnorhiza*	no	no
177387#	gw1.I.5987.1	POPTR_0001s46710.1	56.8/6.0	8/5	50.26	At1g30760/T5I8_22	66%	Q93ZA3	*Arabidopsis thaliana*	no	no

Nutrient assimilation

566952	eugene3.00101510	POPTR_0010s16790.1	72.5/5.8	4/4	40.19	Purple acid phosphatase	67%	Q5MAU7	*Arabidopsis thaliana*	yes	
414694#	gw1.III.1797.1	POPTR_0003s17460.1	56.6/5.1	4/3	26.29	Purple acid phosphatase	79%	Q6TPH1	*Arabidopsis thaliana*	no	yes
820480	estExt_fgenesh4_pg.C_LG_VIII0748	POPTR_0008s08640.1	25.1/5.8	5/5	40.29	S-like ribonuclease	76%	Q9LLS1	*Prunus dulcis*	yes	

Unclassified

216788#	gw1.VII.1093.1	POPTR_0421s00220.1	19.2/9.1	9/2	94.22	Cysteine-rich repeat secretory protein 38	56%	Q9LRJ9	*Arabidopsis thaliana*	no	no
292397#	gw1.64.49.1	POPTR_0017s06760.1	23.8/6.9	1/1	10.12	Cysteine-rich repeat secretory protein 38	53%	Q9LRJ9	*Arabidopsis thaliana*	no	no
640081#*	grail3.0008013701	POPTR_0001s13320.1	17.4/9.6	3/3	30.26	Leucine-rich repeat protein	67%	A5HKK5	*Nicotiana tabacum*	no	yes
293740#	gw1.66.413.1	POPTR_0004s20970.1	57.9/8.7	1/1	8.13	AT3g11590/F24K9_26	51%	Q945L6	*Arabidopsis thaliana*	no	yes
832859	estExt_fgenesh4_pm.C_LG_VIII0763	POPTR_0008s18160.1	29.4/6.7	1/1	8.15	F21J9.10	67%	Q9FYL9	*Arabidopsis thaliana*	no	yes

Table S2. continued

553942	eugene3.00030575	POPTR_0007s10160.1	69.4/9.0	1/1	8.13	Tetratricopeptide repeat (TPR)-containing protein	48%	Q6L4Y6	*Oryza sativa subsp. japonica*	no	yes
252587[#]	gw1.XV.2023.1	POPTR_0015s10470.1	46.6/9.8	1/1	8.14	Auxin-independent growth promoter-like protein	74%	Q9FMW3	*Arabidopsis thaliana*	no	no
549865*	eugene3.00012306	POPTR_0001s31740.1	20.9/8.7	2/2	20.26	Tumor-related protein	37%	P93378	*Nicotiana tabacum*	yes	
814847*	estExt_fgenesh4_pg.C_LG_I0347	POPTR_0001s05560.1	95.2/5.5	34/24	280.34	no sequence similarity to a known protein				yes	
413168[#]	gw1.III.271.1	POPTR_0003s20500.1	95.2/6.4	12/2	98.34	no sequence similarity to a known protein				yes	
588847	eugene3.26820002		71.0/10.9	2/2	8.12	no sequence similarity to a known protein				yes	
791899	fgenesh4_pg.C_scaffold_3039000001		88.5/11.6	1/1	6.18	no sequence similarity to a known protein				no	no

[#] protein sequence in JGI database is incomplete

* protein was also identified via 2D-PAGE MSMS

SA - protein is predicted to serve as signal anchor
