# Supplementary material for: Apoplast proteome reveals that extracellular matrix contributes to multistress response in poplar
Source: BMC Genomics. 2010 Nov 29;11:674. doi: 10.1186/1471-2164-11-674 (PMC3091788; doi:10.1186/1471-2164-11-674)
Supplement: Additional file 4 — Supplementary Table S3. Proteins identified in poplar (P. deltoides) stem apoplast using 2-D PAGE MS/MS. [file 1471-2164-11-674-S4.PDF]

Additional file 4

File format: PDF

Title: Supplementary Table S3

Description:
Table S3. Proteins identified in poplar (*P. deltoides*) stem apoplast using 2-D PAGE MS/MS.

Poplar protein ID	Poplar transcript ID	Poplar new ID	Spot number	Mr/pI (theor.)	Number of matched peptides	Protein score	Protein identity/similarity	Sequence similarity	Accession number	Organism	Signal peptide (SP)	Non-classical SP
**Cell wall metabolism**												
255102*	gw1.XVI.1041.1	POPTR_0016s02620.2	28	73.3/5.5	14	213	Alpha-L-arabinofuranosidase	74%	Q7X9G7	*Malus domestica*	yes	
255102*	gw1.XVI.1041.1	POPTR_0016s02620.2	29	73.3/5.5	10	126	Alpha-L-arabinofuranosidase	74%	Q7X9G7	*Malus domestica*	yes	
718566	estExt_Genewise1_v1.C_LG_VII1401	POPTR_0007s04020.1	44	62.1/6.3	9	99	Pectin methylesterase 2	68%	A0ZNK0	*Pyrus communis*	yes	
816882	estExt_fgenesh4_pg.C_LG_II2363	POPTR_0002s23920.1	55	114.8/5.8	14	175	Alpha-mannosidase	71%	P94078	*Arabidopsis thaliana*	yes	
649422	grail3.0001045402	POPTR_0009s11830.2	60	39.8/6.6	3	94	Glucan endo-1,3-beta-glucosidase 7	73%	Q9M069	*Arabidopsis thaliana*	yes	
830063*	estExt_fgenesh4_pm.C_LG_II0164	POPTR_0002s03580.1	12	34.0/5.5	9	109	Phenylcoumaran benzylic ether reductase	100%	O65904	*Populus trichocarpa*	no	no
830063*	estExt_fgenesh4_pm.C_LG_II0164	POPTR_0002s03580.1	22	34.0/5.5	9	118	Phenylcoumaran benzylic ether reductase	100%	O65904	*Populus trichocarpa*	no	no
830063*	estExt_fgenesh4_pm.C_LG_II0164	POPTR_0002s03580.1	47	34.0/5.5	10	247	Phenylcoumaran benzylic ether reductase	100%	O65904	*Populus trichocarpa*	no	no
830063*	estExt_fgenesh4_pm.C_LG_II0164	POPTR_0002s03580.1	46	34.0/5.5	13	242	Phenylcoumaran benzylic ether reductase	100%	O65904	*Populus trichocarpa*	no	no
830063*	estExt_fgenesh4_pm.C_LG_II0164	POPTR_0002s03580.1	49	34.0/5.5	13	236	Phenylcoumaran benzylic ether reductase	100%	O65904	*Populus trichocarpa*	no	no
830063*	estExt_fgenesh4_pm.C_LG_II0164	POPTR_0002s03580.1	1	34.0/5.5	15	298	Phenylcoumaran benzylic ether reductase	100%	O65904	*Populus trichocarpa*	no	no
830063*	estExt_fgenesh4_pm.C_LG_II0164	POPTR_0002s03580.1	50	34.0/5.5	12	239	Phenylcoumaran benzylic ether reductase	100%	O65904	*Populus trichocarpa*	no	no
830063*	estExt_fgenesh4_pm.C_LG_II0164	POPTR_0002s03580.1	48	34.0/5.5	14	274	Phenylcoumaran benzylic ether reductase	100%	O65904	*Populus trichocarpa*	no	no
830063*	estExt_fgenesh4_pm.C_LG_II0164	POPTR_0002s03580.1	21	34.0/5.5	13	194	Phenylcoumaran benzylic ether reductase	100%	O65904	*Populus trichocarpa*	no	no
**Cell wall and stress related**												
547681*	eugene3.00010122	POPTR_0001s05050.1	23	36.8/4.5	4	292	Peroxidase	98%	Q43101	*Populus trichocarpa*	yes	
817692*	estExt_fgenesh4_pg.C_LG_III1871	POPTR_0003s21620.1	37	37.2/4.5	6	380	Peroxidase	95%	Q43049	*Populus kitakamiensis*	yes	
413562[#] *	gw1.III.665.1	POPTR_0003s21640.1	37	32.8/4.7	6	356	Peroxidase	100%	Q43102	*Populus trichocarpa*	no	yes
817692*	estExt_fgenesh4_pg.C_LG_III1871	POPTR_0003s21620.1	38	37.2/4.5	6	94	Peroxidase	95%	Q43049	*Populus kitakamiensis*	yes	
413562[#] *	gw1.III.665.1	POPTR_0003s21640.1	38	32.8/4.7	7	71	Peroxidase	100%	Q43102	*Populus trichocarpa*	no	yes
817694*	estExt_fgenesh4_pg.C_LG_III1873	POPTR_0003s21660.1	58	33.4/5.8	10	282	Peroxidase N	67%	Q42517	*Armoracia rusticana*	no	yes
800693*	fgenesh4_pm.C_LG_IV000380	POPTR_0004s14240.1	73	34.6/5.5	6	124	Peroxidase ATP17a like protein	68%	Q67XK7	*Arabidopsis thaliana*	yes	
589413*	eugene3.00280149	POPTR_0006s13190.1	20	36.9/5.7	8	75	Peroxidase 21	71%	Q42580	*Arabidopsis thaliana*	yes	
208491[#] *	gw1.V.3892.1	POPTR_0005s14190.1	57	32.6/5.2	11	142	Peroxidase 10	66%	Q9FX85	*Arabidopsis thaliana*	no	yes
**Stress/defense**												
746640*	estExt_Genewise1_v1.C_1970084	POPTR_0015s05990.1	18	30.8/4.4	5	182	Acidic class III chitinase	71%	Q09Y38	*Citrullus lanatus*	yes	
290846[#]	gw1.5405.1.1	POPTR_0001s26210.1	79	34.7/4.7	6	102	Beta-1,3-glucanase	69%	Q84RT6	*Fragaria ananassa*	no	yes
821619*	estExt_fgenesh4_pg.C_LG_IX1399	POPTR_0009s02070.1	77	27.3/5.5	9	291	Cytosolic ascorbate peroxidase 1	88%	A7KIX5	*Gossypium hirsutum*	no	no
821619*	estExt_fgenesh4_pg.C_LG_IX1399	POPTR_0009s02070.1	105	27.3/5.5	14	270	Cytosolic ascorbate peroxidase 1	88%	A7KIX5	*Gossypium hirsutum*	no	no
811643*	fgenesh4_pm.C_scaffold_163000009	POPTR_0011s01280.1	90	21.6/6.4	8	314	Cu-Zn superoxide dismutase	99%	A9PJW9	*Populus jackii*	no	yes
595511*	eugene3.00700152	POPTR_0005s04590.1	11	15.3/5.6	6	288	Superoxide dismutase [Cu-Zn]	100%	A3FM77	*Populus trichocarpa*	no	yes
595511*	eugene3.00700152	POPTR_0005s04590.1	91	15.3/5.6	4	93	Superoxide dismutase [Cu-Zn]	100%	A3FM77	*Populus trichocarpa*	no	yes
727757*	estExt_Genewise1_v1.C_LG_XI2337	POPTR_0011s03570.1	7	21.7/6.1	6	266	Benzoquinone reductase	85%	A3F7Q3	*Gossypium hirsutum*	no	no
663306*	grail3.0055008002	POPTR_0001s43940.1	5	21.6/5.8	8	283	Benzoquinone reductase	91%	A3F7Q3	*Gossypium hirsutum*	no	no

Table S3. continued

726993	estExt_Genewise1_v1.C_LG_XI0270	POPTR_0011s13310.1	87	21.7/6.0	7	371	Benzoquinone reductase	90%	A3F7Q3	*Gossypium hirsutum*	no	no
813818*	estExt_fgenesh4_kg.C_LG_IV0063	POPTR_0004s17610.1	39	18.1/8.7	6	71	Peptidyl-prolyl *cis-trans* isomerase	100%	A9P8L4	*Populus trichocarpa*	no	no
813818*	estExt_fgenesh4_kg.C_LG_IV0063	POPTR_0004s17610.1	40	18.1/8.7	10	234	Peptidyl-prolyl *cis-trans* isomerase	100%	A9P8L4	*Populus trichocarpa*	no	no
813818*	estExt_fgenesh4_kg.C_LG_IV0063	POPTR_0004s17610.1	13	18.1/8.7	7	90	Peptidyl-prolyl *cis-trans* isomerase	100%	A9P8L4	*Populus trichocarpa*	no	no
179209[#]	gw1.I.7809.1	POPTR_0001s13480.1	99	19.2/8.0	7	157	Peptidyl-prolyl *cis-trans* isomerase	91%	A7QUU7	*Vitis vinifera*	no	no
643603*	grail3.0003069401	POPTR_0002s08260.1	45	56.2/4.8	20	266	Protein disulfide-isomerase	76%	Q43116	*Ricinus communis*	yes	
832078	estExt_fgenesh4_pm.C_LG_VI0650	POPTR_0006s24030.1	98	17.5/6.2	4	135	Cytosolic class II low molecular weight heat shock protein	88%	Q9XGS6	*Prunus dulcis*	no	no
563962	eugene3.00080557	POPTR_0008s06260.1	100	18.3/6.2	11	152	18.5 kDa class I heat shock protein	80%	P05478	*Glycine max*	no	yes
563887	eugene3.00080482	POPTR_0008s05410.1	30	71.2/5.1	14	110	DnaK-type molecular chaperone hsp70	95%	Q53NM9	*Oryza sativa subsp. japonica*	no	no
198984[#] *	gw1.IV.4073.1	POPTR_0004s10240.1	80	26.6/9.1	8	112	Mangrin	73%	Q9AYT8	*Bruguiera sexangula*	no	no
729432*	estExt_Genewise1_v1.C_LG_XIII0635	POPTR_0013s01090.1	51	47.4/4.4	17	315	Calreticulin	86%	P93508	*Ricinus communis*	yes	
811231*	fgenesh4_pm.C_scaffold_133000042	POPTR_0005s01850.1	51	43.8/4.6	9	111	Calreticulin-1	88%	O81919	*Beta vulgaris*	yes	
811231*	fgenesh4_pm.C_scaffold_133000042	POPTR_0005s01850.1	52	43.8/4.6	12	260	Calreticulin-1	88%	O81919	*Beta vulgaris*	yes	
729432*	estExt_Genewise1_v1.C_LG_XIII0635	POPTR_0013s01090.1	52	47.4/4.4	7	64	Calreticulin	86%	P93508	*Ricinus communis*	yes	
729432*	estExt_Genewise1_v1.C_LG_XIII0635	POPTR_0013s01090.1	53	47.4/4.4	14	312	Calreticulin	86%	P93508	*Ricinus communis*	yes	
811231*	fgenesh4_pm.C_scaffold_133000042	POPTR_0005s01850.1	53	43.8/4.6	7	141	Calreticulin-1	88%	O81919	*Beta vulgaris*	yes	
243514[#]	gw1.XIV.257.1	POPTR_0483s00220.1	53	24.2/5.7	7	122	Glutathione S-transferase	63%	A0T2X7	*Vitis vinifera*	no	yes
665133	grail3.0104000601	POPTR_0002s20890.1	8	23.2/6.2	5	61	Glutathione S-transferase	63%	A0T2X7	*Vitis vinifera*	no	no
819386*	estExt_fgenesh4_pg.C_LG_VI1270	POPTR_0006s19310.1	106	13.8/8.9	6	208	Blight-associated protein p12	49%	Q6K4C4	*Oryza sativa subsp. japonica*	yes	
573766	eugene3.00190240	POPTR_0019s04070.1	101	21.9/8.3	8	179	Peroxiredoxin	76%	Q5JBR7	*Ipomoea batatas*	no	yes
749645	fgenesh4_kg.C_LG_IX000057	POPTR_0009s01280.1	67	20.8/5.6	13	159	Glyoxalase I	87%	A5A8J4	*Cucurbita maxima*	no	no
201272	gw1.IX.1737.1	POPTR_0009s12090.1	49	34.0/7.8	2	71	Isoflavone reductase related protein	80%	O81355	*Pyrus communis*	no	yes
201272	gw1.IX.1737.1	POPTR_0009s12090.1	48	34.1/7.8	2	79	Isoflavone reductase related protein	80%	O81355	*Pyrus communis*	no	yes
201272	gw1.IX.1737.1	POPTR_0009s12090.1	1	34.0/7.8	2	71	Isoflavone reductase related protein	80%	O81355	*Pyrus communis*	no	yes

Proteolysis

551801*	eugene3.00021116	POPTR_0002s12130.1	34	82.0/6.6	12	132	Serine protease	98%	Q8RVJ7	Populus canadensis	yes	
781583*	fgenesh4_pg.C_scaffold_40000333	POPTR_0014s02410.1	78	50.4/5.4	9	389	Cysteine protease CP1	82%	Q52QX8	*Manihot esculenta*	yes	
675847	grail3.0028002001	POPTR_0006s14400.1	81	39.3/6.3	5	124	Cysteine protease CP1	79%	A5HIJ1	*Actinia deliciosa*	yes	
675847	grail3.0028002001	POPTR_0006s14400.1	82	39.3/6.3	3	66	Cysteine protease CP1	79%	A5HIJ1	*Actinia deliciosa*	yes	
262677*	gw1.XVIII.3218.1	POPTR_0018s11600.1	63	55.1/5.9	14	214	Leucine aminopeptidase 1	77%	P30184	*Arabidopsis thaliana*	no	yes
708470*	estExt_Genewise1_v1.C_LG_I7028	POPTR_0001s06560.1	54	45.6/9.5	15	313	Nucleoid DNA-binding-like protein	66%	Q8L934	*Arabidopsis thaliana*	yes	

Carbohydrate metabolism

656103*	grail3.0049021504	POPTR_0008s05640.1	6	27.2/6.0	15	480	Triosephosphate isomerase	100%	A9PE68	*Populus trichocarpa*	no	yes
656103*	grail3.0049021504	POPTR_0008s05640.1	84	27.2/6.0	14	382	Triosephosphate isomerase	100%	A9PE68	*Populus trichocarpa*	no	yes
724697*	estExt_Genewise1_v1.C_LG_X2172	POPTR_0010s21100.1	86	27.4/6.5	9	312	Triosephosphate isomerase	100%	A9P7V6	*Populus trichocarpa*	no	no
724697*	estExt_Genewise1_v1.C_LG_X2172	POPTR_0010s21100.1	85	27.4/6.5	8	171	Triosephosphate isomerase	100%	A9P7V6	*Populus trichocarpa*	no	no
822907	estExt_fgenesh4_pg.C_LG_XI0680	POPTR_0011s09860.1	10	35.6/8.7	15	345	Malate dehydrogenase	100%	A9PCR0	*Populus trichocarpa*	no	no
564942*	eugene3.00081537	POPTR_0008s16670.1	66	35.7/6.1	11	117	Malate dehydrogenase	100%	A9P8R3	*Populus trichocarpa*	no	yes

Table S3. continued

564942*	eugene3.00081537	POPTR_0008s16670.1	65	35.7/6.1	18	308	Malate dehydrogenase	100%	A9P8R3	*Populus trichocarpa*	no	yes
707785	estExt_Genewise1_v1.C_LG_I4975	POPTR_0001s38560.1	70	35.5/8.0	11	266	Malate dehydrogenase	93%	A9PCR0	*Populus trichocarpa*	no	no
707785	estExt_Genewise1_v1.C_LG_I4975	POPTR_0001s38560.1	68	35.5/8.0	11	266	Malate dehydrogenase	93%	A9PCR0	*Populus trichocarpa*	no	no
575698*	eugene3.00151093	POPTR_0015s14380.1	9	47.9/5.7	18	192	Enolase	100%	A9PD49	*Populus trichocarpa*	no	yes
836259*	estExt_fgenesh4_pm.C_280132	POPTR_0006s11800.1	61	47.6/5.6	8	64	Enolase	100%	A9PIJ2	*Populus trichocarpa*	no	yes
575698*	eugene3.00151093	POPTR_0015s14380.1	31	47.9/5.7	11	136	Enolase	100%	A9PD49	*Populus trichocarpa*	no	yes
575698*	eugene3.00151093	POPTR_0015s14380.1	32	47.9/5.7	21	403	Enolase	100%	A9PD49	*Populus trichocarpa*	no	yes
836259*	estExt_fgenesh4_pm.C_280132	POPTR_0006s11800.1	32	47.6/5.6	6	133	Enolase	100%	A9PIJ2	*Populus trichocarpa*	no	yes
575698*	eugene3.00151093	POPTR_0015s14380.1	2	47.9/5.7	20	371	Enolase	100%	A9PD49	*Populus trichocarpa*	no	yes
836259*	estExt_fgenesh4_pm.C_280132	POPTR_0006s11800.1	2	47.6/5.6	6	133	Enolase	100%	A9PIJ2	*Populus trichocarpa*	no	yes
564181*	eugene3.00080776	POPTR_0008s08400.1	64	42.5/5.8	10	118	Phosphoglycerate kinase	99%	A9P828	*Populus trichocarpa*	no	no
564181*	eugene3.00080776	POPTR_0008s08400.1	3	42.5/5.8	13	184	Phosphoglycerate kinase	99%	A9P828	*Populus trichocarpa*	no	no
564181*	eugene3.00080776	POPTR_0008s08400.1	24	42.5/5.8	13	260	Phosphoglycerate kinase	99%	A9P828	*Populus trichocarpa*	no	no
659332*	grail3.0154005402	POPTR_0010s17870.1	36	42.7/5.7	8	75	Phosphoglycerate kinase	99%	O82159	*Populus nigra*	no	no
659332*	grail3.0154005402	POPTR_0010s17870.1	104	42.7/5.7	19	318	Phosphoglycerate kinase	99%	O82159	*Populus nigra*	no	no
825441*	estExt_fgenesh4_pg.C_LG_XVI1334	POPTR_0016s14950.1	26	61.1/5.4	29	496	2,3-bisphosphoglycerate-independent phosphoglycerate mutase	90%	P35493	*Ricinus communis*	no	no
593790	eugene3.00570034	POPTR_0005s10990.1	35	98.2/5.9	18	99	Aconitate hydratase 1	90%	Q42560	*Arabidopsis thailana*	no	no
592888	eugene3.00440223	POPTR_0017s01390.1	74	51.6/5.8	20	510	UDP-glucose pyrophosphorylase	98%	Q2V506	*Populus tremula x Populus tremuloides*	no	no

Other metabolic processes

835828	estExt_fgenesh4_pm.C_LG_XVIII0158	POPTR_0018s02250.1	75	50.9/8.7	20	183	Aspartate aminotransferase	89%	A5AZ93	*Vitis vinifera*	no	yes
720428	estExt_Genewise1_v1.C_LG_VIII1306	POPTR_0008s12550.1	103	59.9/5.9	11	66	ATP synthase subunit beta	88%	O82722	*Nicotiana sylvestris*	no	yes
720428	estExt_Genewise1_v1.C_LG_VIII1306	POPTR_0008s12550.1	56	59.9/5.9	14	116	ATP synthase subunit beta	88%	O82722	*Nicotiana sylvestris*	no	yes
644907	grail3.0050014702	POPTR_0002s19000.1	62	43.2/5.6	17	260	S-adenosylmethionine synthetase	98%	A9P822	*Populus trichocarpa*	no	no
823550	estExt_fgenesh4_pg.C_LG_XII1303	POPTR_0012s14980.1	72	25.6/5.5	8	122	Proteasome subunit alpha type	94%	A9PAG0	*Populus trichocarpa*	no	no
734681	estExt_Genewise1_v1.C_LG_XVI1054	POPTR_0016s02790.1	59	46.5/5.4	18	297	26S protease regulatory subunit 6B homolog	92%	Q9SEI4	*Arabidopsis thaliana*	no	yes
560773	eugene3.00060732	POPTR_0006s08710.1	69	27.0/5.7	11	277	3-beta hydroxysteroid dehydrogenase/isomerase protein	82%	Q94HJ5	*Oryza sativa*	no	yes
738638	estExt_Genewise1_v1.C_LG_XIX1453	POPTR_0019s08100.1	88	17.7/5.4	10	225	S-adenosylmethionine:2-demethylmenaquinone methyltransferase-like	89%	Q8W0C5	*Oryza sativa subsp. japonica*	no	no
816369*	estExt_fgenesh4_pg.C_LG_II0927	POPTR_0002s10150.1	89	18.8/5.6	1	108	Blue copper-like protein	55%	A3F8V0	*Gossypium hirsutum*	yes	
816369*	estExt_fgenesh4_pg.C_LG_II0927	POPTR_0002s10150.1	93	18.8/5.6	3	101	Blue copper-like protein	55%	A3F8V0	*Gossypium hirsutum*	yes	
816369*	estExt_fgenesh4_pg.C_LG_II0927	POPTR_0002s10150.1	43	18.8/5.6	1	153	Blue copper-like protein	55%	A3F8V0	*Gossypium hirsutum*	yes	
816369*	estExt_fgenesh4_pg.C_LG_II0927	POPTR_0002s10150.1	42	18.8/5.6	3	92	Blue copper-like protein	55%	A3F8V0	*Gossypium hirsutum*	yes	
816369*	estExt_fgenesh4_pg.C_LG_II0927	POPTR_0002s10150.1	94	18.8/5.6	2	117	Blue copper-like protein	55%	A3F8V0	*Gossypium hirsutum*	yes	
816369*	estExt_fgenesh4_pg.C_LG_II0927	POPTR_0002s10150.1	92	18.8/5.6	3	186	Blue copper-like protein	55%	A3F8V0	*Gossypium hirsutum*	yes	
816369*	estExt_fgenesh4_pg.C_LG_II0927	POPTR_0002s10150.1	14	18.8/5.6	3	126	Blue copper-like protein	55%	A3F8V0	*Gossypium hirsutum*	yes	
816369*	estExt_fgenesh4_pg.C_LG_II0927	POPTR_0002s10150.1	17	18.8/5.6	3	180	Blue copper-like protein	55%	A3F8V0	*Gossypium hirsutum*	yes	
816369*	estExt_fgenesh4_pg.C_LG_II0927	POPTR_0002s10150.1	16	18.8/5.6	3	136	Blue copper-like protein	55%	A3F8V0	*Gossypium hirsutum*	yes	
174402[#] *	gw1.I.3002.1	POPTR_0001s33960.1	95	11.9/8.5	2	66	Blue copper protein	70%	Q8LED5	*Arabidopsis thaliana*	yes	
591761	eugene3.00400106	POPTR_0014s04880.1	97	16.3/6.1	6	102	Nucleoside diphosphate kinase	100%	A9PAF2	*Populus trichocarpa*	no	yes
591761	eugene3.00400106	POPTR_0014s04880.1	96	16.3/6.1	7	75	Nucleoside diphosphate kinase	100%	A9PAF2	*Populus trichocarpa*	no	yes

Table S3. continued

256059	gw1.XVI.1998.1	POPTR_0016s06310.1	107	22.6/9.8	5	67	40S ribosomal protein S5	91%	O65731	*Cicer arietinum*	no	no
727272	estExt_Genewise1_v1.C_LG_XI0817	POPTR_0011s15170.1	107	21.9/9.5	5	64	60S ribosomal protein L9	86%	P30707	*Pisum sativum*	no	yes
**Cell structure**												
813612	estExt_fgenesh4_kg.C_LG_I0082	POPTR_0001s31700.1	25	41.7/5.3	18	527	Actin	99%	A4ZSZ4	*Populus trichocarpa*	no	no
707456	estExt_Genewise1_v1.C_LG_I4174	POPTR_0001s29670.1	83	49.6/4.9	8	67	Alpha-tubulin 3	99%	A5YRY0	*Populus tremuloides*	no	no
**Unclassified**												
828728	estExt_fgenesh4_pg.C_2100027	POPTR_0008s22310.1	41	19.1/4.5	7	278	Translationally controlled tumor-like protein	85%	Q06H31	*Arachis hypogaea*	no	yes
732264*	estExt_Genewise1_v1.C_LG_XIV3377	POPTR_0014s15330.1	71	26.2/5.2	10	164	Carboxymethylenebutenolidase	84%	O80889	*Arabidopsis thaliana*	no	no
732264*	estExt_Genewise1_v1.C_LG_XIV3377	POPTR_0014s15330.1	4	26.2/5.2	11	161	Carboxymethylenebutenolidase	84%	O80889	*Arabidopsis thaliana*	no	no
732264*	estExt_Genewise1_v1.C_LG_XIV3377	POPTR_0014s15330.1	19	26.2/5.2	10	167	Carboxymethylenebutenolidase	84%	O80889	Arabidopsis thaliana	no	no
718495	estExt_Genewise1_v1.C_LG_VII1106	POPTR_1698s00200.1	83	26.4/5.8	9	236	Cysteine-rich repeat secretory protein 38	58%	Q9LRJ9	*Arabidopsis thaliana*	yes	
814847*	estExt_fgenesh4_pg.C_LG_I0347	POPTR_0001s05560.1	15	95.2/5.5	9	262	no sequence similarity to a known protein				yes	

[#] protein sequence in JGI database is incomplete

* protein was also identified via 2D-LC MSMS
