# Supplementary material for: Apoplast proteome reveals that extracellular matrix contributes to multistress response in poplar
Source: BMC Genomics. 2010 Nov 29;11:674. doi: 10.1186/1471-2164-11-674 (PMC3091788; doi:10.1186/1471-2164-11-674)
Supplement: Additional file 5 — Supplementary Table S4. Proteins identified in poplar (P. deltoides) stem apoplast using 2-D LC MS/MS. [file 1471-2164-11-674-S5.PDF]

Additional file 5

File format: PDF

Title: Supplementary Table S4

Description:
Table S4. Proteins identified in poplar (*P. deltoides*) stem apoplast using 2-D LC MS/MS.

Poplar Protein ID	Poplar transcript ID	Poplar new ID	Mr/pI (theor.)	Number of matched peptides total/unique	Protein score	Protein identity/similarity	Sequence similarity	Accession number	Organism	Signal peptide (SP)	Non-classical SP
**Cell wall metabolism**											
255102*	gw1.XVI.1041.1	POPTR_0016s02620.2	73.3/5.5	17/10	120.37	Alpha-L-arabinofuranosidase	74%	Q7X9G7	*Malus domestica*	yes	
830063*	estExt_fgenesh4_pm.C_LG_II0164	POPTR_0002s03580.1	34.0/5.5	14/7	100.28	Phenylcoumaran benzylic ether reductase	100%	O65904	*Populus trichocarpa*	no	
834247	estExt_fgenesh4_pm.C_LG_XII0129	POPTR_0012s00670.1	39.7/5.5	17/13	150.33	Caffeic acid 3-O-methyltransferase 1	100%	A9P830	*Populus trichocarpa*	no	yes
730906	estExt_Genewise1_v1.C_LG_XIV0850	POPTR_0014s06740.1	43.3/5.4	9/9	60.28	Fasciclin-like arabinogalactan protein 10	68%	Q9LZX4	*Arabidopsis thaliana*	yes	
176165#	gw1.I.4765.1	POPTR_0001s37650.1	37.2/5.9	3/3	20.13	Fasciclin-like arabinogalactan protein 10	77%	A9XTL5	*Gossypium hirsutum*	no	no
825018	estExt_fgenesh4_pg.C_LG_XVI0181	POPTR_0016s02620.1	21.5/6.4	4/1	30.34	Alpha-L-arabinofuranosidase	83%	Q9SG80	*Arabidopsis thaliana*	no	no
575682	eugene3.00151077	POPTR_0015s14570.1	25.4/6.7	3/2	30.33	Fasciclin-like AGP 15	97%	Q6J189	*Populus tremula x Populus alba*	yes	
644497	grail3.0039009101	POPTR_0002s16390.1	51.5/5.9	3/2	20.26	Polygalacturonase-like protein	80%	Q84LI7	*Fragaria ananassa*	yes	
548424	eugene3.00010865	POPTR_0001s09790.1	52.6/5.2	3/1	20.22	Polygalacturonase-like protein	75%	Q84LI7	*Fragaria ananassa*	SA	
728650	estExt_Genewise1_v1.C_LG_XII0499	POPTR_0012s14510.1	25.4/5.3	2/1	20.25	Fasciclin-like AGP 15	89%	Q6J189	*Populus tremula x Populus alba*	yes	
723575	estExt_Genewise1_v1.C_LG_IX4802	POPTR_0009s01740.1	26.9/7.8	5/3	40.28	Fasciclin-like AGP 8	86%	Q6J196	*Populus tremula x Populus alba*	yes	
204338#	gw1.IX.4803.1	POPTR_0009s01780.1	90.1/6.3	2/2	20.26	Beta-galactosidase	77%	Q93X57	Fragaria x ananassa	yes	
706198	estExt_Genewise1_v1.C_LG_I1353	POPTR_0001s24380.1	38.8/8.8	2/2	20.30	UDP-glucuronic acid decarboxylase 3	97%	Q1M0P0	*Populus tomentosa*	no	no
822488	estExt_fgenesh4_pg.C_LG_X1860	POPTR_0010s21420.1	39.0/7.1	2/1	20.25	UDP-glucuronate decarboxylase 1	90%	Q6IVK5	*Nicotiana tabacum*	no	no
822264	estExt_fgenesh4_pg.C_LG_X1411	POPTR_0010s16640.1	21.5/5.4	2/1	20.23	GPI-anchored protein	53%	Q67ZF7	*Arabidopsis thaliana*	yes	
**Cell wall and stress related**											
817694*	estExt_fgenesh4_pg.C_LG_III1873	POPTR_0003s21660.1	33.4/5.8	8/6	70.24	Peroxidase N	67%	Q42517	*Armoracia rusticana*	no	yes
547681*	eugene3.00010122	POPTR_0001s05050.1	36.8/4.5	5/1	50.28	Peroxidase	98%	Q43101	*Populus trichocarpa*	yes	
589413*	eugene3.00280149	POPTR_0006s13190.1	36.9/5.7	8/8	70.22	Peroxidase 21	71%	Q42580	*Arabidopsis thaliana*	yes	
829298	estExt_fgenesh4_pm.C_LG_I0036	POPTR_0001s04850.1	36.6/4.3	5/1	48.28	Peroxidase	100%	Q43100	*Populus trichocarpa*	yes	
555257	eugene3.00031890	POPTR_0003s21610.1	37.1/4.6	3/1	26.25	Peroxidase	95%	Q43050	*Populus kitakamiensis*	yes	
817692*	estExt_fgenesh4_pg.C_LG_III1871	POPTR_0003s21620.1	37.2/4.5	6/1	60.32	Peroxidase	95%	Q43049	*Populus kitakamiensis*	yes	
413562#*	gw1.III.665.1	POPTR_0003s21640.1	32.8/4.7	6/1	60.32	Peroxidase	100%	Q43102	*Populus trichocarpa*	no	yes
824487	estExt_fgenesh4_pg.C_LG_XV0039	POPTR_0015s00580.1	31.4/6.4	2/1	10.14	Peroxidase 1	70%	Q9SSZ9	*Scutellaria baicalensis*	no	yes
208491#*	gw1.V.3892.1	POPTR_0005s14190.1	32.6/5.2	2/1	10.15	Peroxidase 10	66%	Q9FX85	*Arabidopsis thaliana*	no	yes
800693*	fgenesh4_pm.C_LG_IV000380	POPTR_0004s14240.1	34.6/5.5	3/3	20.20	Peroxidase ATP17a like protein	68%	Q67XK7	*Arabidopsis thaliana*	yes	
**Stress/defense**											
746640*	estExt_Genewise1_v1.C_1970084	POPTR_0015s05990.1	30.8/4.4	5/2	50.22	Acidic class III chitinase	71%	Q09Y38	*Citrullus lanatus*	yes	
770285	fgenesh4_pg.C_LG_X001775	POPTR_0010s20360.1	12.6/8.1	2/1	20.19	Protease inhibitor/seed storage/LTP family protein	82%	B0L632	*Cicer arietinum*	yes	
820375	estExt_fgenesh4_pg.C_LG_VIII0518	POPTR_0008s06210.1	12.5/8.6	3/2	30.24	Protease inhibitor/seed storage/LTP family protein	76%	B0L632	*Cicer arietinum*	yes	
825296	estExt_fgenesh4_pg.C_LG_XVI0953	POPTR_0016s10140.1	11.9/8.1	5/5	50.23	Protease inhibitor/seed storage/lipid transfer protein (LTP) family protein	74%	A9XNQ1	*Sonneratia caseolaris*	yes	
414321#	gw1.III.1424.1	POPTR_0003s01440.1	8.4/6.0	2/2	10.18	Non-specific lipid-transfer protein	100%	A9PDS7	*Populus trichocarpa*	no	yes

Table S4. continued

821619*	estExt_fgenesh4_pg.C_LG_IX1399	POPTR_0009s02070.1	27.3/5.5	2/1	20.31	Cytosolic ascorbate peroxidase 1	88%	A7KIX5	*Gossypium hirsutum*	no	no
256724	gw1.XVI.2663.1	POPTR_0016s08580.1	27.0/5.6	3/1	20.24	Cytosolic ascorbate peroxidase	90%	A7LBP6	*Dimocarpus longan*	no	no
595511*	eugene3.00700152	POPTR_0005s04590.1	15.3/5.6	4/4	30.27	Superoxide dismutase [Cu-Zn]	100%	A3FM77	*Populus trichocarpa*	no	yes
811643*	fgenesh4_pm.C_scaffold_163000009	POPTR_0011s01280.1	21.6/6.4	4/3	40.36	Cu-Zn superoxide dismutase	99%	A9PJW9	*Populus jackii*	no	yes
729723	estExt_Genewise1_v1.C_LG_XIII1233	POPTR_0013s03160.1	15.2/5.5	3/1	30.29	Superoxide dismutase [Cu-Zn]	100%	A9PHV3	*Populus trichocarpa*	no	yes
836268	estExt_fgenesh4_pm.C_280146	POPTR_0006s11570.1	47.1/6.5	7/7	48.17	Monodehydroascorbate reductase	88%	A5JPK7	*Vitis vinifera*	no	no
727757*	estExt_Genewise1_v1.C_LG_XI2337	POPTR_0011s03570.1	21.7/6.1	3/*1*	10.23	Benzoquinone reductase	85%	A3F7Q3	*Gossypium hirsutum*	no	no
663306*	grail3.0055008002	POPTR_0001s43940.1	21.6/5.8	5/1	40.21	Benzoquinone reductase	91%	A3F7Q3	*Gossypium hirsutum*	no	no
818813	estExt_fgenesh4_pg.C_LG_V1539	POPTR_0005s26170.1	18.1/9.1	2/1	18.23	Peptidyl-prolyl *cis-trans* isomerase	100%	A9P7Y6	*Populus trichocarpa*	no	no
821113	estExt_fgenesh4_pg.C_LG_IX0365	POPTR_0009s13270.1	18.0/8.7	9/3	70.27	Peptidyl-prolyl *cis-trans* isomerase	100%	A9P8B6	*Populus trichocarpa*	no	no
813818*	estExt_fgenesh4_kg.C_LG_IV0063	POPTR_0004s17610.1	18.1/8.7	10/3	100.28	Peptidyl-prolyl *cis-trans* isomerase	100%	A9P8L4	*Populus trichocarpa*	no	no
643603*	grail3.0003069401	POPTR_0002s08260.1	56.2/4.8	6/5	40.17	Protein disulfide-isomerase	76%	Q43116	*Ricinus communis*	yes	
675629	grail3.0261001101	POPTR_0010s21280.1	71.1/5.1	6/*1*	40.15	Heat shock protein 70-3	94%	Q67BD0	*Nicotiana tabacum*	no	no
198984[#*]	gw1.IV.4073.1	POPTR_0004s10240.1	26.6/9.1	1/1	10.15	Mangrin	73%	Q9AYT8	*Bruguiera sexangula*	no	no
729432*	estExt_Genewise1_v1.C_LG_XIII0635	POPTR_0013s01090.1	47.4/4.4	6/4	40.31	Calreticulin	86%	P93508	*Ricinus communis*	yes	
811231*	fgenesh4_pm.C_scaffold_133000042	POPTR_0005s01850.1	43.8/4.6	5/3	30.19	Calreticulin-1	88%	O81919	*Beta vulgaris*	yes	
819386*	estExt_fgenesh4_pg.C_LG_VI1270	POPTR_0006s19310.1	13.8/8.9	1/1	20.16	Blight-associated protein p12	49%	Q6K4C4	*Oryza sativa subsp. japonica*	yes	
818850	estExt_fgenesh4_pg.C_LG_V1612	POPTR_0005s26930.1	25.8/5.2	4/4	40.34	Dehydrin	97%	A7L2U5	*Populus canadensis*	no	no
833658	estExt_fgenesh4_pm.C_LG_X0557	POPTR_0010s15250.1	28.3/7/6	3/1	30.21	Tropinone reductase-14	66%	B2BXS3	*Boechera divaricarpa*	no	yes
833676	estExt_fgenesh4_pm.C_LG_X0585	POPTR_0010s16070.1	12.5/5.8	3/3	30.35	Protein Pop3	62%	Q9LUV2	*Arabidopsis thaliana*	no	no
282018[#]	gw1.28.592.1	POPTR_0006s13080.1	56.9/6.9	2/1	16.17	Berberine bridge enzyme	59%	Q9FKU8	*Arabidopsis thaliana*	no	no
569448	eugene3.00120101	POPTR_0012s01070.1	59.9/9.4	3/1	26.17	Berberine bridge enzyme	55%	Q9FKU8	*Arabidopsis thaliana*	Signal anchor	yes
233159[#]	gw1.XI.1139.1	POPTR_0011s16220.1	56.1/9.3	4/1	30.29	Berberine bridge enzyme	59%	Q9FKU8	*Arabidopsis thaliana*	no	no
753708	fgenesh4_pg.C_LG_I003311	POPTR_0001s46720.1	27.0/5.9	2/1	18.21	Berberine bridge enzyme	43%	O64743	*Arabidopsis thaliana*	no	no
753715	fgenesh4_pg.C_LG_I003318	POPTR_0001s46770.1	59.7/8.9	4/*1*	30.21	Berberine bridge enzyme	62%	O64743	*Arabidopsis thaliana*	yes	

Proteolysis

551801*	eugene3.00021116	POPTR_0002s12130.1	82.0/6.6	14/14	108.32	Serine protease	98%	Q8RVJ7	*Populus canadensis*	yes	
781583*	fgenesh4_pg.C_scaffold_40000333	POPTR_0014s02410.1	50.4/5.4	2/2	10.23	Cysteine protease CP1	82%	Q52QX8	*Manihot esculenta*	yes	
262677*	gw1.XVIII.3218.1	POPTR_0018s11600.1	55.1/5.9	3/*1*	10.20	Leucine aminopeptidase 1	77%	P30184	*Arabidopsis thaliana*	no	yes
708470*	estExt_Genewise1_v1.C_LG_I7028	POPTR_0001s06560.1	45.6/9.5	1/1	10.13	Nucleoid DNA-binding-like protein	66%	Q8L934	*Arabidopsis thaliana*	yes	
709916	estExt_Genewise1_v1.C_LG_II0461	POPTR_0002s02010.1	77.2/5.3	5/5	30.16	Uncharacterized protein At1g20160.2	61%	Q2V4M5	*Arabidopsis thaliana*	no	yes
417380[#]	gw1.VI.1753.1	POPTR_0006s24090.1	96.8/5.3	5/5	30.21	AT4g33090/F4I10_20 (Aminopeptidase like protein)	75%	Q8VZH2	*Arabidopsis thaliana*	no	yes
548443	eugene3.00010884	POPTR_0001s09600.2	103.5/5.5	5/1	30.20	Aminopeptidase (M1 aminopeptidase)	81%	Q8H0S9	*Arabidopsis thaliana*	no	no
208844	gw1.V.4245.1	POPTR_0005s18880.1	80.4/6.6	1/1	8.17	Subtilisin-like protease	77%	A9XG40	*Nicotiana tabacum*	yes	

Carbohydrate metabolism

656103*	grail3.0049021504	POPTR_0008s05640.1	27.2/6.0	10/5	100.24	Triosephosphate isomerase	100%	A9PE68	*Populus trichocarpa*	no	yes
724697*	estExt_Genewise1_v1.C_LG_X2172	POPTR_0010s21100.1	27.4/6.5	10/4	100.24	Triosephosphate isomerase	100%	A9P7V6	*Populus trichocarpa*	no	no
558071	eugene3.00091331	POPTR_0009s03650.1	28.4/5.4	3/1	30.19	Triosephosphate isomerase	86%	A7NYZ8	*Vitis vinifera*	no	yes
564942*	eugene3.00081537	POPTR_0008s16670.1	35.7/6.1	5/2	30.29	Malate dehydrogenase	100%	A9P8R3	*Populus trichocarpa*	no	yes

Table S4. continued

747123	estExt_Genewise1_v1.C_2730019	POPTR_0010s08180.1	35.7/6.2	4/1	30.22	Malate dehydrogenase	97%	A9P8R3	*Populus trichocarpa*	yes	
575698*	eugene3.00151093	POPTR_0015s14380.1	47.9/5.7	17/14	140.31	Enolase	100%	A9PD49	*Populus trichocarpa*	no	yes
836259*	estExt_fgenesh4_pm.C_280132	POPTR_0006s11800.1	47.6/5.6	9/6	60.21	Enolase	100%	A9PIJ2	*Populus trichocarpa*	no	yes
575307	eugene3.00150702	POPTR_0015s10330.2	36.8/7.0	10/2	70.23	Glyceraldehyde-3-phosphate dehydrogenase	89%	Q9XG67	*Nicotiana tabacum*	no	no
821843	estExt_fgenesh4_pg.C_LG_X0484	POPTR_0010s06560.1	37.1/7.7	6/1	36.23	Glyceraldehyde-3-phosphate dehydrogenase	99%	Q3LUR8	*Populus maximowiczii x Populus nigra*	no	no
728998	estExt_Genewise1_v1.C_LG_XII1463	POPTR_0012s09570.1	36.7/6.3	11/4	90.28	Glyceraldehyde-3-phosphate dehydrogenase	90%	Q9XG67	*Nicotian tabacum*	no	no
564181*	eugene3.00080776	POPTR_0008s08400.1	42.5/5.8	7/2	50.22	Phosphoglycerate kinase	99%	A9P828	*Populus trichocarpa*	no	no
659332*	grail3.0154005402	POPTR_0010s17870.1	42.7/5.7	10/6	80.22	Phosphoglycerate kinase	99%	O82159	*Populus nigra*	no	no
825441*	estExt_fgenesh4_pg.C_LG_XVI1334	POPTR_0016s14950.1	61.1/5.4	8/2	40.19	2,3-bisphosphoglycerate-independent phosphoglycerate mutase	90%	P35493	*Ricinus communis*	no	no
739764	estExt_Genewise1_v1.C_281127	POPTR_0006s11400.1	61.0/5.4	9/3	58.19	2,3-bisphosphoglycerate-independent phosphoglycerate mutase	89%	P35493	*Ricinus communis*	no	no
832763	estExt_fgenesh4_pm.C_LG_VIII0571	POPTR_0010s11970.1	63.1/5.5	10/4	50.23	Phosphoglucomutase, cytoplasmic (Glucose phosphomutase)	98%	Q9ZSQ4	*Populus tremula*	no	no
578574	eugene3.00180798	POPTR_0018s09730.1	38.4/6.6	3/2	20.27	Fructose-bisphosphate aldolase	100%	A9PHA1	*Populus trichocarpa*	no	no
835735	estExt_fgenesh4_pm.C_LG_XVIII0009	POPTR_0018s07380.1	92.5/6.2	3/1	30.13	Sucrose synthase	94%	Q69FD8	*Populus tremuloides*	no	no
181144[#]	gw1.I.9744.1	POPTR_0001s23330.1	54.4/5.4	2/2	20.20	P66 protein	56%	Q84L69	*Hevea brasiliensis*	no	no
640120	grail3.0008017101	POPTR_0001s12930.1	47.8/5.5	3/2	30.20	Transaldolase-like protein	82%	Q38HS6	*Solanum tuberosum*	no	yes
**Other metabolic processes**											
738500	estExt_Genewise1_v1.C_LG_XIX1125	POPTR_0019s05430.1	85.0/6.2	24/3	160.26	Methionine synthase	92%	A6YGE7	*Carica papaya*	no	no
823714	estExt_fgenesh4_pg.C_LG_XIII0289	POPTR_0013s05850.1	85.1/6.1	26/5	180.37	Methionine synthase	91%	A6YGE7	*Carica papaya*	no	no
679841	grail3.0066005802	POPTR_0004s20220.1	84.6/6.3	33/12	270.39	Methionine synthase	90%	A6YGE7	*Carica papaya*	no	yes
821018	estExt_fgenesh4_pg.C_LG_IX0162	POPTR_0009s15490.1	84.6/6.2	32/12	266.35	Methionine synthase	90%	A6YGE7	*Carica papaya*	no	yes
707148	estExt_Genewise1_v1.C_LG_I3343	POPTR_0001s32780.1	53.4/5.6	6/1	40.19	Adenosylhomocysteinase	95%	A9PIA2	*Populus trichocarpa*	no	no
835881	estExt_fgenesh4_pm.C_LG_XVIII0241	POPTR_0018s08910.1	44.5/7.7	4/3	40.26	Aspartate aminotransferase	92%	A7PEM9	*Vitis vinifera*	no	yes
553231	eugene3.00002546	POPTR_0002s25070.1	27.1/8.6	3/3	20.15	Auxin-induced protein 12	41%	Q94BT2	*Arabidopsis thaliana*	yes	
817858	estExt_fgenesh4_pg.C_LG_IV0521	POPTR_0004s04370.1	63.8/6.9	3/1	10.17	Ketol-acid reductoisomerase	86%	Q05758	*Arabidopsis thaliana*	no	yes
828210	estExt_fgenesh4_pg.C_1450022	POPTR_0018s07000.1	53.5/5.8	3/3	20.21	Nucleotide pyrophosphatase-like protein	70%	Q9FS13	*Spinaciao leracea*	signal anchor	yes
816369*	estExt_fgenesh4_pg.C_LG_II0927	POPTR_0002s10150.1	18.8/5.6	3/2	48.26	Blue copper-like protein	55%	A3F8V0	*Gossypum hirsutum*	yes	
174402[#] *	gw1.I.3002.1	POPTR_0001s33960.1	11.9/8.5	2/2	20.23	Blue copper protein	70%	Q8LED5	*Arabidopsis thaliana*	yes	
177387[#]	gw1.I.5987.1	POPTR_0001s46710.1	56.8/6.0	11/4	78.31	At1g30760/T5I8_22	66%	Q93ZA3	*Arabidopsis thaliana*	no	no
**Structural**											
668776	grail3.0007002601	POPTR_0018s04840.1	14.0/4.5	2/1	20.16	Profilin	100%	A9P8K3	*Populus trichocarpa*	no	yes
**Unclassified**											
560298	eugene3.00060257	POPTR_0006s02790.1	81.7/5.6	1/1	8.14	ARE1-like protein	81%	Q68EC6	*Arabidopsis thaliana*	no	no
732264*	estExt_Genewise1_v1.C_LG_XIV3377	POPTR_0014s15330.1	26.2/5.2	5/5	50.32	Carboxymethylenebutenolidase	84%	O80889	*Arabidopsis thaliana*	no	no
216788[#]	gw1.VII.1093.1	POPTR_0421s00220.1	19.2/9.1	4/2	36.16	Cysteine-rich repeat secretory protein 38	56%	Q9LRJ9	*Arabidopsis thaliana*	no	no
814847*	estExt_fgenesh4_pg.C_LG_I0347	POPTR_0001s05560.1	95.3/5.5	26/22	188.43	no sequence similarity to a known protein				yes	
835930	estExt_fgenesh4_pm.C_LG_XVIII0321	POPTR_0018s10920.1	18.4/6.6	2/2	20.26	no sequence similarity to a known protein				yes	

Table S4. continued

249488[#]	gw1.XIX.1888.1	POPTR_0019s10550.1	52.6/6.0	3/3	20.12	no sequence similarity to a known protein	no	yes
413168[#]	gw1.III.271.1	POPTR_0003s20500.1	95.2/6.4	5/1	36.33	no sequence similarity to a known protein	yes	

[#] protein sequence in JGI database is incomplete

* protein was also identified via 2D-PAGE MSMS

SA - protein is predicted to serve as signal anchor
