# Supplementary material for: Apoplast proteome reveals that extracellular matrix contributes to multistress response in poplar
Source: BMC Genomics. 2010 Nov 29;11:674. doi: 10.1186/1471-2164-11-674 (PMC3091788; doi:10.1186/1471-2164-11-674)
Supplement: Additional file 6 — Supplementary Figure S2. Staining of leaf apoplast proteins to identify post-translational modifications. (A) Leaf apoplast proteins stained with ProQ-Emerald 488 (left panel) to detect glycosylated proteins. The same gel was post-stained with SYPRO Ruby (right panel) to visualize all proteins. Boxed spots annotated with letters indicate glycosylated proteins whose IDs are given in additional file 2: Table S1. Protein molecular weights are shown in kilodaltons (kDa). (B) Leaf apoplast proteins stained with ProQ-Diamond (left panel) to detect phosphorylated proteins. The same gel was post-stained with SYPRO Ruby (right panel) to visualize all proteins. Boxed spots annotated with letters indicate phosphorylated proteins. Box a corresponds to cysteine-rich repeat secretory protein 38 (spot 14; POPTR_1698s00200.1; additional file 2: Table S1), whereas box b refers to thaumatin-like protein (POPTR_0018s10490.1; spot 88; additional file 2: Table S1). Protein molecular weights are shown in kilodaltons (kDa). [file 1471-2164-11-674-S6.PDF]

Additional file 6

File format: PDF

Title: Supplementary Figure S2

Description:
Figure S2. Staining of leaf apoplast proteins to identify post-translational modifications. (A) Leaf apoplast proteins stained with ProQ-Emerald 488 (left panel) to detect glycosylated proteins. The same gel was post-stained with SYPRO Ruby (right panel) to visualize all proteins. Boxed spots annotated with letters indicate glycosylated proteins whose IDs are given in additional file 2: Table S1. Protein molecular weights are shown in kilodaltons (kDa). (B) Leaf apoplast proteins stained with ProQ-Diamond (left panel) to detect phosphorylated proteins. The same gel was post-stained with SYPRO Ruby (right panel) to visualize all proteins. Boxed spots annotated with letters indicate phosphorylated proteins. *Box a* corresponds to cysteine-rich repeat secretory protein 38 (spot 14; POPTR_1698s00200.1; additional file 2: Table S1), whereas *box b* refers to thaumatin-like protein (POPTR_0018s10490.1; spot 88; additional file 2: Table S1). Protein molecular weights are shown in kilodaltons (kDa).

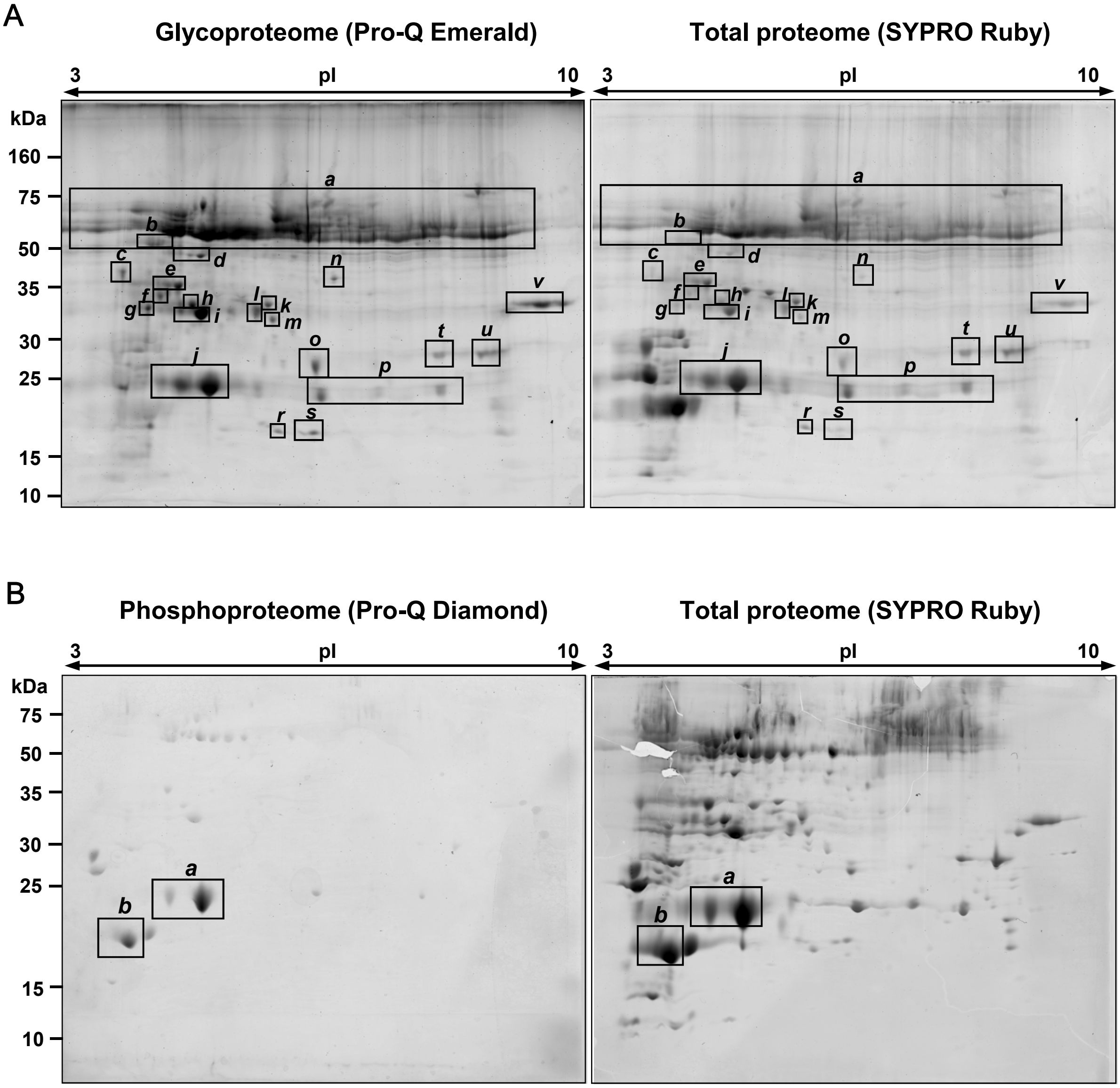
A
Glycoproteome (Pro-Q Emerald)
Total proteome (SYPRO Ruby)
3
pI
10
kDa
160
75
50
35
30
25
15
10
a
b
c
d
e
f
g
h
i
j
k
l
m
n
o
p
r
s
t
u
v
B
Phosphoproteome (Pro-Q Diamond)
Total proteome (SYPRO Ruby)
3
pI
10
kDa
75
50
35
30
25
15
10
a
b
