# Supplementary material for: Apoplast proteome reveals that extracellular matrix contributes to multistress response in poplar
Source: BMC Genomics. 2010 Nov 29;11:674. doi: 10.1186/1471-2164-11-674 (PMC3091788; doi:10.1186/1471-2164-11-674)
Supplement: Additional file 7 — Supplementary Figure S3. Cluster analysis of year-round expression profiles of genes corresponding to 139 leaf apoplast proteins shown in Figure 4A. The log2 fold-change (FC) is shown on the left. Dotted lines (black) denote the expression profiles of individual genes, whereas the solid lines (red) represent the mean expression for the cluster. For each time point, boxplot representation of the expression profile for all filtered array elements is provided. [file 1471-2164-11-674-S7.PDF]

Additional file 7

File format: PDF

Title: Supplementary Figure S3

Description:
Figure S3. Cluster analysis of year-round expression profiles of genes corresponding to 139 leaf apoplast proteins shown in Figure 4A. The log2 fold-change (FC) is shown on the left. Dotted lines (black) denote the expression profiles of individual genes, whereas the solid lines (red) represent the mean expression for the cluster. For each time point, boxplot representation of the expression profile for all filtered array elements is provided.

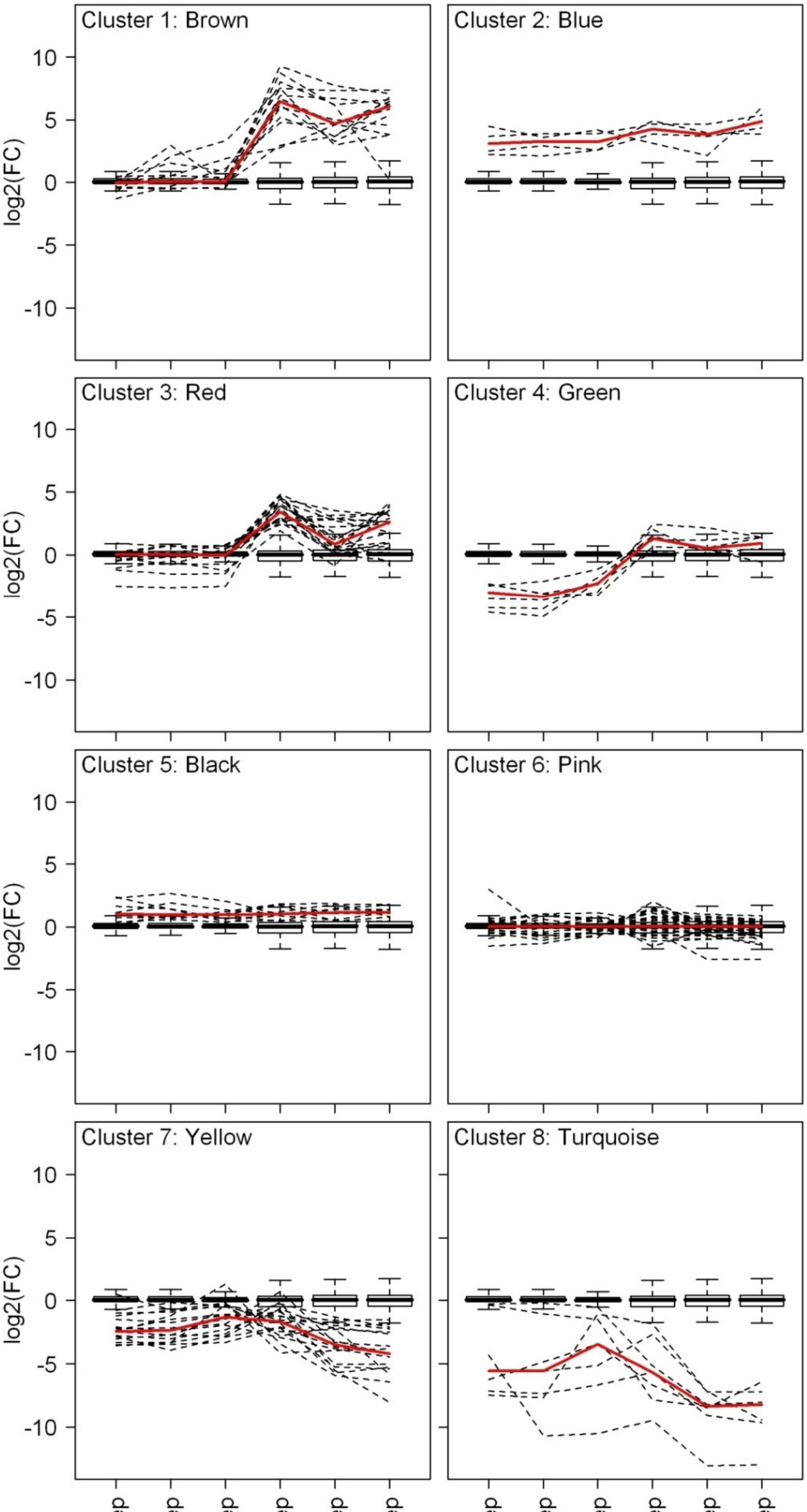
Cluster 1: Brown
Cluster 2: Blue
Cluster 3: Red
Cluster 4: Green
Cluster 5: Black
Cluster 6: Pink
Cluster 7: Yellow
Cluster 8: Turquoise
log2(FC)
10
5
0
-5
-10
DecVSep
FebVSep
MarVSep
AprVSep
MayVSep
JunVSep
