# Supplementary material for: Apoplast proteome reveals that extracellular matrix contributes to multistress response in poplar
Source: BMC Genomics. 2010 Nov 29;11:674. doi: 10.1186/1471-2164-11-674 (PMC3091788; doi:10.1186/1471-2164-11-674)
Supplement: Additional file 8 — Supplementary Figure S4. Validation of microarray data via qRT-PCR using leaves from two other sexually mature P. deltoides trees (Tree 1 and Tree 2), predictive significance (p-value) and relationship strength (R2 value) with microarray intensity values. (A) Alcohol dehydrogenase 2 (POPTR_0002s07290.1): For Tree 1, p = 0.007 and R2 = 0.54; For Tree 2, p = 0.16 and R2 = 0.19. (B) Abscisic acid (ABA) analysis in poplar (P. deltoides) leaves in April, May, June, and July. (C) Cationic peroxidase 1 (POPTR_0016s14030.1): For Tree 1, p = 0.164 and R2 = 0.18; For Tree 2, p = 0.019 and R2 = 0.44. (D) Thaumatin-like protein (POPTR_0018s10490.1): For Tree 1, p < 0.001, R2 = 0.88; For Tree 2, p = 0.003 and R2 = 0.59. (E) Blight-associated p12 (POPTR_0006s19310.1): For Tree 1, p = 0.06, R2 = 0.30, For Tree 2, p = 0.016 and R2 = 0.46. (F) Phenylcoumaran benzylic ether reductase (POPTR_0002s03580.1): For Tree1, p= 0.002, R2 = 0.64; For Tree 2, p = 0.51 and R2 = 0.042. (G) Dehydration stress-induced protein (POPTR_0007s05650.1): For Tree 1, p = 0.001 and R2 = 0.65; For Tree 2, p < 0.001 and R2 = 0.78. (H) Wound-responsive (POPTR_0010s16050.1): For Tree 1, p = 0.04, R2 = 0.35; For Tree 2, p = 0.7 and R2 = 0.014. [file 1471-2164-11-674-S8.PDF]

Additional file 8

File format: PDF

Title: Supplementary Figure S4

Description:
Figure S4. Validation of microarray data via qRT-PCR using leaves from two other sexually mature *P. deltoides* trees (Tree 1 and Tree 2), predictive significance (*p*-value) and relationship strength (R^2 value) with microarray intensity values. (A) Alcohol dehydrogenase 2 (POPTR_0002s07290.1): For Tree 1, p=0.007 and R^2=0.54; For Tree 2, p=0.16 and R^2=0.19. (B) Abscisic acid (ABA) analysis in poplar (*P. deltoides*) leaves in April, May, June, and July. (C) Cationic peroxidase 1 (POPTR_0016s14030.1): For Tree 1, p=0.164 and R^2=0.18; For Tree 2, p=0.019 and R^2=0.44. (D) Thaumatin-like protein (POPTR_0018s10490.1): For Tree 1, p<0.001, R^2=0.88; For Tree 2, p=0.003 and R^2=0.59. (E) Blight-associated p12 (POPTR_0006s19310.1): For Tree 1, p=0.06, R^2=0.30, For Tree 2, p=0.016 and R^2=0.46. (F) Phenylcoumaran benzylic ether reductase (POPTR_0002s03580.1): For Tree1, p= 0.002, R^2=0.64; For Tree 2, p=0.51 and R^2=0.042. (G) Dehydration stress-induced protein (POPTR_0007s05650.1): For Tree 1, p=0.001 and R^2=0.65; For Tree 2, p<0.001 and R^2=0.78. (H) Wound-responsive (POPTR_0010s16050.1): For Tree 1, p=0.04, R^2=0.35; For Tree 2, p=0.7 and R^2=0.014.

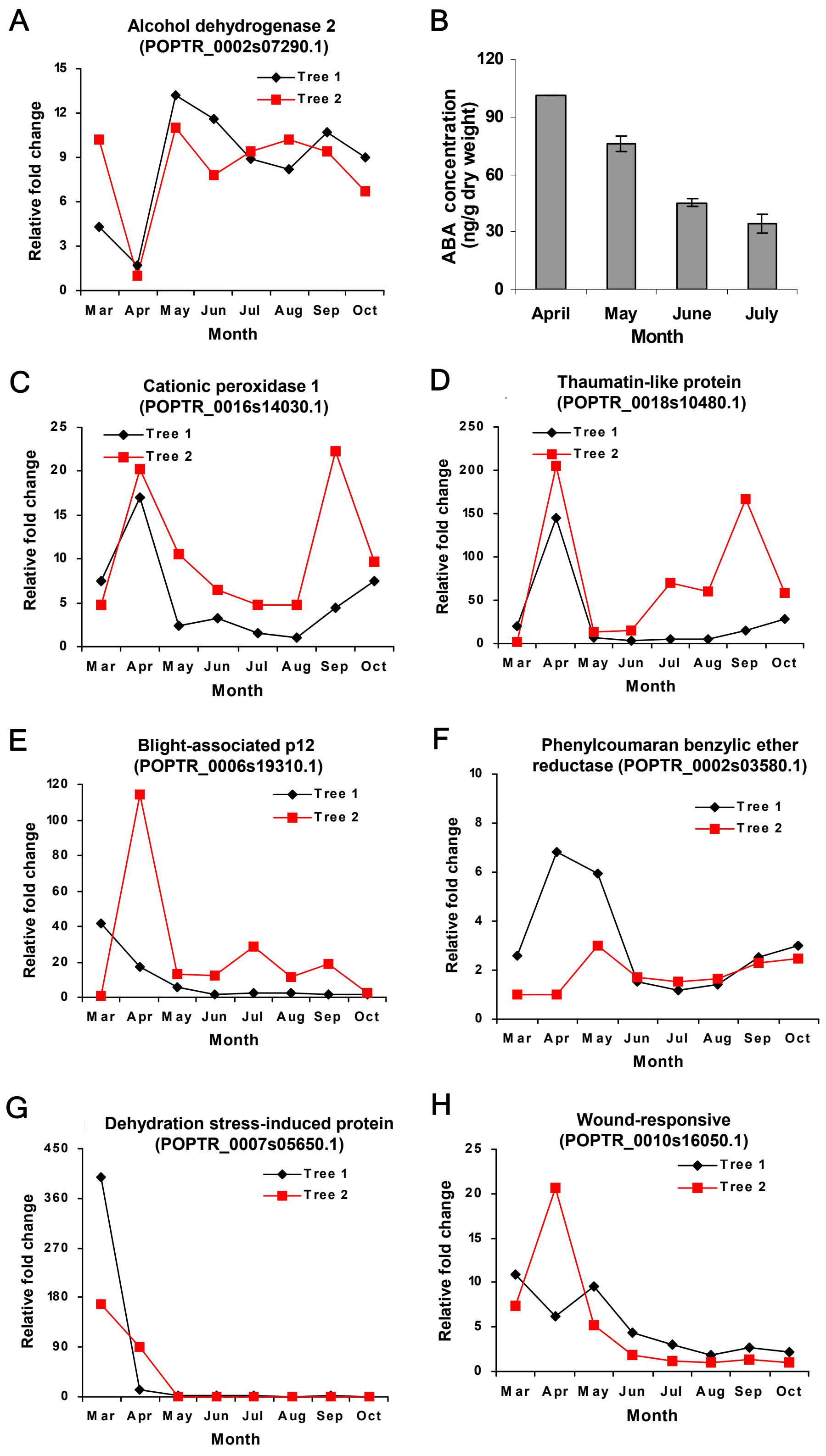
A
Alcohol dehydrogenase 2
(POPTR_0002s07290.1)
Relative fold change
Month
Tree 1
Tree 2
Mar Apr May Jun Jul Aug Sep Oct
B
ABA concentration
(ng/g dry weight)
April May June July
Month
C
Cationic peroxidase 1
(POPTR_0016s14030.1)
D
Thaumatin-like protein
(POPTR_0018s10480.1)
E
Blight-associated p12
(POPTR_0006s19310.1)
F
Phenylcoumaran benzylic ether
reductase (POPTR_0002s03580.1)
G
Dehydration stress-induced protein
(POPTR_0007s05650.1)
H
Wound-responsive
(POPTR_0010s16050.1)
