# Supplementary material for: Apoplast proteome reveals that extracellular matrix contributes to multistress response in poplar
Source: BMC Genomics. 2010 Nov 29;11:674. doi: 10.1186/1471-2164-11-674 (PMC3091788; doi:10.1186/1471-2164-11-674)
Supplement: Additional file 9 — Supplementary Table S5. Clusters of year-round expression profiles of genes corresponding to 139 leaf apoplast proteins and their functional annotation. [file 1471-2164-11-674-S9.PDF]

Additional file 9

File format: PDF

Title: Supplementary Table S5

Description:
Table S5. Clusters of year-round expression profiles of genes corresponding to 139 leaf apoplast proteins and their functional annotation.

Cluster	Colors	Gene ID	Protein ID	Poplar new ID	Annotation
1	turquoise	estExt_fgenesh4_pg.C_LG_VII0502	819822	POPTR_0007s05650.1	Dehydration stress-induced protein
1	turquoise	estExt_fgenesh4_pm.C_LG_VI0650	832078	POPTR_0006s24030.1	Cytosolic class II low molecular weight heat shock protein
1	turquoise	estExt_Genewise1_v1.C_LG_X0701	723969	POPTR_0010s16030.1	Populus x generosa pop3 peptide
1	turquoise	eugene3.01210029	580490	POPTR_0006s18240.1	GDSL-motif lipase/hydrolase-like protein
1	turquoise	grail3.0039009101	644497	POPTR_0002s16390.1	Polygalacturonase-like protein
1	turquoise	gw1.XVIII.3492.1	262951	POPTR_0025s00410.1	Heparanase-like protein 3
1	turquoise	grail3.0064002301	679511	POPTR_0017s06550.2	Peroxidase a
2	blue	estExt_fgenesh4_pg.C_LG_VIII0748	820480	POPTR_0008s08640.1	S-like ribonuclease
2	blue	gw1.IX.21.1	199556	POPTR_0009s00820.1	Serine carboxypeptidase-like 51
2	blue	grail3.0033012902	644125	POPTR_0515s00220.2	Alpha-amylase
2	blue	gw1.40.747.1	286987	POPTR_0014s02940.1	Alpha-amylase
3	brown	estExt_fgenesh4_pg.C_LG_XVI1240	825400	POPTR_0016s14030.1	Cationic peroxidase 1
3	brown	estExt_fgenesh4_pg.C_LG_XVIII0531	825802	POPTR_0018s03020.1	41 kD chloroplast nucleoid DNA binding protein
3	brown	eugene3.00012306	549865	POPTR_0001s31740.1	Tumor-related protein
3	brown	eugene3.00111309	569295	POPTR_0011s15750.1	Alpha-glucosidase 1
3	brown	eugene3.00190854	574380	POPTR_0019s12360.1	Class IV chitinase
3	brown	eugene3.01180035	580100	POPTR_0003s08760.1	Mitochondrial glycine decarboxylase complex H-protein
3	brown	fgenesh4_pg.C_LG_XIV000840	774930	POPTR_0014s14000.1	Peroxidase
3	brown	gw1.XIX.1495.1	249095	POPTR_0019s08160.1	Wound-inducible carboxypeptidase
3	brown	estExt_fgenesh4_pg.C_LG_II0927	816369	POPTR_0002s10150.1	Blue copper-like protein
3	brown	estExt_Genewise1_v1.C_1970084	746640	POPTR_0015s05990.1	Acidic class III chitinase
3	brown	estExt_Genewise1_v1.C_4720001	747341	POPTR_0001s09000.1	Thaumatin-like protein isoform 2
3	brown	gw1.5405.1.1	290846	POPTR_0001s26210.1	Beta-1,3-glucanase
3	brown	gw1.IX.3630.1	203165	POPTR_0019s10310.1	Beta-1,3-glucanase
3	brown	gw1.VI.1875.1	417502	POPTR_0006s24850.1	Nucleoid DNA-binding protein cnd41-like protein
4	yellow	estExt_fgenesh4_pg.C_LG_II2363	816882	POPTR_0002s23920.1	Alpha-mannosidase
4	yellow	estExt_fgenesh4_pg.C_LG_XVI0047	824970	POPTR_0016s01210.1	Membrane protein
4	yellow	estExt_Genewise1_v1.C_LG_VII1054	718485	POPTR_0007s02580.1	Peroxidase
4	yellow	estExt_Genewise1_v1.C_LG_VII1401	718566	POPTR_0007s04020.1	Pectin methylesterase-like protein
4	yellow	eugene3.00002546	553231	POPTR_0002s25070.1	Auxin-induced protein 12
4	yellow	eugene3.00151093	575698	POPTR_0015s14380.1	Enolase
4	yellow	eugene3.00700152	595511	POPTR_0005s04590.1	Superoxide dismutase [Cu-Zn]
4	yellow	fgenesh4_pg.C_LG_II001910	755730	POPTR_0002s18800.1	Polygalacturonase
4	yellow	fgenesh4_pm.C_LG_I000205	797202	POPTR_0001s13140.1	Serine carboxypeptidase-like 45
4	yellow	fgenesh4_pm.C_LG_IV000380	800693	POPTR_0004s14240.1	Peroxidase ATP17a like protein
4	yellow	grail3.0011011501	654740	POPTR_0007s05100.1	Peroxidase 17
4	yellow	grail3.0045003902	648596	POPTR_0004s16120.2	Glucan endo-1,3-beta-glucosidase 7

Table S5. continued

4	yellow	gw1.40.953.1	287193	POPTR_0014s02270.1	Probable carbohydrate esterase At4g34215
4	yellow	gw1.I.26.1	171426	POPTR_0001s21430.1	Xylan 1,4-beta-xylosidase
4	yellow	gw1.V.4245.1	208844	POPTR_0005s18880.1	Subtilisin-like protease
4	yellow	gw1.XV.3396.1	253960	POPTR_0015s12990.1	Pectinacetylesterase
4	yellow	eugene3.68730001	595225	POPTR_0003s13160.1	Polygalacturonase-like protein
4	yellow	gw1.III.413.1	413310	POPTR_0003s13160.1	Polygalacturonase-like protein
5	green	estExt_fgenesh4_kg.C_LG_II0003	813630	POPTR_0002s01740.1	Plastocyanin B
5	green	estExt_fgenesh4_pg.C_860139	827492	POPTR_0004s24220.4	Pectinacetylesterase precursor
5	green	estExt_fgenesh4_pm.C_LG_II0164	830063	POPTR_0002s03580.1	Phenylcoumaran benzylic ether reductase
5	green	eugene3.00012396	549955	POPTR_0001s30680.1	NtPRp27
5	green	eugene3.00021116	551801	POPTR_0002s12130.1	Serine protease
5	green	grail3.0002074001	652151	POPTR_0005s26740.1	Plastocyanin A
6	red	estExt_fgenesh4_pg.C_1970027	828660	POPTR_0015s05980.1	Hevamine-A
6	red	estExt_fgenesh4_pg.C_LG_I0347	814847	POPTR_0001s05560.1	no sequence similarity to a known protein
6	red	estExt_fgenesh4_pg.C_LG_VI1270	819386	POPTR_0006s19310.1	Blight-associated protein p12
6	red	estExt_fgenesh4_pm.C_400050	836412	POPTR_0014s04360.1	Sts15 protein
6	red	estExt_Genewise1_v1.C_400832	740448	POPTR_0014s02630.1	Subtilase
6	red	eugene3.00121097	570444	POPTR_0012s13090.1	Pectinacetylesterase
6	red	fgenesh4_pg.C_LG_II001639	755459	POPTR_0002s17860.1	3-ketoacyl-CoA synthase 10
6	red	fgenesh4_pg.C_scaffold_164000005	786149	POPTR_0018s14920.1	Alpha-galactosidase
6	red	fgenesh4_pg.C_scaffold_40000309	781559	POPTR_0014s02650.1	Subtilase
6	red	fgenesh4_pm.C_LG_VII000423	802937	POPTR_0007s13010.2	Bg55 protein
6	red	grail3.0001063901	649635	POPTR_0009s09760.1	NtPRp27
6	red	grail3.0005000301	666348	POPTR_0015s07660.1	(-)-isopiperitenol dehydrogenase
6	red	grail3.0020019002	669475	POPTR_0018s10480.1	Thaumatin-like protein
6	red	grail3.0020020701	669494	POPTR_0018s10730.1	Blight-associated protein p12
6	red	grail3.0028002001	675847	POPTR_0006s14400.1	Cysteine protease CP1
6	red	estExt_Genewise1_v1.C_LG_VII1106	718495	POPTR_1698s00200.1	Cysteine-rich repeat secretory protein 38
6	red	eugene3.00070258	562320	POPTR_1698s00200.1	Cysteine-rich repeat secretory protein 38
6	red	fgenesh4_pg.C_LG_I001216	751613	POPTR_0001s22090.1	Prolylcarboxypeptidase-like protein
6	red	gw1.10474.1.1	263224	POPTR_0019s08160.1	Serine carboxypeptidase-like 20
6	red	gw1.IX.2413.1	201948	POPTR_0009s09750.1	NtPRp27-like protein
6	red	gw1.XI.1958.1	233978	POPTR_0012s01160.1	Pathogenesis-related protein 8
7	black	estExt_fgenesh4_pg.C_LG_II0662	816232	POPTR_0002s07290.1	Alcohol dehydrogenase 2
7	black	estExt_fgenesh4_pm.C_LG_VIII0763	832859	POPTR_0008s18160.1	F21J9.10
7	black	estExt_Genewise1_v1.C_LG_XVI1825	735049	POPTR_0016s05800.1	Beta-1,3 glucanase
7	black	eugene3.00060326	560367	POPTR_0006s03490.1	Serine carboxypeptidase family protein
7	black	eugene3.00131211	571847	POPTR_0013s14800.1	Cell survival CED-4-interacting protein MAC-1
7	black	gw1.III.1797.1	414694	POPTR_0003s17460.1	Purple acid phosphatase
7	black	gw1.IX.4803.1	204338	POPTR_0009s01780.1	Beta-galactosidase
7	black	gw1.IX.4973.1	204508	POPTR_0009s01240.1	Alpha-L-fucosidase 1 precursor

Table S5. continued

7	black	gw1.X.619.1	225922	POPTR_0010s15510.1	O-linked GlcNAc transferase
8	pink	estExt_fgenesh4_pg.C_1210040	827727	POPTR_0006s18230.1	Proline-rich protein
8	pink	estExt_fgenesh4_pg.C_1450022	828210	POPTR_0018s07000.1	Nucleotide pyrophosphatase-like protein
8	pink	estExt_fgenesh4_pg.C_LG_III1873	817694	POPTR_0003s21660.1	Peroxidase N
8	pink	estExt_fgenesh4_pg.C_LG_X0820	821987	POPTR_0010s10020.1	F9D12.16 protein (Copper binding protein-like, predicted GPI-anchored protein)
8	pink	estExt_fgenesh4_pg.C_LG_X1353	822230	POPTR_0010s16050.1	Wound-responsive
8	pink	estExt_fgenesh4_pg.C_LG_XVI0953	825296	POPTR_0016s10140.1	Protease inhibitor/seed storage/lipid transfer protein (LTP) family protein
8	pink	estExt_fgenesh4_pm.C_LG_XIV0520	835003	POPTR_0014s17580.1	Serine carboxypeptidase-like 20
8	pink	estExt_Genewise1_v1.C_LG_II0461	709916	POPTR_0002s02010.1	Subtilisin-like serine protease
8	pink	eugene3.00021016	551701	POPTR_0002s11090.1	LG27/30-like gene (rhamnogalacturonate lyase)
8	pink	eugene3.00030575	553942	POPTR_0007s10160.1	Tetratricopeptide repeat (TPR)-containing protein
8	pink	eugene3.00101510	566952	POPTR_0010s16790.1	Purple acid phosphatase
8	pink	eugene3.00111328	569314	POPTR_0011s15950.1	FAD linked oxidase, N-terminal
8	pink	eugene3.00140379	572334	POPTR_0014s08860.1	Class III chitinase
8	pink	eugene3.00140904	572859	POPTR_0014s14050.1	Alpha-mannosidase
8	pink	eugene3.00190700	574226	POPTR_0019s10720.1	Leucine-rich receptor-like protein kinase
8	pink	eugene3.01970027	586585	POPTR_0015s06000.1	Acidic class III chitinase
8	pink	eugene3.10400001	579227	POPTR_0185s00200.1	Resistance protein RGC2
8	pink	eugene3.26820002	588847		no sequence similarity to a known protein
8	pink	fgenesh4_pg.C_LG_III000676	757134	POPTR_0007s09730.1	Alpha-mannosidase
8	pink	fgenesh4_pg.C_LG_VI000192	761882	POPTR_0006s02260.1	Abrin-a
8	pink	fgenesh4_pg.C_LG_XI001367	772214	POPTR_0011s16170.1	Carbohydrate oxidase
8	pink	fgenesh4_pg.C_scaffold_3039000001	791899		no sequence similarity to a known protein
8	pink	fgenesh4_pg.C_scaffold_40000333	781583	POPTR_0014s02410.1	Cysteine protease CP1
8	pink	fgenesh4_pg.C_scaffold_44000051	781921	POPTR_0003s02750.1	Pathogenesis-related transcriptional factor
8	pink	fgenesh4_pg.C_scaffold_70000003	782655	POPTR_0005s06140.1	Alcohol dehydrogenase
8	pink	fgenesh4_pm.C_LG_II000364	798675	POPTR_0002s08120.1	Beta-galactosidase 16
8	pink	grail3.0008013701	640081	POPTR_0001s13320.1	Leucine-rich repeat protein
8	pink	grail3.0016022201	662785	POPTR_0013s02730.2	Lipolytic enzyme, G-D-S-L
8	pink	grail3.0024007102	652386	POPTR_0006s01080.1	Fumarylacetoacetase
8	pink	grail3.0024032801	652688	POPTR_0006s04670.1	Beta-1,3 glucanase
8	pink	grail3.0038010001	648132	POPTR_0004s08500.1	Non-specific lipid-transfer protein
8	pink	gw1.142.209.1	270686	POPTR_0013s12870.1	Class IV chitinase
8	pink	gw1.152.150.1	272681	POPTR_0006s06100.1	Heparanase-like protein 3
8	pink	gw1.40.264.1	286504	POPTR_0014s04850.1	Cu2+ plastocyanin-like
8	pink	gw1.66.413.1	293740	POPTR_0004s20970.1	AT3g11590/F24K9_26
8	pink	gw1.66.741.1	294068	POPTR_0004s21990.1	E3 SUMO-protein ligase SIZ1
8	pink	gw1.6840.1.1	294386	POPTR_0001s09570.1	Osmotin-like protein linusitin
8	pink	gw1.88.36.1	297914	POPTR_0017s08470.1	Beta-galactosidase
8	pink	gw1.I.5987.1	177387	POPTR_0001s46710.1	At1g30760/T5I8_22
8	pink	gw1.I.6683.1	178083	POPTR_0003s21660.1	Peroxidase 59

Table S5. continued

8	pink	gw1.II.1445.1	410110	POPTR_0002s13970.1	Nucleoside diphosphate kinase
8	pink	gw1.IV.3557.1	198468	POPTR_0004s08710.1	Disease resistance protein
8	pink	gw1.IX.5004.1	204539	POPTR_0009s01140.1	DNA ligase
8	pink	gw1.V.2620.1	207219	POPTR_0005s07020.1	Beta-galactosidase
8	pink	gw1.V.3892.1	208491	POPTR_0005s14190.1	Peroxidase 10 precursor
8	pink	gw1.V.5003.1	209602	POPTR_0005s16590.1	Putative glucan 1,3-beta-glucosidase
8	pink	gw1.V.5394.1	209993	POPTR_0005s20280.1	Beta-galactosidase 16
8	pink	gw1.VI.1783.1	417410	POPTR_0006s06650.1	Dicyanin
8	pink	gw1.VI.2836.1	418463	POPTR_0006s01000.1	ABC transporter
8	pink	gw1.VIII.806.1	419378	POPTR_0008s15040.1	F9D12.16 protein (Copper binding protein-like, predicted GPI-anchored protein)
8	pink	gw1.X.3030.1	228333	POPTR_0010s26170.1	Acid alpha galactosidase 1
8	pink	gw1.XII.1318.1	422858	POPTR_0012s10830.1	Alpha-mannosidase
8	pink	gw1.XV.2023.1	252587	POPTR_0015s10470.1	Auxin-independent growth promoter-like protein
8	pink	gw1.XVI.1041.1	255102	POPTR_0016s02620.2	Alpha-L-arabinofuranosidase
8	pink	gw1.XVI.799.1	254860	POPTR_0016s01920.1	Pectinesterase
8	pink	gw1.XVIII.493.1	259952	POPTR_0018s14060.1	Salt-inducible protein
8	pink	fgenesh4_pg.C_LG_XVI000455	777213	POPTR_0149s00200.1	Peroxidase
8	pink	fgenesh4_pm.C_LG_III000026	799561	POPTR_0003s01730.1	Xylan 1,4-beta-xylosidase
8	pink	fgenesh4_pm.C_scaffold_163000009	811643	POPTR_0011s01280.1	Superoxide dismutase [Cu-Zn]
8	pink	gw1.VII.1093.1	216788	POPTR_0421s00220.1	Cysteine-rich repeat secretory protein 38
