# Supplementary material for: Apoplast proteome reveals that extracellular matrix contributes to multistress response in poplar
Source: BMC Genomics. 2010 Nov 29;11:674. doi: 10.1186/1471-2164-11-674 (PMC3091788; doi:10.1186/1471-2164-11-674)
Supplement: Additional file 10 — Supplementary Figure S5. Phylogenetic analysis of alcohol dehydrogenase proteins in poplar (POPTR) and Arabidopsis thaliana (At). Poplar leaf (POPTR_0005s06140.1 and POPTR_0002s07290.1 in purple) and root (POPTR_0008s16150.1 in red) apoplast alcohol dehydrogenases fall into different clades. [file 1471-2164-11-674-S10.PDF]

Additional file 10

File format: PDF

Title: Supplementary Figure S5

Description:
Figure S5. Phylogenetic analysis of alcohol dehydrogenase proteins in poplar (POPTR) and *Arabidopsis thaliana* (At). Poplar leaf (POPTR_0005s06140.1 and POPTR_0002s07290.1 in purple) and root (POPTR_0008s16150.1 in red) apoplast alcohol dehydrogenases fall into different clades.

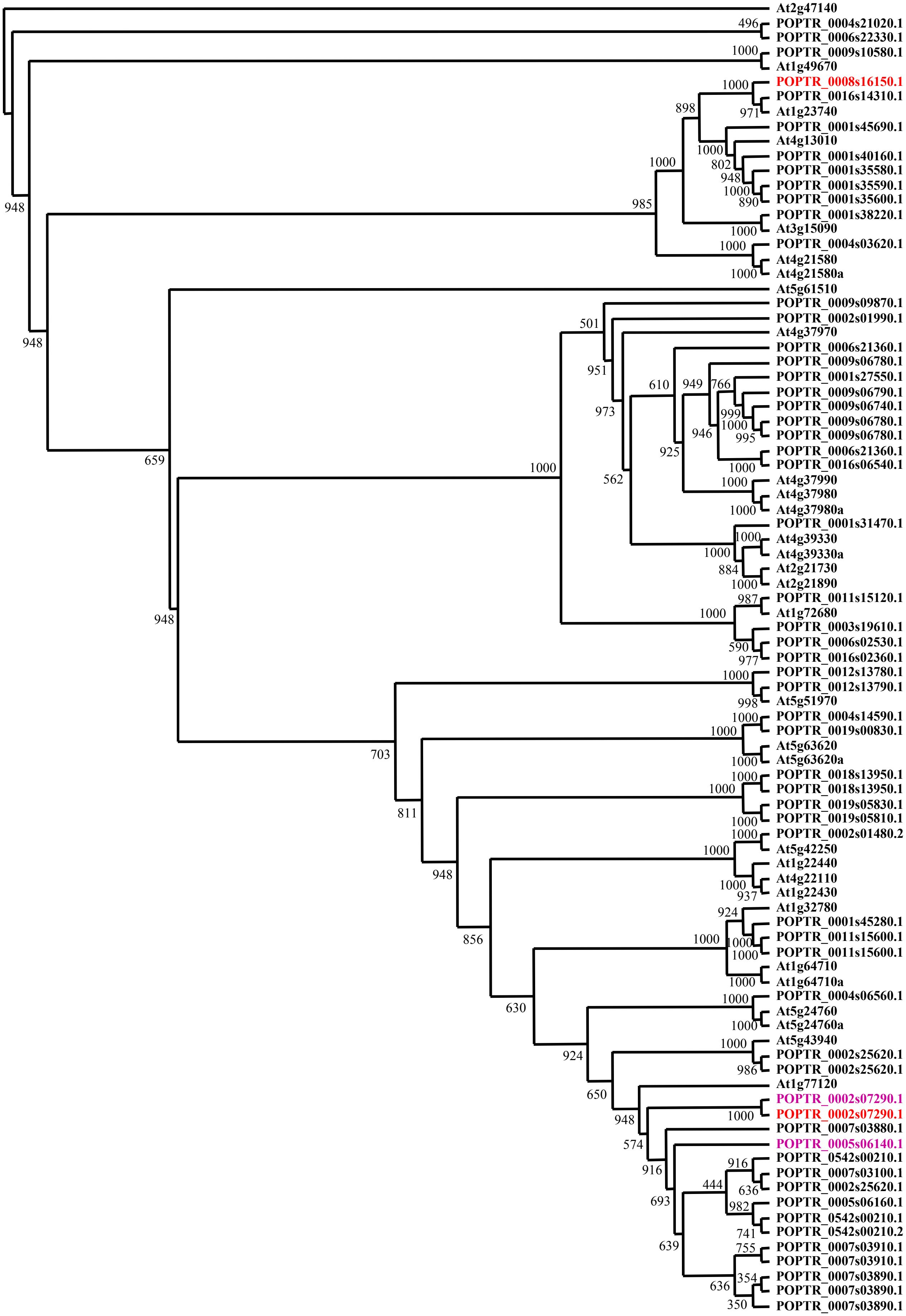
At2g47140
POPTR_0004s21020.1
POPTR_0006s22330.1
POPTR_0009s10580.1
At1g49670
POPTR_0008s16150.1
POPTR_0016s14310.1
At1g23740
POPTR_0001s45690.1
At4g13010
POPTR_0001s40160.1
POPTR_0001s35580.1
POPTR_0001s35590.1
POPTR_0001s35600.1
POPTR_0001s38220.1
At3g15090
POPTR_0004s03620.1
At4g21580
At4g21580a
At5g61510
POPTR_0009s09870.1
POPTR_0002s01990.1
At4g37970
POPTR_0006s21360.1
POPTR_0009s06780.1
POPTR_0001s27550.1
POPTR_0009s06790.1
POPTR_0009s06740.1
POPTR_0009s06780.1
POPTR_0009s06780.1
POPTR_0006s21360.1
POPTR_0016s06540.1
At4g37990
At4g37980
At4g37980a
POPTR_0001s31470.1
At4g39330
At4g39330a
At2g21730
At2g21890
POPTR_0011s15120.1
At1g72680
POPTR_0003s19610.1
POPTR_0006s02530.1
POPTR_0016s02360.1
POPTR_0012s13780.1
POPTR_0012s13790.1
At5g51970
POPTR_0004s14590.1
POPTR_0019s00830.1
At5g63620
At5g63620a
POPTR_0018s13950.1
POPTR_0018s13950.1
POPTR_0019s05830.1
POPTR_0019s05810.1
POPTR_0002s01480.2
At5g42250
At1g22440
At4g22110
At1g22430
At1g32780
POPTR_0001s45280.1
POPTR_0011s15600.1
POPTR_0011s15600.1
At1g64710
At1g64710a
POPTR_0004s06560.1
At5g24760
At5g24760a
At5g43940
POPTR_0002s25620.1
POPTR_0002s25620.1
At1g77120
POPTR_0002s07290.1
POPTR_0002s07290.1
POPTR_0007s03880.1
POPTR_0005s06140.1
POPTR_0542s00210.1
POPTR_0007s03100.1
POPTR_0002s25620.1
POPTR_0005s06160.1
POPTR_0542s00210.1
POPTR_0542s00210.2
POPTR_0007s03910.1
POPTR_0007s03910.1
POPTR_0007s03890.1
POPTR_0007s03890.1
POPTR_0007s03890.1
