# Supplementary material for: Apoplast proteome reveals that extracellular matrix contributes to multistress response in poplar
Source: BMC Genomics. 2010 Nov 29;11:674. doi: 10.1186/1471-2164-11-674 (PMC3091788; doi:10.1186/1471-2164-11-674)
Supplement: Additional file 11 — Supplementary Table S6. Modeling leaf, shoot, and root apoplast proteomics data into regulatory networks and pathways via Pathway Studio using prior knowledge. This pathway contains treatments, primary proteins, effect of primary proteins, and secondary effect leading back to another protein or small molecule. Proteins are referenced by their protein functional name/abbreviation followed by poplar ID in parenthesis (old, new). Effect type is indicated by --+>, ---|, or --->, meaning a strong positive, strong negative, or unresolved association, respectively. Abbreviations: SCPL20 = serine carboxypeptidase-like 20, PR5 = pathogenesis-related gene 5, KCS10 = 3-ketoacyl-CoA synthase 10, SIZ1 = E3 SUMO-protein ligase SIZ1, CP1 = cysteine-type peptidase, SOD = superoxide dismutase, PGK = phosphoglycerate kinase, GST = glutathione S-transferase, SAM = S-adenosylmethionine synthetase, CH-IV = Class IV chitinase, TPI = triose-phosphate isomerase, DHAR = dehydroascorbate reductase, BGL2 = beta-1,3-glucanase 2, TIM = triosephosphate isomerase, GAD = glutamate decarboxylase, and ICDH = isocitrate dehydrogenase. [file 1471-2164-11-674-S11.PDF]

Additional file 11

File format: PDF

Title: Supplementary Table S6

Description:
Table S6. Modeling leaf, shoot, and root apoplast proteomics data into regulatory networks and pathways via Pathway Studio using prior knowledge. This pathway contains treatments, primary proteins, effect of primary proteins, and secondary effect leading back to another protein or small molecule. Proteins are referenced by their protein functional name/abbreviation followed by poplar ID in parenthesis (old, new). Effect type are indicated by --+>, ---|, or ---> , meaning a strong positive, strong negative, or unresolved association, respectively. Abbreviations: SCPL20=serine carboxypeptidase-like 20, PR5=pathogenesis-related gene 5, KCS10=3-ketoacyl-CoA synthase 10, SIZ1=E3 SUMO-protein ligase SIZ1, CP1=cysteine-type peptidase, SOD=superoxide dismutase, PGK=phosphoglycerate kinase, GST=glutathione S-transferase, SAM=S-adenosylmethionine synthetase, CH-IV=Class IV chitinase, TPI=triose-phosphate isomerase, DHAR=dehydroascorbate reductase, BGL2=beta-1,3-glucanase 2, TIM= triosephosphate isomerase, GAD=glutamate decarboxylase, and ICDH=isocitrate dehydrogenase.

Treatment	Primary Protein	Effect of Primary Protein	Secondary Effect
	Actin<br>(813612,<br>POPTR_0001s31700.1)	--+> cell survival<br>---> hypersensitive response	
pathogen ---| drought --+><br>abscisic acid --+><br>ethylene --+>	Ascorbate peroxidase<br>(821619,<br>POPTR_0009s02070.1)	---| senescence<br>---> hydrogen peroxide<br>---> necrosis<br>---> pathogenesis<br>---> reactive oxygen species	hydrogen peroxide---> Peroxidase (718485)
pathogen --+><br>abscisic acid ---| auxins ---><br>ethylene --+>	BGL2<br>(769807,<br>POPTR_0010s15270.1)	--+> dormancy<br>---> disease resistance<br>---> hydrogen peroxide<br>---> jasmonic acid<br>---> necrosis<br>---> oxidative stress<br>---> reactive oxygen species	hydrogen peroxide ---> Ascorbate peroxidase (821619)<br>hydrogen peroxide ---> Peroxidase (718485)
	CH-IV<br>(270686,<br>POPTR_0013s12870.1)	---> defense	
	CP1<br>(753865,<br>POPTR_0002s00720.1)	--+> senescence	
pathogen ---|	DHAR1<br>(833836,<br>POPTR_0010s21840.1)		
	Enolase<br>(836259,<br>POPTR_0006s11800.1)	--+> programmed cell death	
	GAD<br>(648236,<br>POPTR_0004s07350.1)	--+> oxidative stress<br>---> cell survival<br>---> senescence<br>---> defense<br>---> oxidative stress<br>---> reactive oxygen species	reactive oxygen species --+> GST (243514)<br>reactive oxygen species --+> Peroxidase (718485)<br>reactive oxygen species ---> Ascorbate peroxidase (821619)<br>reactive oxygen species ---> PR5 (294386)
auxin --+>	GST<br>(243514,<br>POPTR_0483s00220.1)	---> disease resistance<br>---> oxidative stress<br>---> reactive oxygen species	reactive oxygen species --+> GST (243514)<br>reactive oxygen species --+> Peroxidase (718485)<br>reactive oxygen species ---> Ascorbate peroxidase (821619)<br>reactive oxygen species ---> PR5 (294386)
	ICDH<br>(678577,<br>POPTR_0017s01410.1)	---> defense<br>---> oxidative stress<br>---> senescence	
auxin --+>	KCS10<br>(755459,<br>POPTR_0014s10010.1)		
	Mangrin<br>(198984,<br>POPTR_0004s10240.1)	---> senescence<br>--->jasmonic Acid	jasmonic acid --+> DHAR1 (833836)<br>jasmonic acid --+> Peroxidase (718485)<br>jasmonic acid ---> PR5 (294386)<br>jasmonic acid ---> TPI (724697)
pathogen --+>	Peroxidase<br>(555257,<br>POPTR_0003s21610.1)		

Table S6. continued

drought --+><br>pathogen --+><br>auxin --+><br>abscisic acid --+>	Peroxidase (718485, POPTR_0007s02580.1)	---> lipid peroxides<br>---> necrosis<br>---> disease resistance<br>---> reactive oxygen species<br>---> auxin<br>---> hydrogen peroxide	reactive oxygen species --+> GST (243514)<br>reactive oxygen species --+> Peroxidase (718485)<br>reactive oxygen species ---> Ascorbate peroxidase (821619)<br>reactive oxygen species ---> PR5 (294386)<br>auxin --+> KCS10 (755459)<br>hydrogen peroxide ---> Ascorbate peroxidase (821619)
drought ---|	PGK (564181, POPTR_0008s08400.1)		
pathogen --+><br>drought --+><br>ethylene --->	PR5 (294386, POPTR_0001s09570.1)	--+> necrosis<br>---> disease resistance<br>---> ethylene<br>---> hydrogen peroxide<br>---> jasmonic acid<br>---> oxidative stress	hydrogen peroxide ---> Ascorbate peroxidase (821619)<br>hydrogen peroxide ---> Peroxidase (718485)
	Peroxidase (413562, POPTR_0003s21640.1)	---> lignin concentration	
	SAM (644907, POPTR_0002s19000.1)	---> disease resistance<br>---> senescence	
	SCPL20 (835003, POPTR_0014s17580.1)	---> disease resistance	
	SIZ1 (294068, POPTR_0004s21990.1)	---> necrosis<br>--->disease resistance	
	SOD [Cu-Zn] (595511, POPTR_0005s04590.1)	---> oxidative stress	
abscisic acid --->	Sucrose synthase (835735, POPTR_0018s07380.1)	---> cellulose concentration	
	TIM (713199, POPTR_0004s17530.1)	--+> necrosis	
	TPI (724697, POPTR_0010s21100.1)	---> disease resistance	
