# Supplementary material for: Apoplast proteome reveals that extracellular matrix contributes to multistress response in poplar
Source: BMC Genomics. 2010 Nov 29;11:674. doi: 10.1186/1471-2164-11-674 (PMC3091788; doi:10.1186/1471-2164-11-674)
Supplement: Additional file 12 — Supplementary Table S7. List of poplar apoplast proteins and corresponding transcripts and their expression levels in publicly available poplar (Populus spp.) microarray data obtained from stressed poplar tissues [59,60,141,142]. Dpi, Days Post Infection; Mlp, Melampsora larici-populina; Mmd, Melampsora medusae f. sp. deltoidae. [file 1471-2164-11-674-S12.PDF]

Additional file 12

File format: PDF

Title: Supplementary Table S7

Description:
Table S7. List of poplar apoplast proteins and corresponding transcripts and their expression levels in publicly available poplar (*Populus* spp.) microarray data obtained from stressed poplar tissues [59-60, 141-142]. Dpi, Days Post Infection; Mlp, *Melampsora larici-populina*; Mmd, *Melampsora medusae* f. sp. *deltoidae*.

			Street et al. 2006		Rinaldi et al. 2007			Miranda et al. 2007				Azaiez et al. 2009		
			EST microarray (fold change)		infected with Mlp at 48 hpi			EST microarray (fold change)				Affymetrix microarray (fold change)		
Poplar	Poplar		14 days of drought treatment		NimbleGen	PICME cDNA	cDNA	infected with Mmd with time course				*Melampsora* strains		
protein ID	transcript ID	Annotation	*P. deltoides*	*P. trichocarpa*	oligoarray	microarray	macroarray	1 dpi	3 dpi	6 dpi	9 dpi	Mlp	Mmd	Mix
**Stress/defense**														
233978	gw1.XI.1958.1	Pathogenesis-related protein 8 (POPTR_0012s01160.1)										3.39	2.85	8.83
256724	gw1.XVI.2663.1	Cytosolic ascorbate peroxidase (POPTR_0016s08580.1)			2.6		3.9							
290846	gw1.5405.1.1	Beta-1,3-glucanase (POPTR_0001s26210.1)						1.95	3.88	4.46	6.63			
574380	eugene3.00190854	Class IV chitinase (POPTR_0019s12360.1)										2.43	5.5	7.72
652688	grail3.0024032801	Beta-1,3-glucanase (POPTR_0006s04670.1)										6.46	9.13	16.48
669475	grail3.0020019002	Thaumatin-like protein (POPTR_0018s10480.1)			3.9	28.8	23.3							
669494	grail3.0020020701	Blight-associated protein p12 (POPTR_0018s10730.1)	1.8	1.1	4.1									
726993	estExt_Genewise1_v1.C_LG_XI0270	Benzoquinone reductase (POPTR_0011s13310.1)			3.1	3.1								
727757	estExt_Genewise1_v1.C_LG_XI2337	Benzoquinone reductase (POPTR_0011s03570.1)			3.5	4.4	5.3							
729723	estExt_Genewise1_v1.C_LG_XIII1233	Superoxide dismutase [Cu-Zn] (POPTR_0013s03160.1)			2.2		4.8							
746640	estExt_Genewise1_v1.C_1970084	Acidic class III chitinase (POPTR_0015s05990.1)			6.2	14.4								
811643	fgenesh4_pm.C_scaffold_163000009	Superoxide dismutase [Cu-Zn] (POPTR_0011s01280.1)			3.3									
819386	estExt_fgenesh4_pg.C_LG_VI1270	Blight-associated protein p12 (POPTR_0006s19310.1)							3.77			14.87	6.74	20.96
825296	estExt_fgenesh4_pg.C_LG_XVI0953	Protease inhibitor/seed storage/ lipid transfer protein (LTP) family protein (POPTR_0016s10140.1)				-12.5								

Table S7. continued

**Cell wall metabolism**												
255102	gw1.XVI.1041.1	Alpha-L-arabinofuranosidase (POPTR_0016s02620.2)				3.04	1.71	3.07	3.94			
648596	grail3.0045003902	Glucan endo-1,3-beta-glucosidase 7 (POPTR_0004s16120.2)								5.84	9.19	13.74
816882	estExt_fgenesh4_pg.C_LG_II2363	Alpha-mannosidase (POPTR_0002s23920.1)		3.7								
**Carbohydrate metabolism**												
564942	eugene3.00081537	Malate dehydrogenase (POPTR_0008s16670.1)			2.69							
575307	eugene3.00150702	Glyceraldehyde-3-phosphate dehydrogenase (POPTR_0015s10330.2)	2.19	-2.83								
575698	eugene3.00151093	Enolase (POPTR_0015s14380.1)	2.6		3.5							
593790	eugene3.00570034	Aconitate hydratase 1 (POPTR_0005s10990.1)		2.13								
659332	grail3.0154005402	Phosphoglycerate kinase (POPTR_0010s17870.1)	2.07									
728998	estExt_Genewise1_v1.C_LG_XII1463	Glyceraldehyde-3-phosphate dehydrogenase (POPTR_0012s09570.1)	2.66		2.08							
739764	estExt_Genewise1_v1.C_281127	2,3-bisphosphoglycerate-independent phosphoglycerate mutase (POPTR_0006s11400.1)	2.41									
747123	estExt_Genewise1_v1.C_2730019	Malate dehydrogenase (POPTR_0010s08180.1)	2.48		2.74							
821843	estExt_fgenesh4_pg.C_LG_X0484	Glyceraldehyde-3-phosphate dehydrogenase (POPTR_0010s06560.1)	2.54									
**Cell wall and stress related**												
829298	estExt_fgenesh4_pm.C_LG_I0036	Peroxidase (POPTR_0001s04850.1)	2.7	5.1								
**Unclassified**												
549865	eugene3.00012306	Tumor-related protein (POPTR_0001s31740.1)								2.7	2.23	4.41

59. Rinaldi C, Kohler A, Frey P, Duchaussoy F, Ningre N, Couloux A, Wincker P, Thiec DL, Fluch S, Martin F *et al*: **Transcript profiling of poplar leaves upon infection with compatible and incompatible strains of the foliar rust *Melampsora larici-populina*.** *Plant Physiol* 2007, **144**:347-366.

60. Azaiez A, Boyle B, Levée V, A. S: **Transcriptome profiling in hybrid poplar following interactions with *Melampsora* rust fungi.** *Mol Plant-Microbe Interact* 2009, **22**:190-200.

141. Miranda M, Ralph SG, Mellway R, White R, Heath MC, Bohlmann J, Constabel CP: **The transcriptional response of hybrid poplar (*Populus trichocarpa* x *P. deltoides*) to infection by *Melampsora medusae* leaf rust involves induction of flavonoid pathway genes leading to the accumulation of proanthocyanidins.** *Mol Plant-Microbe Interact* 2007, **20**:816-831.

142. Street NR, Skogstrom O, Sjodin A, Tucker J, Rodrıguez-Acosta R, Nilsson P, Jansson S, Taylor G: **The genetics and genomics of the drought response in *Populus*.** *Plant J* 2006, **48**:321-341.
