# Supplementary material for: Apoplast proteome reveals that extracellular matrix contributes to multistress response in poplar
Source: BMC Genomics. 2010 Nov 29;11:674. doi: 10.1186/1471-2164-11-674 (PMC3091788; doi:10.1186/1471-2164-11-674)
Supplement: Additional file 13 — Supplementary Figure S6. Phylogenetic analysis of peroxidases from various plant species, including the poplar apoplast peroxidases. Protein sequences of 18 poplar apoplastic POXs and 30 deduced amino acid sequences for the following POXs from other species were used for phylogenetic analysis: Arabidopsis At5g06720 (A. thaliana AtPA2; Q42578), Arabidopsis At3g49120 (A. thaliana AtPCb; Q9SMU8), Arabidopsis At3g49110 (A. thaliana AtPCa; P24101), tobacco TP60 (Nicotiana tabacum; Q9XFL2), tomato TPX1 (Lycopersicon esculentum; Q07446), tomato TPX2 (L. esculentum; Q07445), cotton pod2 (Gossypium hirsutum; Q8RVW0), cotton pod3 (G. hirsutum; Q8RVP7), cotton pod4 (G. hirsutum; Q9XGV6), cotton pod6 (G. hirsutum; Q8RVP4), rice POX8.1 (Oryza sativa; O22439), rice POX22.3 (O. sativa; O22438), rice POC1 (O. sativa; Q9LKY9), Norway spruce SPI2 (Picea abies; Q9SC55), Norway spruce PX1 (P. abies; Q5W5I3), Norway spruce PX2 (P. abies; Q5W5I4), Norway spruce PX3 (P. abies; Q5W5I2), sweet potato swpa4 (Ipomoea batatas; B3SHI1), sweet potato swpb5 (I. batatas; B3SHI2), sweet potato swpb7 (I. batatas; B3SHI0), bell pepper PO2 (Capsicum annuum; A4ZCI6), poplar POD1 (Populus alba × Populus tremula var. glandulosa; Q58GF4), lombardy poplar CY26 (P. nigra; Q40949), western balsam poplar PXP3-4 (P. trichocarpa; Q43101), white poplar CWPO-C (P. alba; Q4ADU9), white poplar PO1 (P. alba: Q50KB0), white poplar PO2 (P. alba; Q08IT5), white poplar PO3 (P. alba; Q08IT6), aspen prxA1 (P. kitakamiensis; Q43055), aspen prxA3a (P. kitakamiensis; Q43049). A. thaliana L-ascorbate peroxidase 1, cytosolic (At1g07890/APX1; Q05431) was used to root the tree. [file 1471-2164-11-674-S13.PDF]

Additional file 13

File format: PDF

Title: Supplementary Figure S6

Description:
Figure S6. Phylogenetic analysis of peroxidases from various plant species, including the poplar apoplast peroxidases. Protein sequences of 18 poplar apoplastic POXs and 30 deduced amino acid sequences for the following POXs from other species were used for phylogenetic analysis: *Arabidopsis* At5g06720 (*A. thaliana* AtPA2; Q42578), *Arabidopsis* At3g49120 (*A. thaliana* AtPCb; Q9SMU8), *Arabidopsis* At3g49110 (*A. thaliana* AtPCa; P24101), tobacco TP60 (*Nicotiana tabacum*; Q9XFL2), tomato TPX1 (*Lycopersicon esculentum*; Q07446), tomato TPX2 (*L. esculentum*; Q07445), cotton pod2 (*Gossypium hirsutum*; Q8RVW0), cotton pod3 (*G. hirsutum*; Q8RVP7), cotton pod4 (*G. hirsutum*; Q9XGV6), cotton pod6 (*G. hirsutum*; Q8RVP4), rice POX8.1 (*Oryza sativa*; O22439), rice POX22.3 (*O. sativa*; O22438), rice POC1 (*O. sativa*; Q9LKY9), Norway spruce SPI2 (*Picea abies*; Q9SC55), Norway spruce PX1 (*P. abies*; Q5W5I3), Norway spruce PX2 (*P. abies*; Q5W5I4), Norway spruce PX3 (*P. abies*; Q5W5I2), sweet potato swpa4 (*Ipomoea batatas*; B3SHI1), sweet potato swpb5 (*I. batatas*; B3SHI2), sweet potato swpb7 (*I. batatas*; B3SHI0), bell pepper PO2 (*Capsicum annuum*; A4ZCI6), poplar POD1 (*Populus alba x Populus tremula* var. *glandulosa*; Q58GF4), lombardy poplar CY26 (*P. nigra*; Q40949), western balsam poplar PXP3-4 (*P. trichocarpa*; Q43101), white poplar CWPO-C (*P. alba*; Q4ADU9), white poplar PO1 (*P. alba*: Q50KB0), white poplar PO2 (*P. alba*; Q08IT5), white poplar PO3 (*P. alba*; Q08IT6), aspen prxA1 (*P. kitakamiensis*; Q43055), aspen prxA3a (*P. kitakamiensis*; Q43049). *A. thaliana* L-ascorbate peroxidase 1, cytosolic (At1g07890/APX1; Q05431) was used to root the tree.

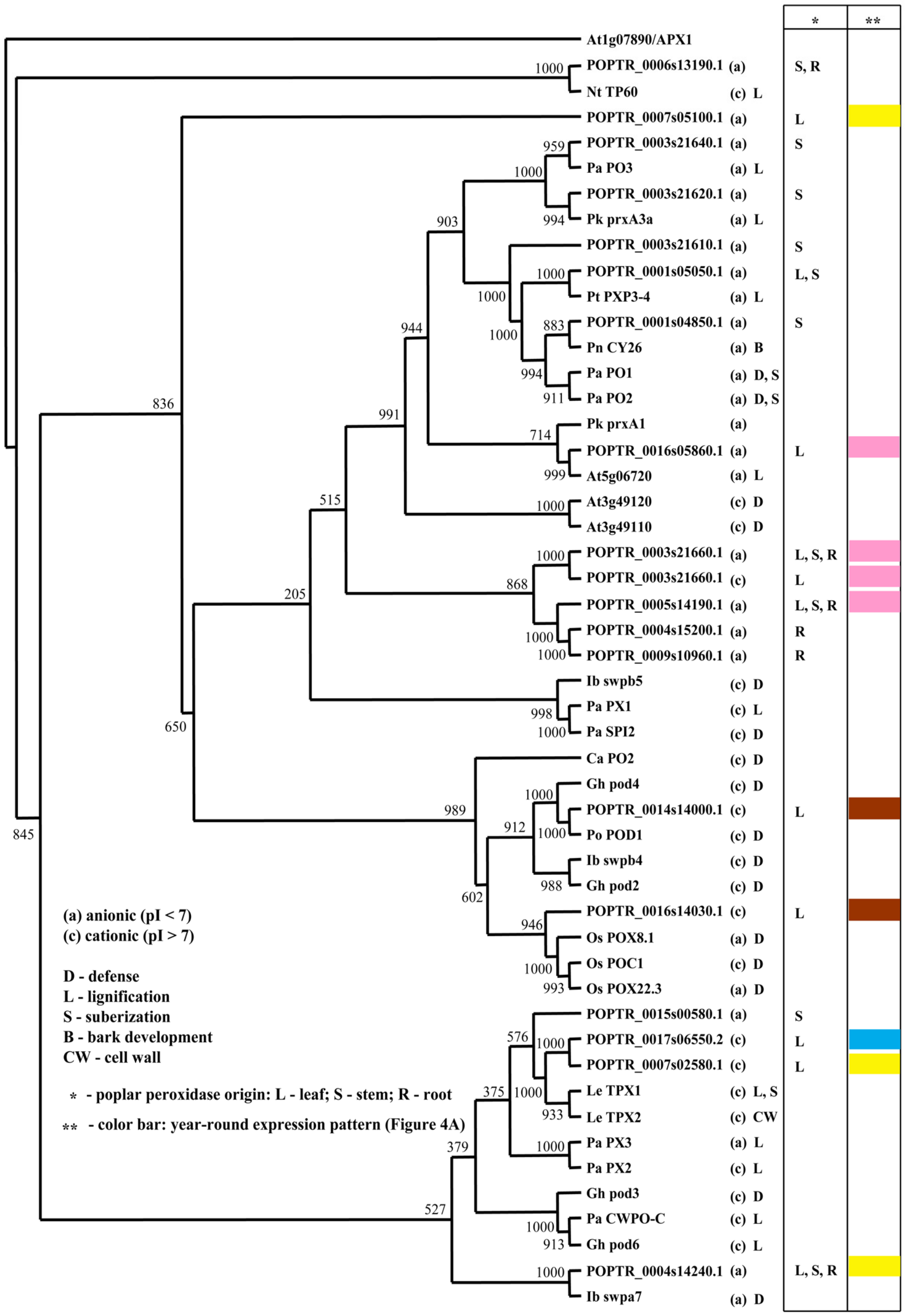

*
**
At1g07890/APX1
POPTR_0006s13190.1 (a) S, R
Nt TP60 (c) L
POPTR_0007s05100.1 (a) L
POPTR_0003s21640.1 (a) S
Pa PO3 (a) L
POPTR_0003s21620.1 (a) S
Pk prxA3a (a) L
POPTR_0003s21610.1 (a) S
POPTR_0001s05050.1 (a) L, S
Pt PXP3-4 (a) L
POPTR_0001s04850.1 (a) S
Pn CY26 (a) B
Pa PO1 (a) D, S
Pa PO2 (a) D, S
Pk prxA1 (a)
POPTR_0016s05860.1 (a) L
At5g06720 (a) L
At3g49120 (c) D
At3g49110 (c) D
POPTR_0003s21660.1 (a) L, S, R
POPTR_0003s21660.1 (c) L
POPTR_0005s14190.1 (a) L, S, R
POPTR_0004s15200.1 (a) R
POPTR_0009s10960.1 (a) R
Ib swpb5 (c) D
Pa PX1 (c) L
Pa SPI2 (c) D
Ca PO2 (c) D
Gh pod4 (c) D
POPTR_0014s14000.1 (c) L
Po POD1 (c) D
Ib swpb4 (c) D
Gh pod2 (c) D
POPTR_0016s14030.1 (c) L
Os POX8.1 (a) D
Os POC1 (c) D
Os POX22.3 (a) D
POPTR_0015s00580.1 (a) S
POPTR_0017s06550.2 (c) L
POPTR_0007s02580.1 (c) L
Le TPX1 (c) L, S
Le TPX2 (c) CW
Pa PX3 (a) L
Pa PX2 (c) L
Gh pod3 (c) D
Pa CWPO-C (c) L
Gh pod6 (c) L
POPTR_0004s14240.1 (a) L, S, R
Ib swpa7 (a) D
1000 959 1000 994 903 1000 1000 1000 883 994 911 944 991 714 999 1000 515 1000 868 1000 1000 205 998 1000 836 650 989 1000 912 1000 988 602 946 1000 993 845 576 1000 375 1000 933 379 1000 527 1000 913 1000
(a) anionic (pI < 7)
(c) cationic (pI > 7)
D - defense
L - lignification
S - suberization
B - bark development
CW - cell wall
* - poplar peroxidase origin: L - leaf; S - stem; R - root
** - color bar: year-round expression pattern (Figure 4A)
