# Supplementary material for: Apoplast proteome reveals that extracellular matrix contributes to multistress response in poplar
Source: BMC Genomics. 2010 Nov 29;11:674. doi: 10.1186/1471-2164-11-674 (PMC3091788; doi:10.1186/1471-2164-11-674)
Supplement: Additional file 14 — Supplementary Table S8. N-terminal sequencing of proteins from three spots (83, 87, and 88) on leaf apoplast 2-D gels. Letters correspond to amino acids. Dashes indicate undetermined residues. [file 1471-2164-11-674-S14.PDF]

Additional file 14

File format: PDF

Title: Supplementary Table S8

Description:
Table S8. N-terminal sequencing of proteins from three spots (83, 87, and 88) on leaf apoplast 2-D gels. Letters correspond to amino acids. Dashes indicate undetermined residues.

Residue	Amino acid		
	Spot 83	Spot 87	Spot 88
1	A	--	A
2	G	T	T
3	I	F	F
4	A	E	E
5	I	I	I
6	Y	R	R
7	W	N	N
8	G	Q	Q
9	Q	--	--
10	N	P	T
11	N	Y	Y
12	N	T	T
13	--	V	V
14	--	W	W
15	--	A	A
