# Supplementary material for: Apoplast proteome reveals that extracellular matrix contributes to multistress response in poplar
Source: BMC Genomics. 2010 Nov 29;11:674. doi: 10.1186/1471-2164-11-674 (PMC3091788; doi:10.1186/1471-2164-11-674)
Supplement: Additional file 15 — Supplementary Table S9. List of primers that were used for qRT-PCR analyses. [file 1471-2164-11-674-S15.PDF]

Additional file 15

File format: PDF

Title: Supplementary Table S9

Description:
Table S9. List of primers that were used for qRT-PCR analyses.

Experiments	Name	Gene ID	Protein ID	Forward and reverse primers (5' - 3')	Amplicon size (bp)	Tm (ºC)
**Confirmation of microarray data**	Dehydration stress-induced protein	estExt_fgenesh4_pg.C_LG_VII0502 (POPTR_0007s05650.1)	819822	F-CCTCCTCCTCCTTTCCTTCTCCTCCATAG	163	70
				R-CTCAAGATCTGGGACCTCCACGCCATAACC		75
	Thaumatin-like protein	grail3.0020019002 (POPTR_0018s10480.1)	669475	F-CGTTTTTCTTACTTATCGTTGTTTCCCTGC	234	68
				R-GGCCCCATCGAAAATGCAGTTGGTCCGTC		79
	Cationic peroxidase 1	estExt_fgenesh4_pg.C_LG_XVI1240 (POPTR_0016s14030.1)	825400	F-GCATCAGTGCTTTTGGACGGTGGAG	238	71
				R-CATCGCTAACACTTCCTGCTGTGGC		70
	P.trichocarpa X P.deltoides wound-responsive	estExt_fgenesh4_pg.C_LG_X1353 (POPTR_0010s16050.1)	822230	F-CGAAGAAATCTGCAATCGTATTACCTGG	188	67
				R-GTTCAGTTGCTGAACAGGATAGTTTTTAC		62
	Blight-associated protein p12	estExt_fgenesh4_pg.C_LG_VI1270 (POPTR_0006s19310.1)	819386	F-CTTATCTCCGTTGCGCATGCTGCACAAG	205	75
				R-GTTTGCATGGTTTTGGAGCTTCATTTG		69
	Alcohol dehydrogenase 2	estExt_fgenesh4_pg.C_LG_II0662 (POPTR_0002s07290.1)	816232	F-CACCGTCTGCCATATTGGGTCTGTTG	207	71
				R-CTCGAAGCCCCTGATAACCTTGCTCCC		73
	Phenylcoumaran benzylic ether reductase	estExt_fgenesh4_pm.C_LG_II0164 (POPTR_0002s03580.1)	830063	F-CTTCTTTGCTGCATATTATCTCCCCACATTG	230	70
				R-ATAGATCAATAAGCTCATTGAATGAGTAGG		61
	Ubiquitin	eugene3.00111099 (POPTR_0011s13770.1)	569085	F-CGATAATGTGAAGGCCAAAATTCAG	278	65
				R-GGTCAGGGGGTATTCCTTCCTTGTC		68
**Water stress**	Phenylcoumaran benzylic ether reductase	estExt_fgenesh4_pm.C_LG_II0164 (POPTR_0002s03580.1)	830063	F-CATCTCCTGCTCTCCAGTTATTAATG	96	58
				R-ACCTCCTTGACTTGGACAAACAC		58
	Isoflavone reductase related protein	gw1.IX.1737.1 (POPTR_0009s12090.1)	201272	F-CCATCTCATTCAACGATCTTGTG	80	58
				R-CTCCTCGGGAATGTAAATCTTTTC		58
	Alcohol dehydrogenase 2	estExt_fgenesh4_pg.C_LG_II0662 (POPTR_0002s07290.1)	816232	F-TGCAAGATCCACCTGCTACATG	79	60
				R-AAACCAGAACCAAAAGCCATTG		59
	Alcohol dehydrogenase	fgenesh4_pg.C_scaffold_70000003 (POPTR_0005s06140.1)	782655	F-TGGCGTGCCGAACAAAG	108	59
				R-TCTGAGCGCGGCTTATAGTTTC		60
	(-)-isopiperitenol dehydrogenase	grail3.0005000301 (POPTR_0015s07660.1)	666348	F-TGAGACCAATATGAGCTTGAAAGG	62	58
				R-GCACAGCATCTGCCACATG		58
	P.trichocarpa X P.deltoides wound-responsive	estExt_fgenesh4_pg.C_LG_X1353 (POPTR_0010s16050.1)	822230	F-AGTCAGGATGCCTTCAATGAGTATC	76	59
				R-CACAGGCAGGCAAAAACTTG		58
	Populus x generosa pop3 peptide	estExt_Genewise1_v1.C_LG_X0701 (POPTR_0010s16030.1)	723969	F-TTCGGCCGGGCTATTTC	110	58
				R-TTCACATAGCACAGCACAAACAAG		59
	Unknown protein	estExt_fgenesh4_pg.C_LG_I0347 (POPTR_0001s05560.1)	814847	F-GATGGAACTGTGTCCTTGGAATC	85	58
				R-TCATGCTCTGGCCTGACTTG		59
	Cysteine-rich repeat secretory protein 38	estExt_Genewise1_v1.C_LG_VII1106 (POPTR_1698s00200.1)	718495	F-GCCCCTATGAATCCAACCTAAAC	80	59
				R-TGAACCCAGACCAAATCCTGTAC		59

Table S9. continued

	Dehydration stress-induced protein	estExt_fgenesh4_pg.C_LG_VII0502 (POPTR_0007s05650.1)	819822	F-GGCCATGGTTGGTACGTTAACTAC	85	60
				R-GCTCAATCTCGAACTTCTTTTGTG		58
	Thaumatin-like protein	grail3.0020019002 (POPTR_0018s10480.1)	669475	F-TCGTTGTTTCCCTGCTTCCT	63	58
				R-GAAAGTAGCTGCGTTGCTTAAGG		58
	Pathogenesis-related protein 8	estExt_fgenesh4_pm.C_LG_XI0062 (POPTR_0012s01160.1)	233978	F-CTCGTGTCATCATCTCATGTATCCT	83	58
				R-TCAATTCAAGTACAAGTACACAGTCACA		58
	Acidic class III chitinase	estExt_Genewise1_v1.C_1970084 (POPTR_0015s05990.1)	746640	F-GAGCAAGCAGTATGACAATGGGTAT	98	59
				R-GGAATCAGGAGCACACAGCAT		59
	Class IV chitinase	gw1.142.209.1 (POPTR_0013s12870.1)	270686	F-TTACTACGGCCGAGGACCAA	60	59
				R-TCTCCGGCAGGTCCATAATT		58
	NtPRp27	eugene3.00012396 (POPTR_0001s30680.1)	549955	F-CAAGGCCAAGTATGGAAAATAGAAG	83	58
				R-TGATGCGAGCTGCTAAAAGTGT		59
	Cationic peroxidase 1	estExt_fgenesh4_pg.C_LG_XVI1240 (POPTR_0016s14030.1)	825400	F-GCAGGAAGGCCAATTGAATACT	67	58
				R-CACACACATAAACAAGAGCTGCAA		59
**Pathogen challenge assay**	Cyclin-dependent kinase 2 (CDC2)	grail3.0056004702 (POPTR_0004s14080.1)	648419	F-CTTCTAAGGATTTGGCAACTGTAGTTCCA	110	63.3
				R-AGCACTCCTGGCAGTAATTCTTTTAGT		63.3
	Glyceraldehyde-3-phosphate dehydrogenase (Gapdh)	estExt_fgenesh4_pg.C_LG_X0484 (POPTR_0010s06560.1)	821843	F-AGGCTTTAGATGATGTGCGAAGTTTGGA	172	66.5
				R-TGGTAGAGGAGAGAGAGCGGGA		66.4
	Ubiquitin/ribosomal protein 27a (Ubi)	estExt_Genewise1_v1.C_LG_II1812 (POPTR_0001s06260.1)	710530	F-ACCAAGCCCAAGAAGATCAAGCA	131	65.1
				R-CCAGCACCGCACTCAGCA		65
	Eukaryotic initiation factor 4A-2 (eIF4)	estExt_Genewise1_v1.C_LG_VI0160 (POPTR_0006s24220.1)	716781	F-TGGGGCCTCTATTTAGCATGGAT	88	64
				R-CTGCACCCGAAATGGGATTGACC		65.3
	Actin (Act1)	estExt_fgenesh4_kg.C_LG_I0082 (POPTR_0001s31700.1)	813612	F-CCCATTGAGCACGGTATTGT	131	60.4
				R-TACGACCACTGGCATACAGG		63.1
	Blight-associated protein p12	estExt_fgenesh4_pg.C_LG_VI1270 (POPTR_0006s19310.1)	819386	F-GCATTGTATCATGCCTTATCTCCGTTGC	156	66
				R-ATGCTGCTCCACCATTCCACAGA		66
	Acidic class III chitinase	estExt_Genewise1_v1.C_1970084 (POPTR_0015s05990.1)	746640	F-CTCTGGAGCAAGCAGTATGACAATGG	89	66
				R-ACAGCATGGGGAAGAGCAGG		66
	Thaumatin-like protein	grail3.0020019002 (POPTR_0018s10480.1)	669475	F-TTCAGTCCAGTATCAGGCAACTGC	189	66
				R-GCACCTCTGCTTGAAAAACCTAGAGTAAT		66
	Cationic peroxidase 1	estExt_fgenesh4_pg.C_LG_XVI1240 (POPTR_0016s14030.1)	825400	F-GGACGGTGGAGAAAAGACAGCAC	142	66
				R-GGTCCCGAGCAGCAACAGATAAGATG		66
	Cysteine-rich repeat secretory protein 38	estExt_Genewise1_v1.C_LG_VII1106 (POPTR_1698s00200.1)	718495	F-AGATGGAGCTAGGAGAATCAACCAAG	151	65
				R-CCACTGACAACTCTGCCACCTTC		66
