# Supplementary material for: Apoplast proteome reveals that extracellular matrix contributes to multistress response in poplar
Source: BMC Genomics. 2010 Nov 29;11:674. doi: 10.1186/1471-2164-11-674 (PMC3091788; doi:10.1186/1471-2164-11-674)
Supplement: Additional file 16 — Supplementary Figure S7. Using qRT-PCR, the transcript stability of potential internal control genes was determined using leaf tissues of the hybrid poplar clone NM6 (P. nigra X P. maximowiczii) challenged with the isolates of M. medusae f. sp. deltoidae (Mmd) and M. laricipopulina (Mlp). [file 1471-2164-11-674-S16.PDF]

Additional file 16

File format: PDF

Title: Supplementary Figure S7

Description:
Figure S7. Using qRT-PCR, the transcript stability of potential internal control genes was determined using leaf tissues of the hybrid poplar clone NM6 (*P. nigra* X *P. maximowiczii*) challenged with the isolates of *M. medusae* f. sp. *deltoidae* (Mmd) and *M. laricipopulina* (Mlp).

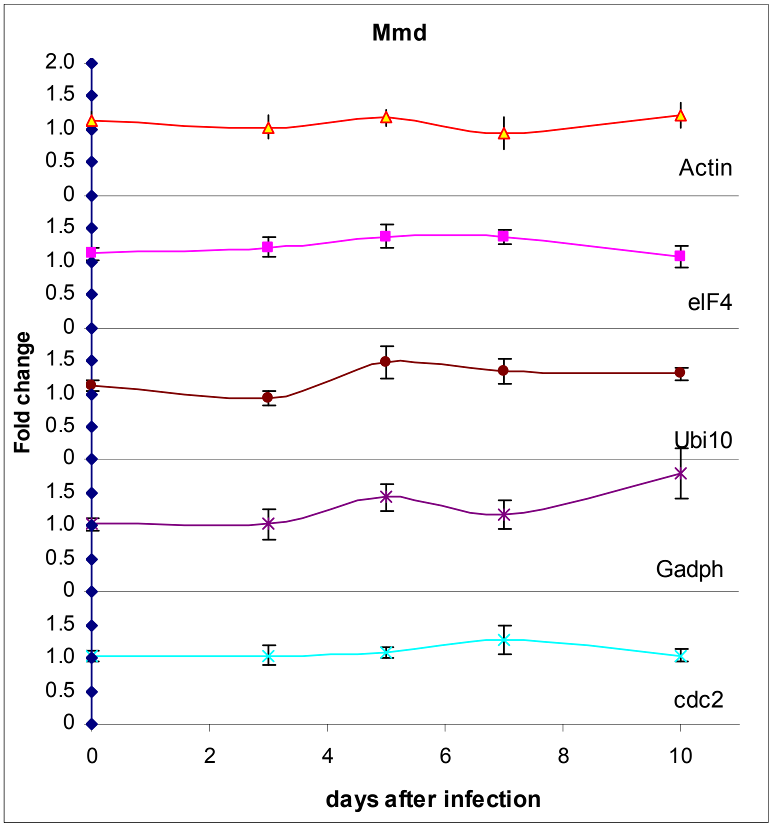
Mmd
2.0
1.5
1.0
0.5
0
Actin
elF4
Ubi10
Gadph
cdc2
Fold change
0
2
4
6
8
10
days after infection

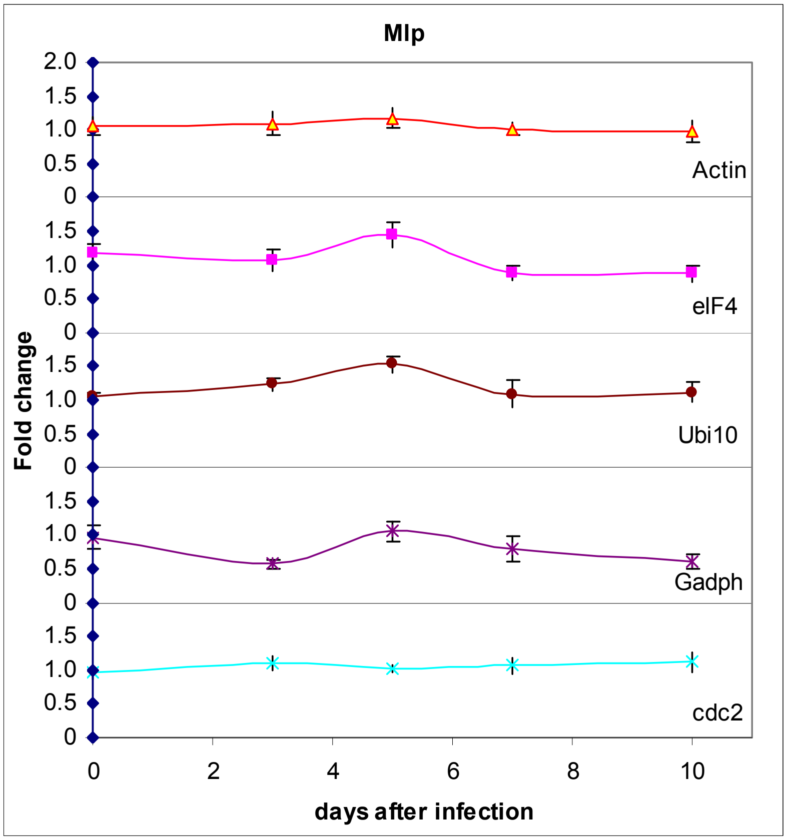
Mlp
2.0
1.5
1.0
0.5
0
Actin
elF4
Ubi10
Gadph
cdc2
Fold change
0
2
4
6
8
10
days after infection
